# Ligand Rigidity Steers the Selectivity and Efficiency of the Photosubstitution Reaction of Strained Ruthenium Polypyridyl Complexes

**DOI:** 10.1021/jacs.3c03543

**Published:** 2023-06-09

**Authors:** Matthijs L. A. Hakkennes, Michael S. Meijer, Jan Paul Menzel, Anne-Charlotte Goetz, Roy Van Duijn, Maxime A. Siegler, Francesco Buda, Sylvestre Bonnet

**Affiliations:** † Leiden Institute of Chemistry, Leiden University, P.O. Box 9502, Leiden 2300 RA, The Netherlands; ‡ Department of Chemistry, Johns Hopkins University, 3400 N Charles Street, Baltimore, Maryland 21218, United States

## Abstract

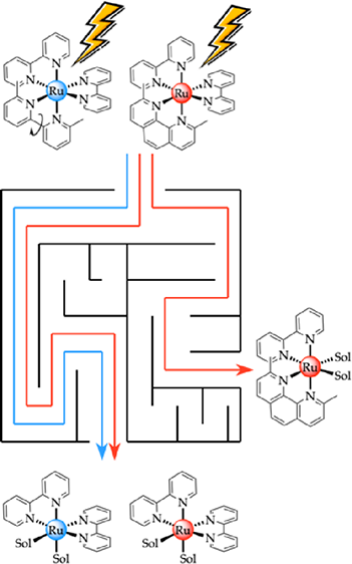

While photosubstitution reactions in metal complexes are usually thought of as dissociative processes poorly dependent on the environment, they are, in fact, very sensitive to solvent effects. Therefore, it is crucial to explicitly consider solvent molecules in theoretical models of these reactions. Here, we experimentally and computationally investigated the selectivity of the photosubstitution of diimine chelates in a series of sterically strained ruthenium(II) polypyridyl complexes in water and acetonitrile. The complexes differ essentially by the rigidity of the chelates, which strongly influenced the observed selectivity of the photosubstitution. As the ratio between the different photoproducts was also influenced by the solvent, we developed a full density functional theory modeling of the reaction mechanism that included explicit solvent molecules. Three reaction pathways leading to photodissociation were identified on the triplet hypersurface, each characterized by either one or two energy barriers. Photodissociation in water was promoted by a proton transfer in the triplet state, which was facilitated by the dissociated pyridine ring acting as a pendent base. We show that the temperature variation of the photosubstitution quantum yield is an excellent tool to compare theory with experiments. An unusual phenomenon was observed for one of the compounds in acetonitrile, for which an increase in temperature led to a surprising decrease in the photosubstitution reaction rate. We interpret this experimental observation based on complete mapping of the triplet hypersurface of this complex, revealing thermal deactivation to the singlet ground state through intersystem crossing.

## Introduction

Ruthenium(II) polypyridyl complexes exhibit great potential as molecular machines,^1,2^ biological imaging agents,^3−5^ or prodrugs for photodynamic therapy (PDT) or photoactivated chemotherapy (PACT).^6−16^ For some of them, visible light irradiation leads to the photosubstitution of one of the ligands by surrounding solvent molecules. In such photosubstitutionally active ruthenium complexes, absorption of a visible photon leads to excitation of a nonbonding (t_2g_) electron located on the metal into an antibonding orbital (π*) situated on one of the polypyridyl ligands, thereby forming a singlet metal-to-ligand charge transfer-excited state (^1^MLCT). Within less than 100 fs, this state quickly transforms into the corresponding triplet state (^3^MLCT) due to the efficient spin-orbit coupling of the ruthenium heavy atom.^17,18^ In complexes where the ligand field splitting energy is not too high, thermal activation of the ^3^MLCT state may lead to the formation of the nonluminescent triplet metal-centered state (^3^MC), in which the electron previously in the π* of the polypyridyl ligands has been promoted into an antibonding (e_g_*) orbital involving the metal. Occupying the ^3^MC states leads to elongation of the ligand–metal bond distance, making it more susceptible to substitution by solvent molecules than in the ground state. A series of studies showed that different classes of ^3^MC states can be identified: some called ^3^MC_trans_ states, in which the upper (e_g_*) single-occupied molecular orbital (SOMO) is *d*_z2_-like, and some called ^3^MC_cis_ states, where the antibonding SOMO is of *d*_x2–y2_ character. Both states exhibit different energy barriers from the ^3^MLCT state, which predicts different photoreactivities^19−23^ and suggests that excited state fine-tuning could lead to more efficient PACT compounds.^24^

Typically, modification of the ligands bound to ruthenium can steer the photophysical and photochemical properties of these complexes to obtain enhanced photosubstitution efficiency. A commonly used method follows the energy gap law and aims at lowering the ^3^MLCT-^3^MC energy gap for a faster population of the ^3^MC state(s).^24,25^ This strategy can be achieved by introducing steric strain on the dissociating ligand, thereby lowering the ligand field splitting energy of the complex, which stabilizes the ^3^MC state relative to the ^3^MLCT state.^26−32^ Alternatively, it is, instead, possible to destabilize the ^3^MLCT state via electronic effects.^32,33^ A more recent strategy has also shown to lead to photosubstitutionally active ruthenium polypyridyl complexes: very low-lying ^3^MLCT states lead to complexes that do not follow the energy gap law^34^ and that perform photosubstitution even under near-infrared light irradiation.

A less explored strategy for the fine-tuning of the triplet hypersurface is to play on the rigidity of the ligands. For example, the 2,2′-bipyridine scaffold (bpy) is “nonrigid” because the two pyridyl rings can rotate around the central C_2_–C_2’_ bond, while its analogue 1,10-phenanthroline (phen), which is electronically very similar, is much more “rigid” because such rotation is hindered by the two carbon atoms C_5_ and C_6_ that fuse the two pyridyl rings together. Generally, the photosubstitution of strained ruthenium polypyridyl complexes bearing three bidentate ligands leads to the selective expulsion of the ligand that puts the most strain on the complex. However, ruthenium polypyridyl complexes have also been described showing selective photosubstitution of a nonhindered ligand, or a completely unselective photosubstitution.^35−37^ It has been postulated by Sauvage et al. that, apart from steric strain, the degree of rotational freedom between the two metal-binding nitrogen atoms of these bidentate ligands, i.e., their rigidity, may also play a role in determining the selectivity of these photoreactions.^35^ They showed that irradiation of [Ru(bpy)_2_(dpphen)](PF_6_)_2_ (dpphen = 2,9-diphenyl-1,10-phenanthroline) in acetonitrile led to the expulsion of the less rigid bpy ligands, rather than the sterically demanding, but rigid, dpphen. However, the exact role played by the rigidity of these chelates in the photosubstitution reaction has not been rationalized yet.

**Scheme 1:**
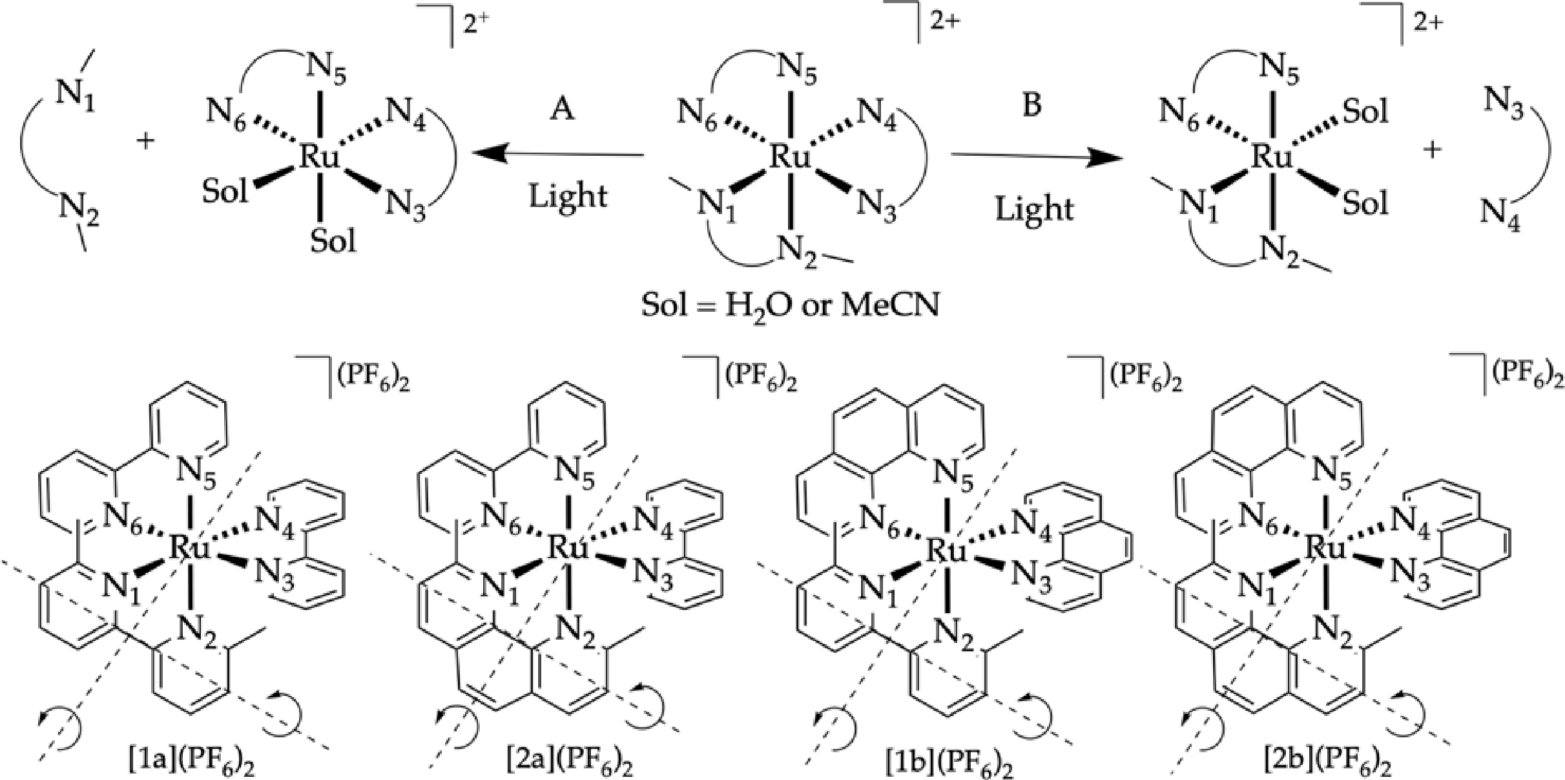
Two Possible Photosubstitution Reactions When Complexes [**1a**–**2b**](PF_6_)_2_ Are Irradiated with Visible Light in Acetonitrile (Sol = CH_3_CN) or Water (Sol = H_2_O) Reaction A represents the photosubstitution of the sterically hindering ligand (dmbpy or dmphen), and reaction B is the photosubstitution of one of the nonstraining ligands (bpy or phen).

In this work, we systematically investigated the influence of the rigidity of bipyridine-like bidentate chelates on the selectivity and efficiency of their photosubstitution in ruthenium(II) tris-diimine complexes, and this in two different solvents, water and acetonitrile. For the first time, we combined experiments with DFT modeling to compare theoretical predictions with experimental measurements. The four known ruthenium complexes [Ru(bpy)_2_(dmbpy)]^2+^ ([**1a**]^2+^, dmbpy = 6,6′-dimethyl-2,2′-bipyridine), [Ru(phen)_2_(dmbpy)]^2+^ ([**1b**]^2+^), [Ru(bpy)_2_(dmphen)]^2+^ ([**2a**]^2+^, dmphen = 2,9-dimethyl-1,10-phenanthroline), and [Ru(phen)_2_(dmphen)]^2+^ ([**2b**]^2+^) were re-synthesized (see the Supporting Information), each comprising one sterically demanding ligand substituted ortho to the metal-bound nitrogen atoms by a methyl group (dmbpy or dmphen), and two nonstraining ligands deprived of such substituents (bpy or phen). The ligands also differed in rigidity, as can be seen in Scheme 1: bpy and dmbpy ligands are not rigid, while phen and dmphen are rigid. Bipyridines and phenanthrolines are otherwise quite comparable, and in particular, they generate ruthenium(II) complexes with similar absorption and emission wavelengths. Photosubstitution reactions were performed on all four compounds in two solvents, water and acetonitrile, to measure photosubstitution quantum yields in the same conditions. Whereas most studies used the change of luminescence with temperature to calculate the rate of thermal promotion of the ^3^MLCT to ^3^MC states, and hence the barrier of activation for photosubstitution,^38−41^ we directly measured the variation of the photosubstitution quantum efficiency with temperatures for two complexes of the series in water and acetonitrile.^42^ These measurements revealed that the solvent significantly affected not only the selectivity and photosubstitution quantum efficiency at room temperature but also its activation barriers.^43^

As these systems were too large for long-time-scale dynamic calculations up to the nanosecond timescale, static DFT was used to compare the photochemistry of [**1a**]^2+^ and [**2a**]^2+^.^19,20,23,44−47^ One of the main difficulties in simulating the dissociation of bidentate ligands statically is the unknown angle from which the solvent approaches the compound, and the low symmetry of these compounds when one coordination bond is broken. To date, most theoretical studies of photosubstitution reactions in ruthenium complexes have hence not involved any explicit solvent molecules in the model, which has prevented to compare experimental data to theoretical predictions. As it seemed highly improbable that we would understand solvent effects in photosubstitution reactions without modeling explicit solvent molecules, we developed a new DFT workflow that included 1–2 explicit solvent molecules (H_2_O or CH_3_CN). This workflow enabled us to identify the minimum energy pathway for photosubstitution of either one or the other diamine chelate, using two reaction coordinates that are directly involved in the photodissociation mechanism. We show that it is possible to compare experimental photosubstitution quantum yields to theoretical activation energies. Finally, we found a unique deactivation pathway for complex [**2a**]^2+^ in acetonitrile, which showed experimentally an unexpected behavior, i.e., a decrease in photosubstitution quantum yield when the temperature increased.

## Results

### Experimental Determination of the Photochemical Selectivity in Water and Acetonitrile

**Figure 1:**
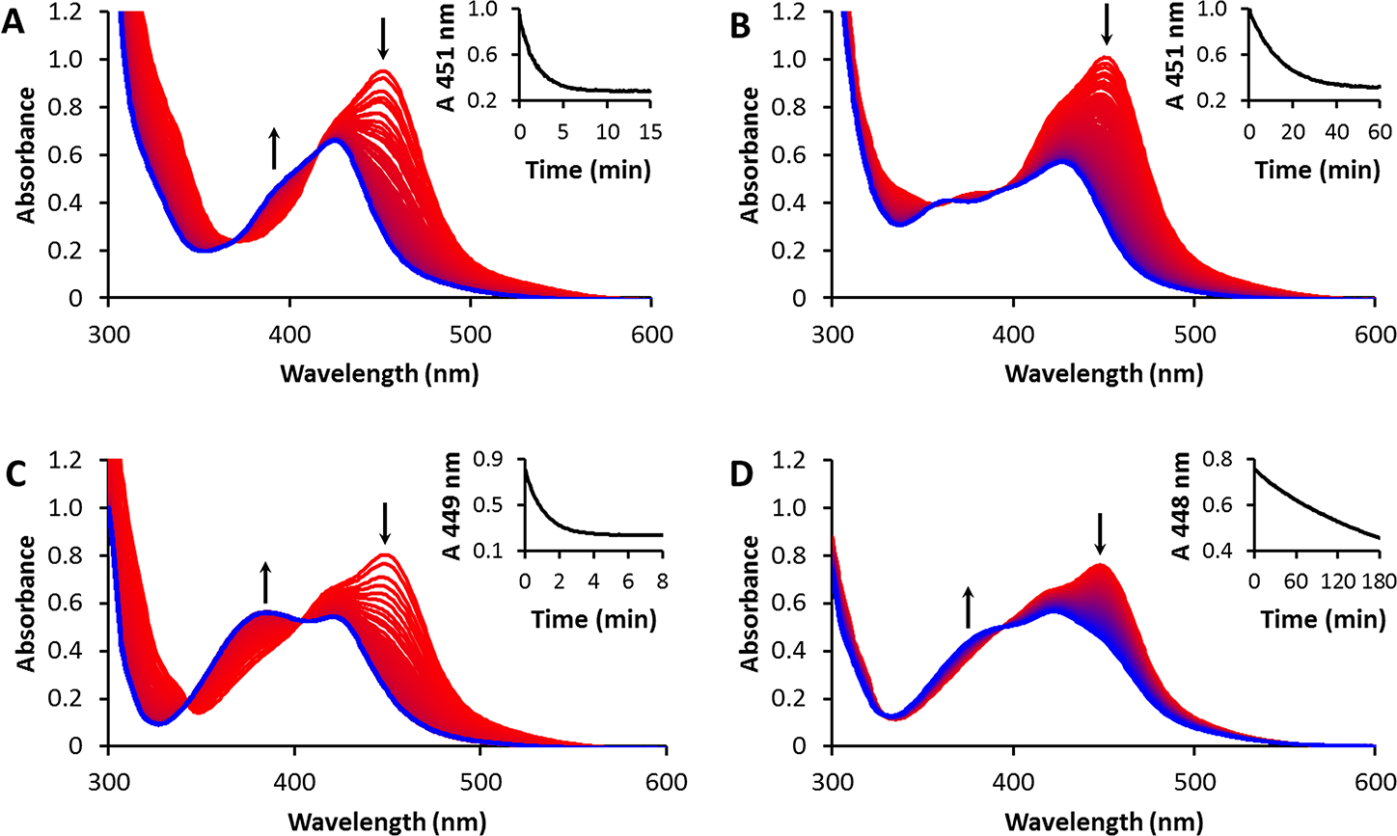
Evolution of the UV–vis absorption spectra of a solution of (A) [**1a**](PF_6_)_2_, (B) [**2a**](PF_6_)_2_, (C) [**1b**](PF_6_)_2_, and (D) [**2b**](PF_6_)_2_ in CH_3_CN upon irradiation with a 413 nm LED (5.38 × 10^–8^ mol photons·s^–1^) under N_2_ at 298 K. Conditions: (A) 15 min, 94 μm; (B) 60 min, 73 μm; (C) 15 min, 50 μm; (D) 180 min, 48 μm.

The photosubstitution reaction of the four compounds was first studied in acetonitrile. A clear ^1^MLCT absorption band around 450 nm was seen for all investigated complexes. [**1a**](PF_6_)_2_ was irradiated by violet light (413 nm). Photosubstitution was accompanied by a hypsochromic shift in the ^1^MLCT absorption band from 451 to 425 nm, with two isosbestic points at 369 and 416 nm (Figure 1A) suggesting the reaction occurred in a single step. Only the sterically demanding dmbpy ligand was substituted, as confirmed by mass spectrometry. These observations were in agreement with the selectivity previously reported by Glazer et al.^27^ In the same conditions, a similar photosubstitution reaction was observed for compounds [**1b**](PF_6_)_2_ and [**2b**](PF_6_)_2_. The ^1^MLCT absorption band shifted from 449 to 420 nm, accompanied by two isosbestic points at 342 and 404 nm (Figure 1C) for compound [**1b**](PF_6_)_2_, whereas irradiation of compound [**2b**](PF_6_)_2_ led to an absorption band shift from 448 to 420 nm, with isosbestic points at 329 and 394 nm (Figure 1D). Photosubstitution of the latter required a considerably longer time, however, and it could not be achieved within 3 h, even if the data were still consistent with a single-step reaction. Mass spectrometry and monitoring by ^1^H-NMR in CD_3_CN by white light (λ > 400 nm, see Figures S12–S15) concluded that the photosubstitution reaction selectively cleaved off the sterically demanding ligand dmphen.

**Figure 2:**
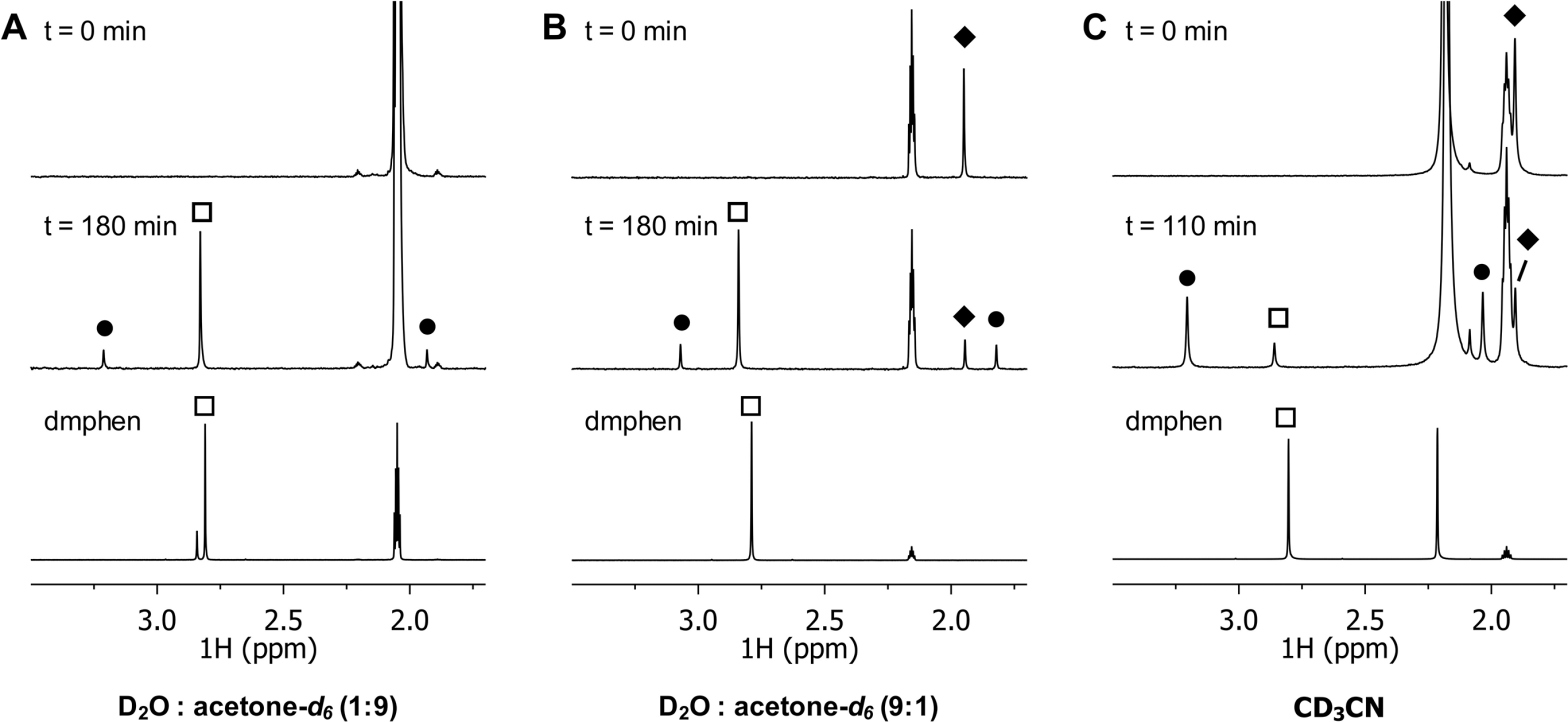
Evolution of the ^1^H NMR spectrum (δ = 3.5–1.7 ppm) of [**2a**](PF_6_)_2_ (tilted square solid) under irradiation with white light in D_2_O:acetone-*d*_6_ (1:9 v/v (A) or 9:1 v/v (B) or CD_3_CN (C)) at 298 K under N_2_, also showing the ^1^H NMR spectrum of the photoproduct 2,9-dimethyl-1,10-phenanthroline (empty squares). Peaks belonging to the photoproduct cis-[Ru(bpy)(dmphen)(Sol)_2_]^2+^ are labeled as circle solid. In panels A and C, the peak belonging to the starting compound [**2a**](PF_6_)_2_ is (partially) obscured by the residual solvent signals (δacetone-*d*_6_ = 2.05 ppm; δCD_3_CN = 1.94 ppm).

Interestingly, a different selectivity was found upon irradiation of [**2a**](PF_6_)_2_ by violet light. Similar to the other three compounds, the absorption resulted in a blue shift of the ^1^MLCT band (Figure 1B). However, no isosbestic points were found, implying that multiple reactions occurred, either sequential or in parallel. Mass spectrometry revealed the formation of four different compounds, which indicated that either the dmphen or bpy ligand was expulsed. ^1^H-NMR spectroscopy was used to follow the unselective photosubstitution of [**2a**](PF_6_)_2_ in CD_3_CN under white light irradiation (Figure 2A). The singlet at 2.80 ppm (empty squares) can be attributed to free dmphen, while bpy was identified by two doublets at 8.45 and 8.66 ppm (Figure S13). The photoproducts containing ruthenium, [Ru(bpy)_2_(CD_3_CN)_2_]^2+^ and [Ru(bpy)(dmphen)(CD_3_CN)_2_]^2+^, were identified by the doublets at 9.30 and 9.47 ppm, respectively. The ratio between the rates of the competing photosubstitution reactions was determined from the ratio of the integrals of these peaks. Photosubstitution of [**2a**](PF_6_)_2_ in acetonitrile led to a bpy:dmphen ratio of 6:1, i.e., the least hindered and least rigid bpy ligand was substituted preferentially.

The effect of the incoming ligand on the selectivity of the photosubstitution was investigated by studying the same reaction in a water-containing solution. A water:acetone 1:1 mixture was used to solubilize the poorly water-soluble hexafluorophosphate salts. Irradiation was performed by a 466 nm light source (instead of 413 nm) to stay close to the new isosbestic point. Complexes [**1a**](PF_6_)_2_, [**1b**](PF_6_)_2_, and [**2b**](PF_6_)_2_ showed no change in selectivity, as confirmed by ^1^H-NMR and mass spectrometry (see the Supporting Information). The photosubstitution reaction remained one step and remained selective toward the sterically demanding ligand. Surprisingly, however, for [**2a**](PF_6_)_2_, the selectivity ratio in water was found opposite to that in acetonitrile: dmphen photosubstitution was found preferential to bpy photosubstitution, with a bpy:dmphen ratio of 1:3. The water concentration did not influence this value, as the same bpy:dmphen ratio was found in both 1:9 (Figure 2B) and 9:1 (Figure 2C) D_2_O:acetone-*d*_6_ mixtures. The photosubstitution reaction of this compound is, therefore, highly dependent on the nature of the incoming solvent.

In principle, both the rigidity of the ligand and solvent may influence not only the selectivity of the photosubstitution reaction but also its quantum efficiency (Table 1). For compounds [**1a**](PF_6_)_2_, [**1b**](PF_6_)_2_, and [**2b**](PF_6_)_2_, however, going from acetonitrile to water:acetone mixtures had little effect on the photosubstitution quantum yield Φ. In contrast, for [**2a**](PF_6_)_2_, Φ was an order of magnitude higher in acetonitrile (0.0050), compared with water:acetone 1:1 (0.00046). As it was difficult to rationalize these results with experiments, we engaged in modeling this reaction computationally and decided to include explicit solvent molecules in the model to investigate in more details the solvent effects on this reaction.

**Table 1:** Maximum Absorption Wavelengths (λ_max_), Molar Absorption Coefficients (ε), Photosubstitution Quantum Yields in CH_3_CN (Φ_413_) and H_2_O/Acetone (Φ_466_), Photosubstitution Reactivities in CH_3_CN (ξ_413_) and H_2_O/Acetone (ξ_466_), Singlet Oxygen Generation Quantum Yields (Φ_Δ_), Phosphorescence Quantum Yields (Φ_P_), and Emission Wavelengths (λ_em_) for Complexes [**1a**–**2b**](PF_6_)_2_

complex	λ_max_ (nm)	ε_413_^a^	ε_466_^b^	Φ_413_^a^	Φ_466_^b^	ξ_413_^a^^,^^b^	ξ_466_^b^^,^^f^	Φ_Δ_^c^	Φ_P_ (λ_em_/nm)^c^
[**1a**](PF_6_)_2_	451 (9.87)	6.05	10.4	0.050	0.034	302	352	0.023^d^	3 × 10^–5^ (614)
[**2a**](PF_6_)_2_	451 (13.9)	9.08	10.5	0.0050^e^	0.00046^e^	45^e^	4.8^e^	<0.005	5 × 10^–5^ (622)
[**1b**](PF_6_)_2_	449 (15.8)	12.3	11.4	0.045	0.028	551	320	<0.005	2 × 10^–5^ (605)
[**2b**](PF_6_)_2_	448 (15.7)	12.8	12.3	0.00025	0.00023	3.2	2.8	<0.005	3 × 10^–5^ (611)

a In CH_3_CN, ε (10^3^ m^–1^·cm^–1^).

b In H_2_O/acetone (1:1), ε (10^3^ m^–1^·cm^–1^).

c In CD_3_OD, λ_exc_ = 450 nm.

d See Cuello-Garibo et al.^14^

e Based on the consumption of [**2a**](PF_6_)_2_, thus including both possible photosubstitution reactions (bpy or dmphen substitution; see Scheme 1).

f ξ_λ_ = Φ_λ_ × ε_λ_.

### DFT Study of the Photosubstitution Mechanism

**Scheme 2:**
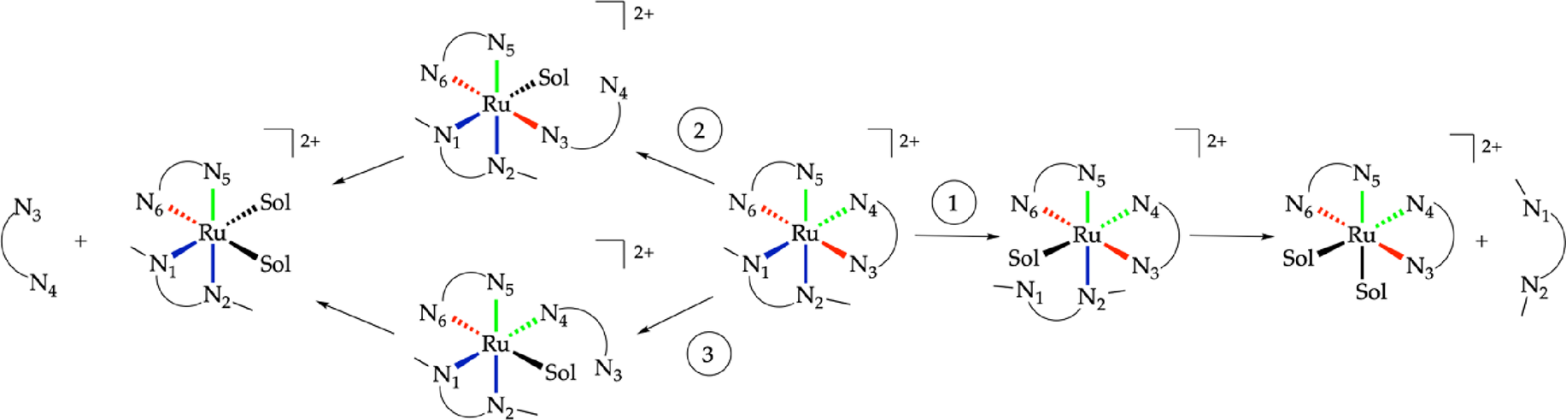
Three Possible Reaction Pathways for the Dissociation of One of the Bidentate Ligands in [**1a**]^2+^ and [**2a**]^2+^ Color code indicates bonds that are equivalent by symmetry (see main text).

The photosubstitution mechanism of polypyridyl complexes generally occurs via an intermediate state in which the dissociating ligand is still bound to the complex in a monodentate fashion (κ^1^).^48^ Since complexes [**1a**]^2+^ and [**2a**]^2+^ have three pairs of symmetrically unequivalent Ru-N bonds, the photosubstitution reaction may follow three different reaction pathways, shown in Scheme 2. In pathway 1 (blue), one of the two Ru-N bonds with the sterically hindering ligand (called N_1_) is broken first. In reaction pathway 2 (green), one of the Ru-N bonds of the bipyridines *trans* to the sterically hindering ligand (called N_4_) initially breaks. Finally, in pathway 3 (red), a Ru-N bond of the nonhindered ligand (bpy) *trans* to the nitrogen bond of the other nonhindered ligand, called N_3_, is broken first. Pathways 2 and 3, in which the bpy ligand dissociates from the complex, lead to the same final product since the two bpy ligands are equivalent by C_2_ symmetry.

While several ^1^MLCT states with similar energies can be excited with visible light, we assumed here that the question of knowing which of these states is originally occupied following photon absorption was not relevant to the pathway followed and to the nature of the dissociation product. In other words, we assumed that Kasha’s rule was valid for photosubstitution reactions. This assumption is supported by the high density of states of these complexes, which allows for quick interconversion between the different ^1^MLCT states,^49,50^ and by the very fast intersystem crossing toward ^3^MLCT states (10–100 fs for [Ru(bpy)_3_]^2+^ according to both modeling and experiments^51−54^). Meanwhile, conversion between the different ^3^MLCT states of [Ru(bpy)_3_]^2+^ localized on the different bpy ligands has also been followed using Car-Parrinello MD,^55^ trajectory surface hopping,^54^ and mixed quantum-classical approaches.^56,57^ All methods showed subpicosecond transitions of the excited electron from one bpy ligand to the other. While explicit solvation leads to a more localized character of the excited electron, it is still quite mobile and hops between different bpy ligands.^55,58,59^ Therefore, in the triplet state as well, the question of knowing which specific ^3^MLCT state is initially occupied is hypothesized to be less relevant to the substitution selectivity than further thermally activated dissociation from the different ^3^MC states.

**Figure 3:**
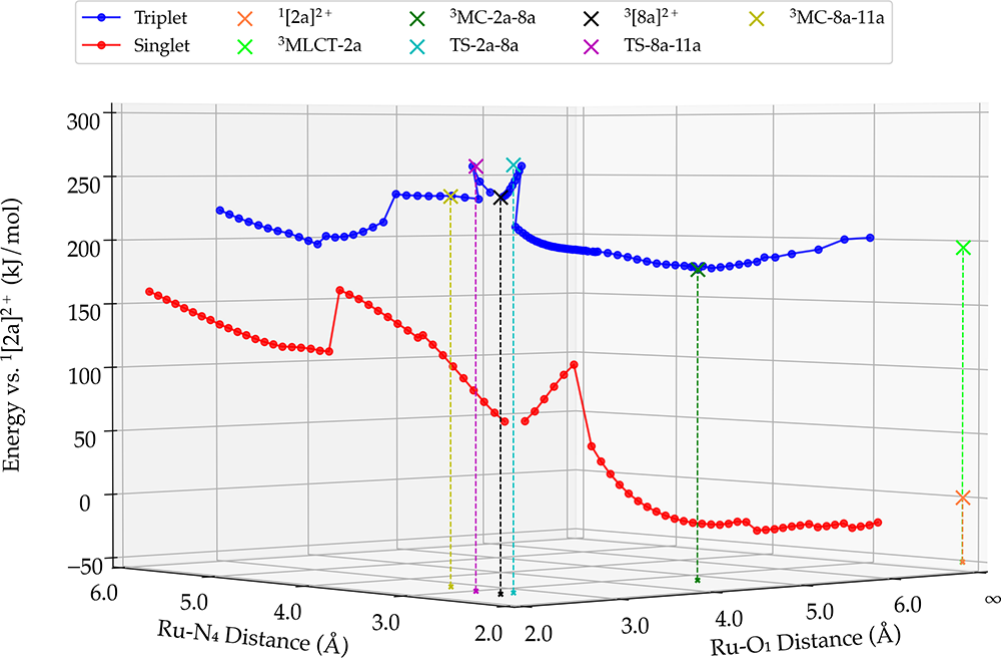
One example of a linear transit plot obtained for the singlet ground state (red) and for the triplet-excited state (blue) for pathway 3 of complex [**2a**]^2+^ in water. The Ru-O_1_ distance that was constrained during linear transit calculations is plotted on the *x*-axis, the distance involving the second step of the substitution (here, Ru-N_4_) is shown on the *y*-axis, and the energy (in kJ/mol) relative to the ground state ^1^[**2a**]^2+^ is plotted on the *z*-axis. See Schemes S4 and S5 for the formulae of all intermediates and species on the singlet and triplet hypersurface.

It is usually acknowledged that following the formation of the ^3^MLCT state, a ligand may be photosubstituted when one or several ^3^MC states can be reached thermally from one of the photochemically generated ^3^MLCT state. As the most favorable angle from which the first solvent incoming molecule would approach the elongated Ru-N bond is *a priori* unknown, it was decided to use the singlet and triplet geometries of the κ^1^ intermediate as a starting point (e.g., [**3a**]^2+^ of [**4a**]^2+^ for pathway 1 in [**1a**]^2+^, see Scheme S4). In these intermediate geometries, one solvent molecule has formed a coordination bond with ruthenium, while the ligand that will ultimately be photosubstituted is still bound in a monodentate fashion to ruthenium. From such a local minimum, the coordination bond involving the first incoming solvent molecule (Ru-Sol in Scheme 2) was then elongated step by step. For each of these steps, the Ru-Sol bond length was constrained while the rest of the geometry was optimized, either on the singlet or on the triplet hypersurface. This procedure led to a molecularly realistic initial reaction pathway for the first incoming solvent molecule called a linear transit path. The same procedure was repeated to model the cleavage of the second Ru-N bond by elongating the bond distance between ruthenium and the second nitrogen atom of the substituted chelate, which was in most pathways still bound in a κ^1^ fashion in the intermediate (e.g., Ru-N_4_ for pathway 3, see Figure 3). In both linear transit paths, when a local minimum was identified on the constrained pathway, unconstrained geometry optimization was systematically repeated near that geometry, which led to real local minima corresponding to the real intermediates of the photoreaction (Figure 3). Transition states were obtained by transition-state searches and nudged elastic band (NEB) calculations between the different local minima along these paths.

### Nomenclature

To identify and name all geometries involved in these different pathways, a systematic nomenclature was defined (both for local minima on the singlet and triplet hypersurface and transition states) according to the following principles: (i) for each pathway 1, 2, or 3, local minima corresponding to the κ^1^ intermediate structure or the final bis-aqua product of the reaction were defined according to a numbering scheme fully detailed in Scheme S4 (geometries on the singlet hypersurface) and Scheme S5 (geometries on the triplet hypersurface), and a letter that corresponded to the nature of the starting compound. For example, the dissociation of the nonstraining bpy ligand in pathway 3 of complex [**2a**]^2+^ in water led to the intermediate state [**8a**]^2+^ and the final bis-aqua product [**11a**]^2+^. Both species may exist either as a singlet or as a triplet state; (ii) an intermediate-excited triplet state that lays between two different local minima of the triplet hypersurface was named first with the nature of the state (MC, MLCT, or TS for transition states), second by the state from which the reaction starts, third by the final product of the considered reaction pathway. For example, the transition state between state ^3^[**2a**]^2+^ and ^3^[**8a**]^2+^ was named TS-2a-8a hereafter, while the local minimum that corresponds to the ^3^MC state for this pathway is named ^3^MC-2a-8a.

### Proton Shift

**Figure 4:**
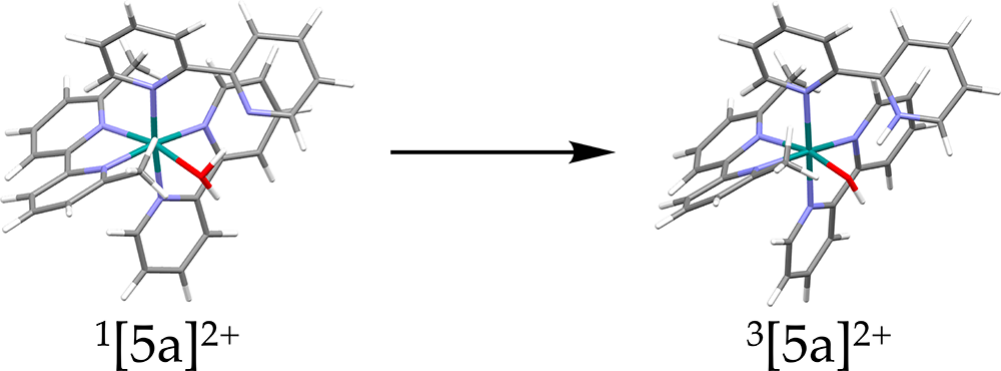
Proton shift from the coordinated water molecule in the singlet state ^1^[**5a**]^2+^ to the dissociated N atom of the κ^1^-bound bpy ligand in the triplet state ^3^[**5a**]^2+^ following pathway 1 in water. In parallel, the Mulliken charge on the ruthenium atom was increased in the triplet state ^3^[**5a**]^2+^, compared to the singlet state. This phenomenon occurred for all 3 pathways and both complexes [**1a**]^2+^ and [**2a**]^2+^ in water.

In water, both geometries ^3^[**3a**]^2+^ and ^3^[**4a**]^2+^ of pathway 1 were identified along the triplet hypersurface as ^3^MC states, as demonstrated by a spin density on the ruthenium atom of approximately 1.5. By contrast, geometries ^3^[**5a**]^2+^,^3^[**6a**]^2+^ of pathway 2, and ^3^[**7a**]^2+^,^3^[**8a**]^2+^ along pathways 3, were ^3^MLCT states, as their spin density on the ruthenium atom was around 0.8. Interestingly, all triplet geometries showed an (10-15%) increased Mulliken charge on the ruthenium atom, compared to the singlet state of the same species. In parallel, these triplet states showed a proton shift between the water molecule coordinated to ruthenium and the nearby dissociated nitrogen atom of the κ^1^-bound ligand (Figure 4). Hence, while singlet states such as ^1^[**5a**]^2+^ can be formally described as [Ru^II^(κ^1^-bpy)(bpy)(dmbpy)(OH_2_)]^2+^, their triplet analogue ^3^[**5a**]^2+^ , which is of MLCT character, better fits with the formula [Ru^III^(κ^1^-Hbpy^+^)(bpy^·–^)(dmbpy)(OH^–^)]^2+^. This role of the dissociated pyridine ring as a pendent base during photosubstitution in water had never been reported yet. To examine the effect of this proton transfer, we optimized compound ^3^[**5a**]^2+^ by constraining the transferred proton at a bonding distance of 0.96 Å to the oxygen atom of the coordinating water molecule. The obtained structure remained a ^3^MLCT state with a Mulliken spin around 0.8 but appeared 40.7 kJ/mol higher in energy compared to the hydroxo species ^3^[**5a**]^2+^ (after proton transfer). Overall, the main effect of proton transfer was of thermodynamic nature: it stabilized the intermediate ^3^[**5a**]^2+^. In acetonitrile, such proton shift can of course not occur. In this solvent, all triplet geometries ^3^[**12a**]^2+^, ^3^[**13a**]^2+^, ^3^[**14a**]^2+^, ^3^[**15a**]^2+^, ^3^[**16a**]^2+^, and ^3^[**17a**]^2+^ corresponded to ^3^MC states (Tables S14 and S15). Even though a proton transfer was not observed, the Mulliken charge was comparable to the ^3^MC states in water. A slight increase in spin density of approximately 0.2 on the ruthenium atom was observed for the ^3^MC states in acetonitrile, suggesting that the proton transfer facilitated the delocalization of the spin density over the ligand.

### Computed Mechanisms

**Figure 5:**
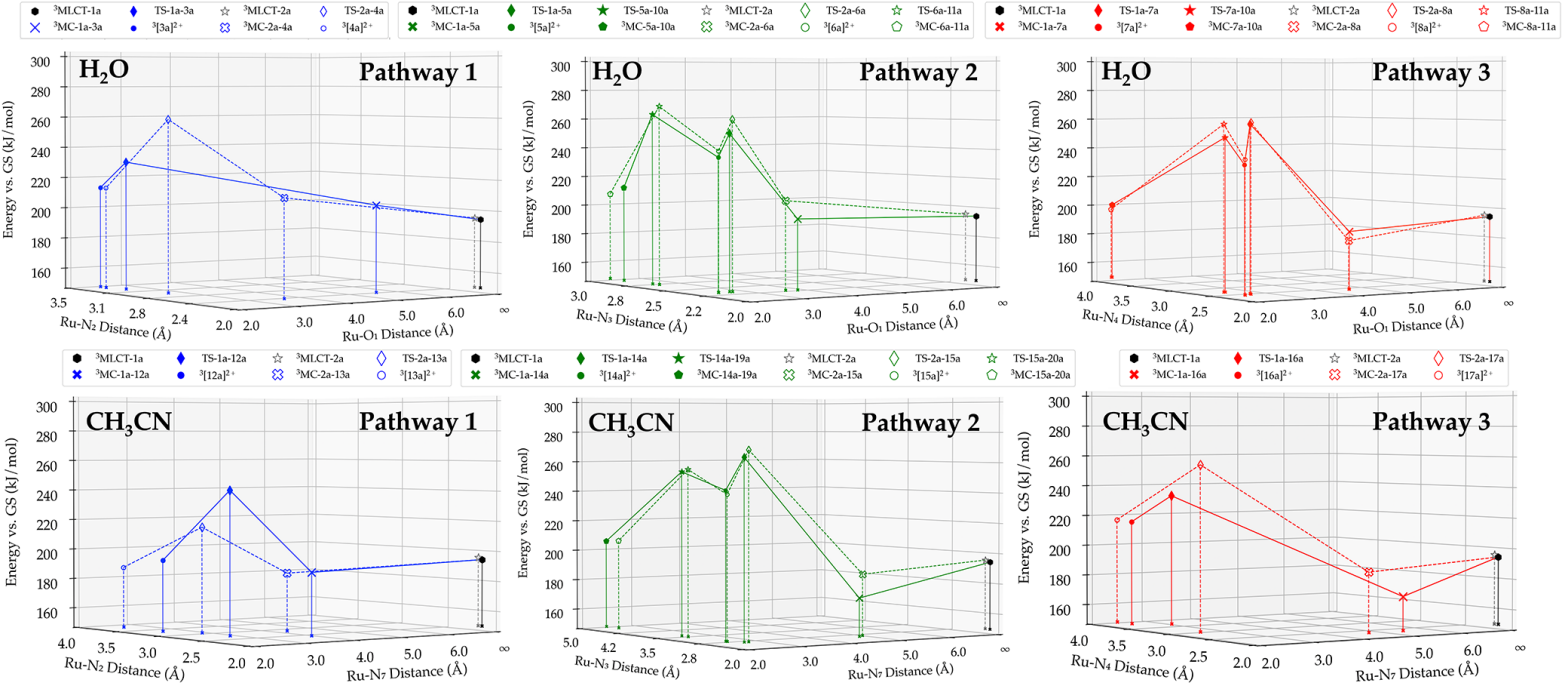
Energy vs bond distance plot for the triplet states involved in the photodissociation of a bidentate chelate for all three possible pathways shown in Scheme 2, in water and acetonitrile. The solid line and markers correspond to [**1a**]^2+^, and the dashed line and hollow markers correspond to [**2a**]^2+^. The energy of each state, plotted on the *z*-axis, is relative to the ground singlet states of [**1a**]^2+^ and [**2a**]^2+^. Level of theory: PBE/TZP/D3-BJDAMP/ ZORA /COSMO. See Schemes S4 and S5 for the formulae of all intermediates and species on the singlet and triplet hypersurface.

In both solvents, [**1a**]^2+^ and [**2a**]^2+^ showed a similar trend for each of the three pathways, as can be seen in Figure 5. All three pathways start as expected by a conversion from the ^3^MLCT to a ^3^MC state, in which a ruthenium nitrogen bond is elongated due to the occupation of an e_g_* orbital. In water, such elongation allows the incoming water molecule to bind to ruthenium and in a concerted manner transfer a proton to the nitrogen of the dissociating pyridyl ligand, to end up with a ^3^MLCT-type intermediate with a κ^1^-bound intermediate species. In pathway 1, thermal activation was required for the transitions from ^3^MLCT-1a to ^3^MC-1a-3a and ^3^MLCT-2a to ^3^MC-2a-4a. The dissociation of the sterically strained ligand was a one-step process, as no stable κ^1^-bound geometry was found. Pathway 2 required, like pathway 1, thermal activation for the ^3^MLCT-1a to ^3^MC-1a-5a and ^3^MLCT-2a to ^3^MC-2a-6a. In contrast to pathway 1, however, a two-step process was clearly found for the dissociation of the bpy ligand, as a stable κ^1^-pyridinium hydroxo intermediate was found as the local minimum. Crossing a second energy barrier was required for full dissociation to occur. In pathway 3, the ^3^MC states were found lower in energy, and a spontaneous transition from ^3^MLCT-1a to ^3^MC-1a-7a and ^3^MLCT-2a to ^3^MLCT-2a-8a could be expected. Stabilization of ^3^MC-1a-7a and ^3^MC-2a-8a was due to a π-π interaction between the dissociating bpy ligand and the coordinated dmbpy/dmphen ligand. Similar to pathway 2, a stable κ^1^-pyridinium hydroxo intermediate was found for pathway 3, and dissociation followed a two-step process. A concerted mechanism for proton transfer and Ru-O_1_ coordination bond formation was found in TS-1a-3a, TS-2a-4a, TS-1a-5a, TS-2a-6a, TS-1a-7a, and TS-2a-8a.

**Figure 6:**
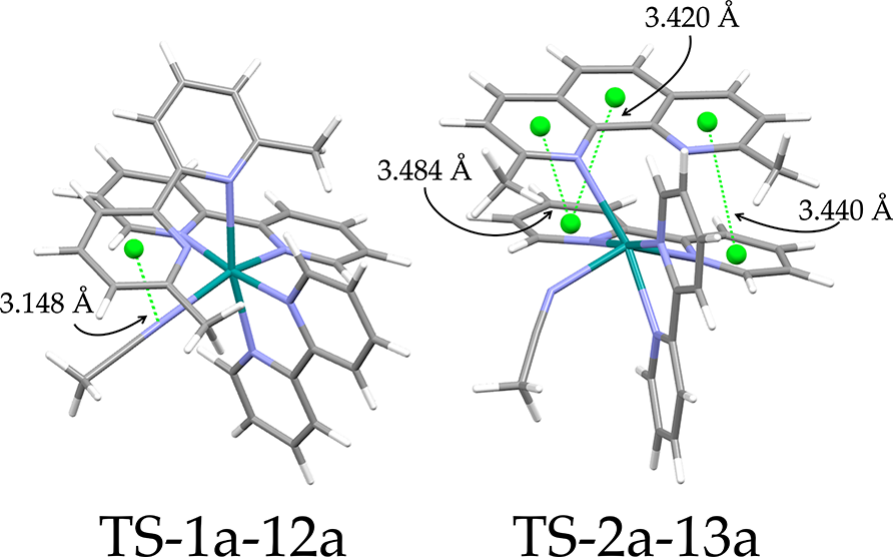
In TS-1a-12a, the noncoordinated pyridine ring of the bpy ligand can rotate around its central C_2_-C_2'_ axis without losing the coordination bond of the second nitrogen atom to ruthenium, to form a π-π interaction with the acetonitrile. The dmphen ligand cannot rotate around the analogue C_11_-C_12_ axis, and initial elongation of the Ru-N_1_ bond is favored to form a stronger π-π interaction with one of the coordinated bpy ligands.

Acetonitrile, on the other hand, cannot form a hydrogen bond or exchange a proton with the κ^1^-bound bidentate ligand. This essential difference between the two solvents had a significant effect on the ^3^MLCT-to-^3^MC state transition. The acetonitrile molecule can already form a π-π interaction with the bispyridyl ligand, whereas in water, this interaction cannot exist: the ruthenium-nitrogen bond must be elongated first before the water molecule forms a stabilizing hydrogen bond with the nitrogen atom that will become pendent in the transition state. All ^3^MC states were, thus, found at a lower energy in acetonitrile than the ^3^MLCT states. In pathway 1, the TS-2a-13a state was found at a 24.2 kJ/mol lower energy than TS-1a-12a. In TS-2a-13a, the dmphen cannot rotate around its axis to form a π-π interaction with the acetonitrile, which the dmbpy ligand in TS-1a-12a does (Figure 6, left). The greater π-system of the dmphen ligand in complex [**2a**]^2+^ favored the initial elongation of the Ru-N_1_ bond (instead of the Ru-N_2_ bond), to facilitate the formation of a stronger π-π interaction with one of the bpy ligands, which resulted in the observed difference (Figure 6, right). Similar to water, the dissociation of the straining ligand was a one-step process, and the dissociation of the bpy ligand via pathway 2 was a two-step process. In contrast, switching to acetonitrile made pathway 3 a one-step process instead of a two-step process.

### Computed Activation Barriers

From these intermediate energies, thermal activation barriers for the photosubstitution reaction could be calculated by taking the difference in energy between the lowest stable state on the triplet hypersurface and the highest transition state on each pathway. The values are shown in Table 2. According to all computed activation barriers, the most favorable pathway for complex [**1a**]^2+^ in water was pathway 1, which was characterized by an activation energy of 37.5 kJ/mol (Table 2). The elongation of the Ru-N_1_ bond in the dissociative ^3^MC state allowed the nonrigid dmbpy ligand to rotate around its C_2_-C_2'_ axis. This rotation allowed the incoming water molecule to form a hydrogen bond at a favorable angle with N_1_. Subsequently, in TS-1a-3a and at this optimal angle, a proton transfer occurred simultaneously with formation of the Ru-O_1_ bond. As the dmphen ligand in complex [**2a**]^2+^ could not rotate around its C_11_-C_12_ axis, the energy required for dissociation was considerably higher for this complex (activation barrier: 64.7 kJ/mol). Moreover, the angle between the nitrogen and water in TS-2a-4a, before proton transfer, was less optimal than that of TS-1a-3a. As expected, pathways 2 and 3 of complex [**1a**]^2+^ in water had notably higher activation energies as dissociation of either bpy ligand does not alleviate any strain on the complex. The higher activation of pathway 3 can also be rationalized by the nature of its ^3^MC state. In this dissociative state, the occupation of the antibonding orbital leads to elongation of two bonds that are involved with the nonsteric bpy ligands (Ru-N_3_ and Ru-N_6_), whereas some strain relief still occurs in the ^3^MC state of pathway 2 due to elongation of the Ru-N_1_ bond. These calculated activation barriers hence predict that only dmbpy would dissociate upon light activation of [**1a**]^2+^, which is indeed what was experimentally observed.

**Table 2:** Thermal Activation Energy on the Triplet Hypersurface According to DFT Calculations for Reaction Pathways 1–3 in Water^a^

complex	pathway	ligand dissociating	state (*x*)	state (*y*)	state (*z*)	Δ*E*_1_ (*x* – [a]) (kJ/mol)	Δ*E*_2_ (*z* – *y*) (kJ/mol)	Δ*E*_3_ (*x* – *y*) (kJ/mol)
[**1a**]^2+^	1	dmbpy	TS-1a-3a	[**3a]**^2+^		37.5		
	2	bpy	TS-1a-5a	[**5a]**^2+^	TS-5a-10a	58.3	26.8	15.3
	3	bpy	TS-1a-7a	[**7a]**^2+^	TS-7a-10a	71.3	17.0	25.8
								
[**2a**]^2+^	1	dmphen	TS-2a-4a	[**4a]**^2+^		64.7		
	2	bpy	TS-2a-6a	[**6a**]^2+^	TS-6a-10a	66.5	28.1	20.2
	3	bpy	TS-2a-8a	[**8a**]^2+^	TS-8a-10a	78.0	22.3	23.5

a [a] Energetically, the lowest on the triplet state.

For [**2a**]^2+^, the energy of activation in water following pathway 3 was disfavored (78.0 kJ/mol), compared to [**1a**]^2+^, due to the stabilization of the ^3^MC-2a-8a state. Analysis of these states shows us that in both structures, the bpy ligand has rotated to form a π-π interaction with the straining ligand. Since dmphen has a greater conjugated system, the ^3^MC-2a-8a is more stabilized than ^3^MC-1a-7a. Interestingly, the rigidity of the dmphen ligand causes the activation energy of [**2a**]^2+^ pathway 1 in water to rise, compared to [**1a**]^2+^. As a result, pathways 1 and 2 showed similar energy of activation of 64.7 and 66.5 kJ/mol, respectively. This would predict a bpy:dmphen ratio of 1:1 for the photosubstitution reaction. However, it was also found that it was more favorable for the κ^1^-bound geometry of pathway 2 (^3^[**6a**]^2+^) to return to the ^3^MC-2a-6a state (activation barrier: 20.2 kJ/mol) than to break the second bpy nitrogen bond to go to ^3^MC-6a-11a (activation barrier: 28.1 kJ/mol). A lower bpy:dmphen dissociation ratio would, according to this analysis, be expected, which was indeed observed experimentally (1:3).

In acetonitrile like in water (Table 3), the most favorable pathway was pathway 1 for [**1a**]^2+^, with a total activation energy of 52.2 kJ/mol. As explained in the previous section, complex [**2a**]^2+^ had, unlike in water, a significantly lower barrier for pathway 1 (38.3 kJ/mol) than for pathways 2 and 3. In pathway 2 for complex [**1a**]^2+^ and [**2a**]^2+^, the occupation of the e_g_* orbitals led to a ^3^MC_trans_ state in which also the Ru-N_1_ was elongated. The flexibility of the dmbpy ligand allowed it to rotate around its C_2_-C_2'_ axis and form a stabilizing π-π interaction with one of the coordinated bpy ligand. The energy of activation in pathway 2 for [**1a**]^2+^ was, therefore, 10.3 kJ/mol higher (90.5 kJ/mol) than that of [**2a**]^2+^ (80.2 kJ/mol). This stabilization was less predominant in pathway 3, because in ^3^MC-2a-15a, the acetonitrile did not make a π-π interaction with one of the bpy ligands, as was observed in ^3^MC-1a-14a. The stabilization was still strong in comparison to pathway 1, resulting in a large activation barrier (62.7 and 67.8 kJ/mol for [**1a**]^2+^ and [**2a**]^2+^, respectively). According to these DFT calculations, in acetonitrile photodissociation of the dmbpy or dmphen ligand is preferable over dissociation of a bpy ligand. Indeed, for [**1a**]^2+^ we saw mainly the sterically hindering ligand dissociating in this solvent (no trace of free bpy), but for [**2a**]^2+^ there was a discrepancy: we observed a bpy:dmphen ratio of 6:1. We will come back to this point in the general discussion below.

**Table 3:** Thermal Activation Energy on the Triplet Hypersurface According to DFT Calculations for Reaction Pathways 1–3 in Acetonitrile^a^

complex	pathway	ligand dissociating	state (*x*)	state (*y*)	state (*z*)	Δ*E*_1_ (*x* – [a]) (kJ/mol)	Δ*E*_2_ (*z* – *y*) (kJ/mol)	Δ*E*_3_ (*x* – *y*) (kJ/mol)
[**1a**]^2+^	1	dmbpy	TS-1a-12a	[**12a]**^2+^		52.2		
	2	bpy	TS-1a-14a	[**14a**]^2+^	TS-14a-19a	90.5	10.8	20.7
	3	bpy	TS-1a-16a	[**16a]**^2+^		62.7		
								
[**2a**]^2+^	1	dmphen	TS-2a-13a	[**13a]**^2+^		38.3		
	2	bpy	TS-2a-15a	[**15a**]^2+^	TS-15a-20a	80.2	15.0	27.9
	3	bpy	TS-2a-17a	[**17a**]^2+^		67.8		

a [a] Energetically, the lowest on the triplet state.

### Bond Formation of the Second Solvent Molecule

To examine if the selectivity of the reaction was determined in the bond formation of the first solvent molecule, we also compared an interchange and dissociative-associative mechanism for the bond formation of the second solvent molecule. In contrast to the bond formation of the first solvent molecule, it was not possible to start from an initial geometry where the solvent molecule was already attached to ruthenium (e.g., from ^1^[**9a**]^2+^ or ^1^[**11a**]^2+^ for pathway 1 or 2 for [**1a**]^2+^ in water, see Scheme S4). To simulate the interchange mechanism, we added the second solvent molecule at a sufficient distance and intuitive angle. The interchange mechanism would react with the stable intermediates (^3^[**5a**]^2+^, ^3^[**6a**]^2+^, ^3^[**7a**]^2+^, ^3^[**8a**]^2+^, ^3^[**14a**]^2+^, and ^3^[**15a**]^2+^), where simultaneously, the second ruthenium nitrogen bond would break while the new ruthenium solvent bond would be formed. A dissociative-associative mechanism would occur by thermal activation to a ^3^MC state where the second ruthenium nitrogen bond would already be broken (^3^[**3a**]^2+^, ^3^[**4a**]^2+^, ^3^MC-5a-10a, ^3^MC-6a-11a, ^3^MC-7a-10a, ^3^MC-8a-11a, ^3^[**12a**]^2+^, ^3^[**13a**]^2+^, ^3^MC-14a-19a, ^3^MC-15a-20a, ^3^[**16a**]^2+^, and ^3^[**17a**]^2+^), after which the second solvent molecule would associatively bind to ruthenium. No possible pathways to the product were found for the interchange mechanism; hence, the dissociative-associative mechanism was found more favorable. Once the compound reaches the ^3^MC state where the second ruthenium nitrogen bond is broken, the association of the second solvent molecule is barrierless. Since the energy barrier to reach this ^3^MC state was always lower than the first energy barrier for the formation of the bond with the first solvent molecule, we concluded that the selectivity was not determined during the bond formation of the second solvent molecule.

### Activation Enthalpy of the Photosubstitution Reactions

**Figure 7:**
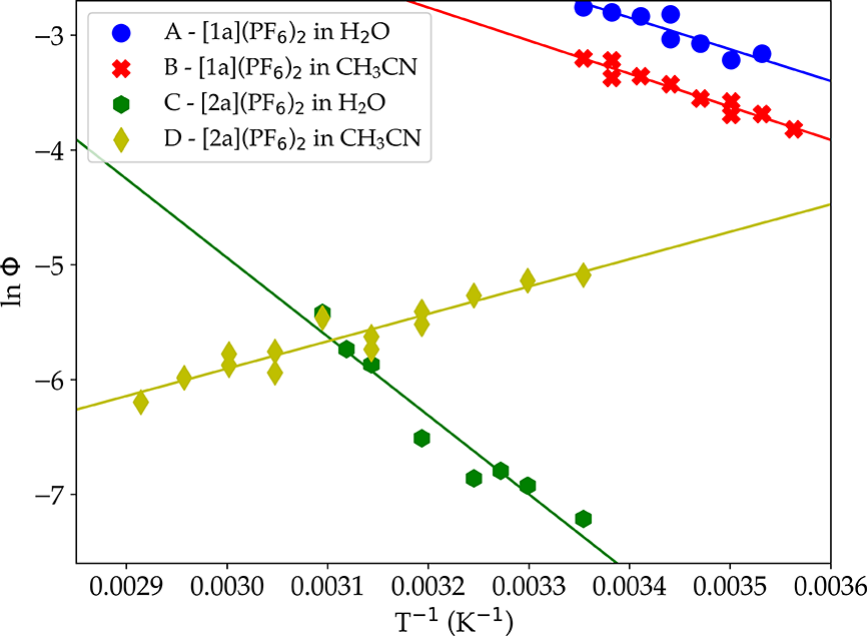
Eyring-type plot showing the evolution of the natural logarithm of the photosubstitution quantum yield vs 1/*T* for [**1a**](PF_6_)_2_ and [**2a**](PF_6_)_2_ in either a H_2_O:acetone 1:1 solution or acetonitrile. Conditions: (A, C) light power = 6.0 mW, λ_ex_ = 466 nm, [Ru] = 0.19 μM; (B) light power = 3.0 mW, λ_ex_ = 413 nm, [Ru] = 0.80 μM; (D) light power = 5.8 mW, = 413 nm, [Ru] = 0.80 μM.

In principle, our full model of the photosubstitution reaction provided a unique opportunity to compare these calculations to the experimental barrier of activation for the photosubstitution reaction of [**1a**](PF_6_)_2_ and [**2a**](PF_6_)_2_ in either water:acetone 1:1 or acetonitrile. To do so, the variation of the photosubstitution quantum yields Φ was determined as a function of temperature (see Table S4–S7). If we assume that the first-order rate constant k_nr_ for nonradiative decay follows the Eyring-Polanyi equation,^60,61^ we can express the evolution of ln(Φ) vs 1/T by eq 7a (see the experimental part for derivation):
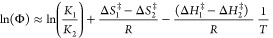
7awhere Δ*H*_1_^‡^ and Δ*S*_1_^‡^ are the enthalpy and entropy of activation, respectively, for the photosubstitution pathway, Δ*H*_2_^‡^ and Δ*S*_2_^‡^ are the enthalpy and entropy of activation, respectively, for nonradiative decay, and *K*_1_ and *K*_2_ are transmission coefficients. Linear regressions for ln(Φ) vs 1/*T*, where Φ is the experimental photosubstitution quantum yields at temperature *T*, are shown in Figure 7. As a note, the measured quantum yields for [2a]^2+^ are the total quantum yields for the photosubstitution of both ligands, as no difference could be made by UV–vis spectroscopy between the kinetics of the two reactions. The experimental difference in enthalpy of activation for each decay pathway, ΔΔ*H*_exp_^‡^ = Δ*H*_1, exp_^‡^ – Δ*H*_2, exp_^‡^, could be obtained experimentally from the slope of each linear regression. The resulting values of ΔΔ*H*_exp_^‡^ are indicated for both solvents and both complexes [**1a**]^2+^ and [**2a**]^2+^ in Table 4. In water:acetone 1:1, a positive value ΔΔ*H*_exp_^‡^ was obtained for both complexes [**1a**]^2+^ and [**2a**]^2+^, which demonstrated that the enthalpy of activation for photosubstitution (Δ*H*_1, exp_^‡^) was greater than that for nonradiative decay (Δ*H*_2, exp_^‡^). Since no change in volume occurred in these reactions, we assumed that the variation of internal energy Δ*E* calculated by DFT (Tables 2 and 3) were good approximations for the computed enthalpy of activations Δ*H*_1, comp_^‡^. As explained in the experimental part (eq 11), assuming the experimental Δ*H*_1, exp_^‡^ values and computed barriers Δ*H*_1, comp_^‡^ to be identical, we may interpret the differences between Δ*H*_1, comp_^‡^ and ΔΔ*H*_exp_^‡^ as an approximation for Δ*H*_2_^‡^, the barrier of activation for nonradiative decay.

**Table 4:** Experimental Difference in Enthalpy ΔΔ*H*_exp_^‡^, Theoretical Activation Enthalpy for Photosubstitution Δ*H*_1, comp_^‡^, and Activation Enthalpy for Nonradiative Decay Δ*H*_2, exp_^‡^^a^

solvent	complex	ΔΔ*H*_exp_^‡^ (kJ/mol)	Δ*H*_1, comp_^‡^ (kJ/mol)	Δ*H*_2, exp_^‡^ (kJ/mol)
water^b^	[**1a**](PF_6_)_2_	22.9 ± 0.03	37.5	14.6
	[**2a**](PF_6_)_2_	57.1 ± 0.03	64.7	7.6
acetonitrile	[**1a**](PF_6_)_2_	23.9 ± 0.02	52.2	28.3
	[**2a**](PF_6_)_2_	–20.6 ± 0.19	38.3	58.9

a Calculated according to eq 12

b Water:acetone 1:1 for experimental values.

The experimentally obtained values of ΔΔ*H*_exp_^‡^ for compound [**1a**](PF_6_)_2_ in water:acetone and acetonitrile were found positive and reasonably close to each other, i.e., 22.9 and 23.9 kJ/mol, respectively. Positive values were expected, as photosubstitution reactions become notoriously faster when temperature increases.^40,41^ The computed values Δ*H*_1, comp_^‡^ for photosubstitution by water and acetonitrile, on the other hand, differed significantly (37.5 vs 52.2 kJ/mol, respectively), which predicts a twice higher barrier for nonradiative decay in acetonitrile (28.3 kJ/mol), compared to water (14.6 kJ/mol). For [**2a**](PF_6_)_2_ in water, ΔΔ*H*_exp_^‡^ was also positive and significantly larger (57.1 kJ/mol) than for [**1a**](PF_6_), which meant that the enthalpy of activation for nonradiative decay was much lower than that of photosubstitution, 7.6 kJ/mol in comparison to 64.7 kJ/mol.

For compound [**2a**](PF_6_)_2_ in acetonitrile, however, a surprising result was observed: photosubstitution became slower when the temperature increased, i.e., the activation barrier difference ΔΔ*H*_exp_^‡^ was negative (−20.6 kJ/mol). To our knowledge, this observation has never been reported before. According to our interpretation and eq 7, this result does not mean that photosubstitution is not thermally activated, but that for this complex in this solvent, the barrier for nonradiative decay (Δ*H*_2, exp_^‡^ = 58.9 kJ/mol) is higher than the activation barrier for photosubstitution (Δ*H*_1_^‡^ = 38.3 kJ/mol). This result was clear-cut but rather counter-intuitive: its most direct consequence would be that there must be (low) temperatures where the photosubstitution quantum yield should become very high, while at ambient temperatures, Φ_413_ was still rather low (0.0050).

### Computed Potential Energy Surface

**Figure 8:**
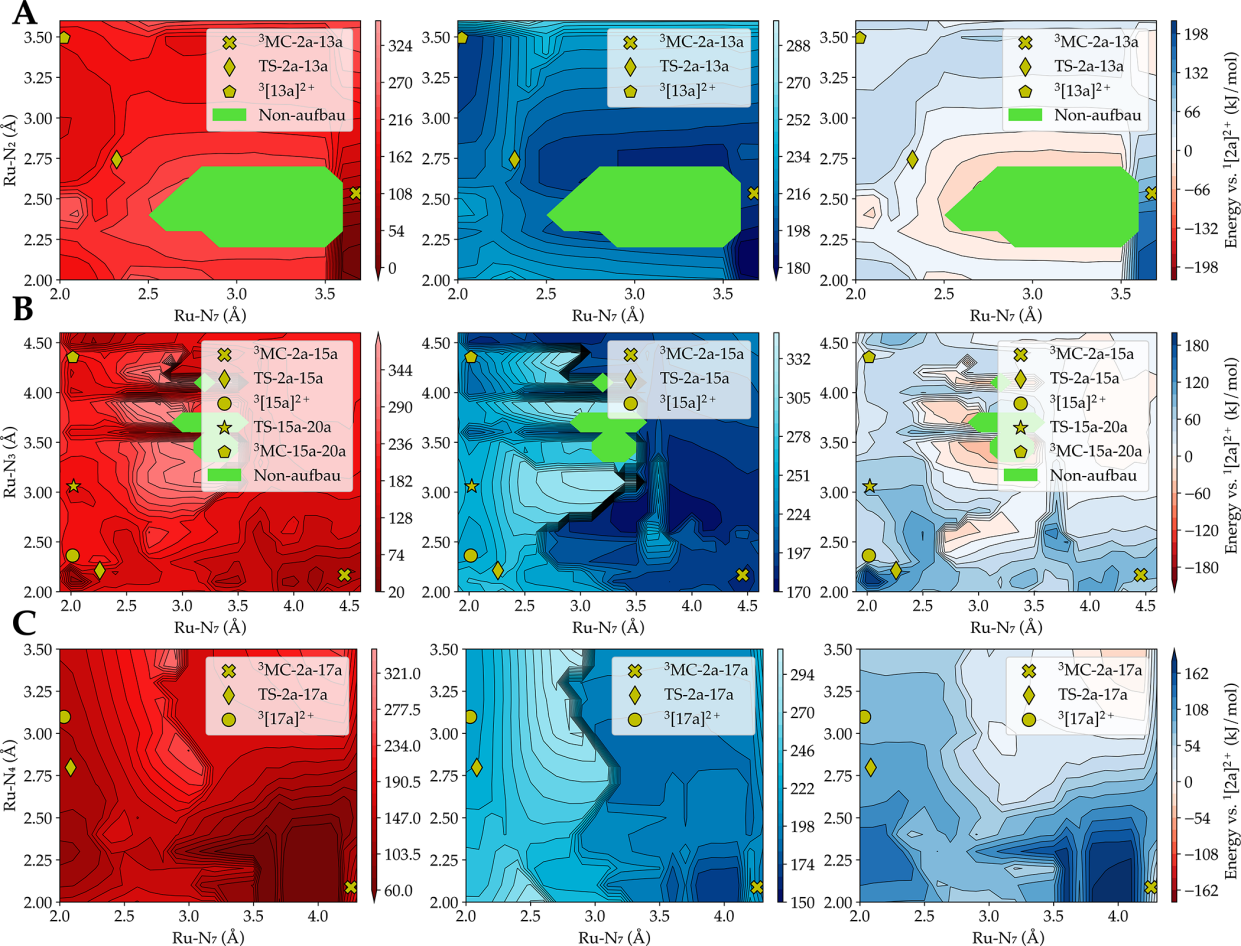
Mapping of the singlet ground-state energy, of the lowest triplet state energy, and of the energy difference between these two surfaces, for pathway 1 (A), 2 (B), and 3 (C) of complex [**2a**]^2+^ in acetonitrile. The ^3^MC states and transition states for the first step of the photosubstitution are also shown as yellow symbols. In pathway 1 (A), the reaction needs to pass through a vast area in which the singlet surface is higher in energy than the triplet surface. Therefore, the probability of deactivation by intersystem crossing from the reactive triplet hypersurface to the singlet hypersurface is considerably larger. Level of theory: PBE/TZP/D3-BJDAMP/ ZORA /COSMO.

To understand the unusual negative barrier of activation ΔΔ*H*_exp_^‡^ observed for [**2a**]^2+^ in acetonitrile, the singlet and triplet hypersurfaces for both complexes [**1a**]^2+^ and [**2a**]^2+^ were generated in both solvents to investigate whether there was a possibility of nonradiative decay through intersystem crossing during photosubstitution. The triplet surface was first generated by a potential energy surface scan. Two reaction coordinates, the same as those elongated in Figure 5, were used to perform this computation; for each point, the geometry was optimized while constraining the two reaction coordinates. Subsequently, the energy of the same geometry on the singlet hypersurface was calculated. Figure 8 shows the singlet ground-state energy (left), the lowest triplet state energy (center), and the difference between these two hypersurfaces (right) color-coded for [**2a**]^2+^ in acetonitrile. In the difference graph, red areas indicate a region where the singlet hypersurface is higher in energy than the triplet surface, blue areas a region where the triplet surface is higher in energy, and white areas regions in which the triplet and singlet surfaces do not differ in energy very much. In other words, the white areas represent regions along the potential energy surface where the probability of intersystem crossing and nonradiative decay to the ground state is high. The areas coded in green corresponded to quasi-degenerate regions, as a non-Aufbau solution was found here. In these green regions, the energies can, therefore, not be trusted, as the states are of multiconfigurational character, which DFT cannot accurately describe.

According to these energy maps, only the transition state of pathway 1 of [**2a**]^2+^ (TS-2a-13a, Figure 8A) was surrounded by a broad white region of small energetic difference between the singlet and triplet state. For pathways 2 and 3 (Figure 8B,C, respectively), in which bpy ligand dissociation occurs, the transition state TS-2a-15a and TS-2a-17a, respectively, were located in a blue area where the triplet hypersurface is clearly above the singlet hypersurface. As pathway 1 has the lowest computed barrier for [**2a**]^2+^ in acetonitrile, when undergoing photosubstitution, the molecule will, at low temperatures, pass along the minimum energy path where the chances to go back to the ground state are considerable. As the temperature increases, deviations from the minimum energy path will increase the chances of nonradiative decay to the ground state, as points of degeneracy at higher energies are visited. This phenomenon is also responsible, according to our understanding of the system, for the lower photosubstitution rate of the dmphen ligand, compared to bpy, when [**2a**]^2+^ is irradiated in acetonitrile.

## Discussion

Our results show that the rigidity of the ligand significantly affects the selectivity and efficiency of the photosubstitution reaction of ruthenium polypyridyl complexes. [**1a**]^2+^, [**1b**]^2+^, and [**2b**]^2+^ all have nonmethylated ligands that are as rigid or less rigid than the methylated ligands. For these compounds, a selective photosubstitution of the straining ligand was observed in both solvents water and acetonitrile. However, if the straining methylated ligand was more rigid than the nonmethylated ligands, as in [**2a**]^2+^, then unselective photosubstitution occurred.

In aqueous solution, an incoming water molecule forms a hydrogen bond with the dissociating straining ligand, after which a proton transfer occurs in the transition state. The inability of the rigid dmphen ligand of [**2a**]^2+^ to rotate around its central C_11_-C_12_ axis results in a more strained complex during proton transfer in TS-2a-4a, TS-2a-6a, and TS-2a-8a, resulting in a higher energy barrier in comparison to the nonrigid dmbpy ligand in [**1a**]^2+^. The energy barriers of pathway 1 and 2 are approximately equal in height, which would suggest that a 1:1 ratio between free bpy and free dmphen should be observed. However, whereas dissociation of the dmphen ligand requires a single energy barrier to be crossed, dissociation of the bpy ligand is a two-step process in which a stable intermediate is formed. After forming this intermediate geometry, it is more favorable, (by ∼8.0 kJ/mol) for the complex to return to the ^3^MC state and finally to the ^3^MLCT state, than to go to the final product. Therefore, not every crossing will lead to the photoproduct, and in accordance with experiments, it is more likely that substitution of the dmphen ligand occurs.

At first sight, the photodissociation reaction in acetonitrile showed some similarities compared with that in water-containing solutions. The computed energy barriers for [**1a**]^2+^ also suggested that only dissociation of the straining ligand would occur, which was experimentally observed in both water:acetone and acetonitrile. Even though the dissociation of the nonrigid dmbpy ligand in water:acetone required less thermal energy (37.5 kJ/mol) than in acetonitrile (52.2 kJ/mol), the relative difference between the enthalpy of activation for photosubstitution (Δ*H*_1, exp_^‡^) and that for nonradiative decay (Δ*H*_2, exp_^‡^) was similar for both solvents (ΔΔ*H*_exp_^‡^ = 22.9 kJ/mol in H_2_O vs 23.9 kJ/mol in acetonitrile). A clear discrepancy was found for the photodissociation of the rigid dmphen ligand of [**2a**]^2+^ in acetonitrile. In particular, in this solvent, the calculated energy barrier for dmphen photodissociation was particularly low (38.3 kJ/mol), i.e., lower than that for the nonrigid bpy ligands (70–80 kJ/mol), but also lower than that for the substitution of the nonrigid dmbpy ligand in [**1a**]^2+^ in acetonitrile.

We interpret this inconsistency as the result of three different phenomena. First, the inability of acetonitrile to form a hydrogen bond with the leaving κ^1^-bound bidentate ligand; second, the rigidity of the dmphen ligand; and third, the size of its conjugated π system. The rigid dmphen ligand cannot rotate around its C_11_-C_12_ axis and is not kept in place by a hydrogen bond as it is in water. This allows dmphen to relocate in TS-2a-13a to a position in which it can form a stronger, highly stabilizing π-π interaction with one of the bpy ligands. By contrast, the nonrigid dmbpy ligand in [**1a**]^2+^ rotates around its C_2_-C_2'_ axis, which allows the formation of a π-π interaction with the incoming acetonitrile ligand. This interaction prohibits dmbpy from relocating to a position in which it could form a stronger π-π interaction with one of the bpy ligands, thereby increasing the energy barrier in TS-1a-12a compared to TS-2a-13a. The energy barriers of all three pathways in acetonitrile suggested that both complexes would only expulse the straining ligand. Experimentally, this prediction was confirmed for [**1a**]^2+^, but a nonselective photosubstitution reaction of 6:1 in favor of bpy was observed for [**2a**]^2+^. It seemed that the low barrier for dmphen substitution was outcompeted by another faster pathway.

This hypothesis was confirmed by the very unusual experimental observation that dmphen photodissociation in [**2a**](PF_6_)_2_ in acetonitrile decreased in rate when the temperature was increased. Upon mapping the energy difference between the singlet and triplet energy surfaces, it appeared that regions in all three reaction pathways exist where the energy differences between the singlet and triplet surfaces were almost zero (white regions in Figure 8). For such regions, there is a high probability of intersystem crossing from the triplet hypersurface back to the singlet ground state, which corresponds to nonradiative decay. Qualitatively, these regions are distributed differently for both compounds in both solvents: they can be either close or far from the transition states that the system needs to cross to go from one intermediate along the photosubstitution pathway to the next one (see Figures S28–S31). When the white regions are close to the TS, lower photosubstitution quantum yields are expected, as going through the TS increases the probability of nonradiative decay to the singlet ground state. For [**2a**]^2+^ in acetonitrile, dissociation of the dmphen ligand (pathway 1) goes via the TS-2a-13a state that is right in the middle of a vast white region (Figure 8A). Upon increasing the temperature, more trajectories will go through these white areas around the TS-2a-13a, increasing the probability of nonradiative decay and decreasing the photosubstitution quantum yield. If the temperature was lower, most trajectories would go through TS-2a-13a, where there is still a probability of ISC, which is why we observe a low quantum yield at ambient temperature (Φ_413_ = 0.0050). The transition state TS-2a-13a along pathway 1 is lower in energy in comparison to TS-2a-15a along pathway 2 and TS-2a-17a along pathway 3. However, as can be seen in Figure 8B,C, there are virtually no areas around the TS-2a-15a and TS-2a-17a where the singlet and triplet hypersurface are quasi-degenerate. Following one of these two pathways will lead to a higher chance of dissociating the bpy ligand, compared to pathway 1, as the area along these routes where intersystem crossing is likely, is small. The dissociation probability will, therefore, be higher for the bpy ligand than for the dmphen ligand. In other words, it is the topology of the singlet and triplet hypersurfaces along the three photosubstitution pathways for [**2a**]^2+^ in acetonitrile, which explains the observed 6:1 ratio between free bpy and free dmphen observed experimentally at the end of the photosubstitution.

Although most experimental results can be interpreted using our computational workflow, there is still some room for improvement in interpreting Δ*H*_2, exp_^‡^. At first sight, we speculated that it would represent the enthalpic energy necessary to attain an energy level where the probability of intersystem crossing would become significant. However, according to the transition state theory, we would anticipate *k*_nr_ to increase with temperature faster than *k*_ps_ if Δ*H*_2_^‡^ was smaller than Δ*H*_1_^‡^, and its increase would be slower if Δ*H*_2_^‡^ was larger than Δ*H*_1_^‡^. Our findings contradict this assumption, as photosubstitution became faster when the temperature increased while Δ*H*_2_^‡^ < Δ*H*_1_^‡^ for [**1a**]^2+^ in water:acetone and acetonitrile, as well as for [**2a**]^2+^ in water:acetone. By using the Eyring equation to model the temperature dependence of the rate of nonradiative decay, we over-simplify the system. The process of nonradiative decay involves intersystem crossing from a triplet to a singlet hypersurface, followed by vibrational relaxation to the ground vibrational state of the electronic ground state. Modeling the temperature variation of this process, to compare to experimental values, is challenging because one should integrate in the analysis (i) the energy variation of both hypersurfaces with geometry, (ii) the overlap between the vibrational wavefunction in the singlet and triplet electronic states, and (iii) the spin-forbidden nature of the transition.^62^ Additionally, each compound has specific topologies for its triplet and singlet hypersurfaces, which are strongly dependent on the solvent, causing significant variations in the probability of intersystem crossing. Therefore, although our data can be fitted using Eyring’s equation, it remains speculative whether this treatment is a fully accurate description of the temperature variation of the rate constant of T_1_ → S_0_ intersystem crossing.

## Conclusions

In this study, we have examined the influence of the solvent, temperature, and ligand rigidity, on the selectivity and quantum efficiency of photosubstitution reactions in sterically hindered ruthenium complexes. Selective photosubstitution of the straining ligand occurred when the ligand was as rigid as, or less rigid than, the nonstraining ligands. By contrast, unselective photosubstitution was observed for [**2a**]^2+^, in which the straining ligand was more rigid. For this compound, the selectivity of the photosubstitution reaction was highly dependent on the nature of the incoming ligand, and hence of the solvent. The dmphen was photosubstituted preferentially in acetone:water mixtures, while in MeCN, the bpy ligand was cleaved faster. The quantum efficiency of the photoreaction was regulated mainly by the rigidity of the photosubstituted diamine ligand, with faster photosubstitutions for bipyridines than for phenanthrolines. When measuring the activation barriers of photosubstitution for [**1a**]^2+^ and [**2a**]^2+^, a normal behavior was found in water, where increasing temperatures increased the photosubstitution quantum efficiency. [**2a**]^2+^ showed the same behavior in water, but it showed an “abnormal” behavior in acetonitrile, where photosubstitution quantum yields decreased when the temperature increased. For the first time, the experimental energy barriers for photosubstitution and selectivities could be rationalized by a full DFT modeling of the singlet and triplet hypersurfaces of the complexes that included explicit solvent molecules. In water, a proton transfer was observed between the incoming water molecule and ^3^MLCT-like triplet intermediates; the calculated energy barriers compared well to the experimentally measured activation enthalpies of the photoreaction. In acetonitrile, however, no proton transfer could occur, and a discrepancy was found between experimental and calculated photosubstitution activation enthalpies. This discrepancy was explained by the higher probability of nonradiative decay back to the ground state when the triplet transition states were located in regions where the singlet and triplet hypersurfaces were almost degenerate. These results highlight the active role of the solvent in the fine-tuning of the reactivity of photosubstitutionally active metal complexes. In fact, solvent molecules need to be included in theoretical models to rationalize the mechanisms, rates, activation barriers, and selectivity, obtained experimentally. Overall, future research should strive to bridge the gap between experimental and computational work, to facilitate the understanding of solvent effects in photosubstitution reactions.

## Method Section

### Experimental Section

#### Synthesis and Characterization

Compounds [**1a**](PF_6_)_2_, [**1b**](PF_6_)_2_, [**2a**](PF_6_)_2_, and [**2b**](PF_6_)_2_ were synthesized according to modified literature procedures (see the Supporting information).

#### Photosubstitution Studies Monitored by UV–vis Absorption Spectroscopy and MS

UV–vis experiments on the ruthenium complexes were performed on a Cary 50 Varian spectrometer equipped with a Cary Single Cell Peltier for temperature control (*T* = 298 K) and stirring. Experiments were performed in 1.0 × 1.0 cm fluorescence cuvettes (QS-111, Hellma Analytics) containing 3.00 mL of solution. A stock solution of the desired complex was prepared in either acetonitrile or acetone, which was then diluted to the desired working concentration and placed in the cuvette. Irradiations were carried out under an N_2_ atmosphere after deoxygenation for 10 min by gentle bubbling of N_2_ through the sample, and the sample was kept under an inert atmosphere during the experiment by a gentle flow of N_2_ over the top of the cuvette. Irradiation was performed from the top of the cuvette using a custom-build LED irradiation setup, consisting of a high-power LED (λ = 413 nm, FWHM = 17 nm or λ = 466 nm, FWHM = 36 nm, part no. H2A1-H410 and H2A1H470, Roithner Lasertechnik), driven by a LED driver operating at 50–350 mA. Irradiance photon fluxes were determined using potassium ferrioxalate actinometry (see Table S3). UV–vis absorption spectra were recorded every 6 s. Mass spectrometry (see general section above for details) was performed after the irradiation experiments to identify the photoproducts. Data were analyzed using Microsoft Excel 2010. The rate constants of the photosubstitution reactions (*k*_Φ_) were derived by fitting the time evolution of the UV–vis absorption at 450 nm to a monoexponential decay function using Origin Pro 9.1. As the irradiation wavelength was chosen close to the isosbestic point in the photosubstitution reactions, we assumed that *A*_λ_ (λ = 413 or 466 nm) was constant in time, so that the obtained rate constants could be converted into quantum yields for the photosubstitution reactions (Φ_413_ and Φ_466_) using eq 1.^63^
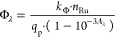
1where *k*_Φ_ is the observed first-order rate constant for photosubstitution (in s^–1^), *n*_Ru_ is the total amount of ruthenium ions (in mol), *q*_p_ is the incoming photon flux (in mol s^–1^), and *A*_λ_ is the absorbance of the solution at the irradiation wavelength.

#### Photosubstitution Studies Monitored by ^1^H NMR Spectroscopy

An aliquot of either compound [**1a**](PF_6_)_2_, [**2a**](PF_6_)_2_, [**1b**](PF_6_)_2_, or [**2b**](PF_6_)_2_ (1 mg) was placed into an NMR tube, and under a nitrogen atmosphere. It was then dissolved under an N_2_ atmosphere in 0.65 mL of deoxygenated deuterated solvent (either CD_3_CN or a mixture of acetone-*d*_6_ and D_2_O). The tube was closed and sealed with PTFE tape. It was then irradiated at room temperature with a 850 W LOT Xenon arc lamp equipped with both an IR shortpass and 400 nm longpass filter. In addition, a second NMR tube was kept in the dark for the duration of the experiment as a control sample. ^1^H-NMR spectra were taken at various time intervals to monitor the photoreaction. Data were analyzed using MestReNova 11.0.

#### Singlet Oxygen Generation and Phosphorescence Quantum Yield of [**1a**–**2b**](PF_6_)_2_

See the Supporting information.

#### Activation Enthalpy of Photosubstitution Reaction

Stock solutions of complex [**1a**](PF_6_)_2_ and [**2a**](PF_6_)_2_ were prepared in a H_2_O:acetone 1:1 mixture and in acetonitrile (see Tables S4–S7). A 1.0 × 1.0 cm quartz cuvette was used for the UV–vis measurements. The stock solution (3 mL) was put in the cuvette and deoxygenated for 10 min by gently bubbling N_2_ gas through the cuvette. The sample was kept under an inert atmosphere during the experiment by a gentle flow of N_2_ over the solvent air interface. The temperature was controlled by a Peltier system. A UV–vis absorption spectrum was measured every 6 s horizontally, while the sample was irradiated with blue light vertically (413 nm in H_2_O:acetone; 466 nm in ACN).

The photosubstitution reaction was repeated at different temperatures, and for each temperature, the reaction rate constants (*k*_Φ_) were derived by fitting the time evolution of the UV–vis absorption spectrum with Glotaran.^64^ The irradiation wavelength was chosen close to the isosbestic point so that it could be assumed that the absorbance of the solution at irradiation wavelength *A*_λ_ was constant in time. The rate constants can be converted in the photosubstitution quantum yield (Φ) following eq 1. After excitation of the complex, two main competing reaction pathways influence the quantum yield: photosubstitution (rate constant k_ps_) vs nonradiative decay (rate constant *k*_nr_). Φ is given by eq 2:
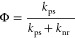
2

The rate constants of both pathways can be written in their Eyring-Polanyi equation form following the activated complex theory:^65,66^
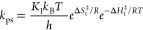
3
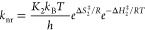
4

For each pathway, *K* represents the transmission coefficient through the activated state (<1), *k*_B_ (in m^2^ kg s^–2^ K^–1^) is Boltzmann’s constant, *T* (in K) is the temperature, *h* (in m^2^ kg s^–1^) is Planck’s constant, Δ*S*^‡^ (J mol^–1^ K^–1^) is the entropy of activation of the reaction, *R* (kg m^2^ K^–1^ mol^–1^ s^–2^) is the perfect gas constant, and Δ*H*^‡^ (J mol^–1^) is the enthalpy of activation of the deactivation pathway (photosubstitution or nonradiative decay). As initial calculations indicated that the rate constant of the nonradiative decay pathway was substantially larger than that of the photosubstitution pathway (Φ ≪ 1, experimentally typically a few percent), we reasoned that the quantum yield can be approximated by eq 5, which can rearrange into eq 6:
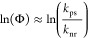
5
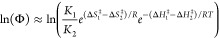
6
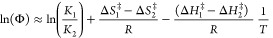
7

From eq 7, we deduced that, by plotting the logarithm of the photosubstitution quantum yield Φ versus 1/*T*, we obtain a slope that, after multiplication by -R, is equal to the difference between the enthalpy of activation of the photosubstitution and nonradiative decay pathways:
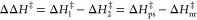
8

A comparison can be made between our experimental and computational values, since there is no variation in the change of volume. By rewriting eq 8, we can deduce, while taking computational error into consideration, that the difference between Δ*H*_1, comp_^‡^ and ΔΔ*H*_exp_^‡^ gives an approximation to Δ*H*_2, exp_^‡^.
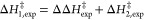
9
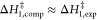
10
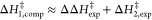
11
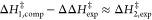
12

### Computational Methods

All DFT calculations were performed with the ADF 2019 package.^67^ We used PBE^68^ as exchange-correlation functional, and a TZP basis set was used for the ruthenium atom, while a DZP basis set was chosen for the rest of the atoms.^69,70^ Relativistic effects were scalarly corrected for by ZORA,^71^ and dispersion effects were corrected by Grimme’s D3 correction with BJ damping.^72^ Implicit solvent effects were considered via the COSMO model either in water or acetonitrile.^73−75^ The ground-state geometries were optimized by restricted Kohn-Sham DFT, and the triplet states were determined with its unrestricted analogue in combination with the collinear approximation.^76,77^

Areas around the transition state were calculated via a potential energy surface scan. The potential energy scan was performed along two reaction coordinates. These coordinates were constrained in the triplet state, while the rest of the geometry was relaxed. The optimized geometry was used to perform single-point calculations of the singlet state to obtain the corresponding singlet surface, and ultimately, the difference between both surfaces was calculated.

## Supporting Information Available

Experimental details, photosubstitution studies, X-ray crystallographic data, and all computational data of the compounds, accompanied with supporting graphs for each pathway, can be found in the Supporting Information (PDF)
Raw data is available on Zenodo (https://doi.org/10.5281/zenodo.7845382)
