# Supplementary material for: Ligand Rigidity Steers the Selectivity and Efficiency of the Photosubstitution Reaction of Strained Ruthenium Polypyridyl Complexes
Source: J Am Chem Soc. 2023 Jun 9;145(24):13420–34. doi: 10.1021/jacs.3c03543 (PMC10299800; doi:10.1021/jacs.3c03543)
Supplement: Supplementary file 1 — ja3c03543_si_001.pdf [file ja3c03543_si_001.pdf]

Supporting Information

Ligand rigidity steers the selectivity and efficiency of the photosubstitution reaction of strained ruthenium polypyridyl complexes

Matthijs L. A. Hakkennes,[†] Michael S. Meijer,[†] Jan Paul Menzel,[†] Anne-Charlotte Goetz, Roy van Duijn, Maxime A. Siegler,[°] Francesco Buda,[†] Sylvestre Bonnet[*†]*

[†]Leiden Institute of Chemistry, Leiden University, P.O. Box 9502, 2300 RA Leiden, The Netherlands; °Department of Chemistry, Johns Hopkins University, 3400 N Charles Street, Baltimore, MD, 21218, United States.

*Corresponding author: *bonnet@chem.leidenuniv.nl* , *buda@chem.leidenuniv.nl*

EXPERIMENTAL SUPPORTING INFORMATION 3

General synthesis 3

Photosubstitution studies monitored by UV-Vis absorption spectroscopy and MS 14

Photosubstitution studies monitored by ^{1}H NMR spectroscopy 16

Singlet oxygen generation and phosphorescence quantum yield of [1a–2b](PF_6)$_2$ 18

Absolute phosphorescence quantum yield of [Ru(bpy)$_3$Cl$_2$] 19

COMPUTATIONAL SUPPORTING INFORMATION 26

Structures Singlet 26

Structures Triplet 29

Reaction Pathways of First Solvent Molecule 32
- **Complex [1a]$^{2+}$ - H_2O – Pathway 1** 32
- **Complex [2a]$^{2+}$ - H_2O – Pathway 1** 33
- **Complex [1a]$^{2+}$ - H_2O – Pathway 2** 34
- **Complex [2a]$^{2+}$ - H_2O – Pathway 2** 35
- **Complex [2a]$^{2+}$ - H_2O – Pathway 3** 37
- **Complex [1a]$^{2+}$ - CH_3CN – Pathway 1** 38
- **Complex [2a]$^{2+}$ - CH_3CN – Pathway 1** 39
- **Complex [2a]$^{2+}$ - CH_3CN – Pathway 2** 41
- **Complex [1a]$^{2+}$ - CH_3CN – Pathway 3** 42
- **Complex [2a]$^{2+}$ - CH_3CN – Pathway 3** 43

Singlet and Triplet Potential Energy Surfaces 44
- **Complex [1a]$^{2+}$ - H_2O** 44
- **Complex [2a]$^{2+}$ - H_2O** 45
- **Complex [1a]$^{2+}$ - CH_3CN** 46

REFERENCES 47

Experimental Supporting Information

General synthesis

Dry DMF was obtained by storing a freshly opened bottle of DMF over 3 Å molecular sieves under N_2 atmosphere. Dry acetonitrile was collected from a Pure Solve MD5 dry solvent dispenser (Demaco). All other reagents and solvents, were purchased from Sigma-Aldrich, and used as received. Ruthenium complex synthesis was conducted in oxygen-free atmosphere using standard Schlenk-line techniques in the absence of light. Flash column chromatography was performed on silica gel (Screening devices B.V.) with a particle size of 40–64 µm and a pore size of 60 Å. TLC analysis was conducted on TLC aluminium foils with silica gel matrix (Supelco, silica gel 60, art. nr. 56524) with detection by UV-absorption (254 nm). *cis*-[Ru(bpy)$_2$(CH$_3$CN)$_2$](CF$_3$SO$_3$)$_2$ was prepared following a literature method.[1]

All ^{1}H NMR spectra were recorded on a Bruker AV-300 or AV-400 spectrometer. Chemical shifts (δ) are indicated in ppm relative to TMS or the solvent peak. Mass spectra were recorded by using a MSQ Plus Spectrometer fitted with a Dionex automatic sample injection system. High resolution mass spectra were recorded by direct injection (2 µl of 1 µM solution in MeOH or acetonitrile and 0.1% formic acid) in a mass spectrometer (Thermo Finnigan LTQ Orbitrap) equipped with an electrospray (250 °C) with resolution R = 60,000 at m/z = 400 (mass range m/z = 150–2000) and dioctylphthalate (m/z = 391.28428) as a lock mass. The high-resolution mass spectrometer was calibrated prior to measurements with a calibration mixture (Thermo Finnigan).

[Ru(bpy)$_2$(dmbpy)](PF$_6$)$_2$, [1a](PF$_6$)$_2$: Compound [**1a**](PF$_6$)$_2$ was prepared by a modified literature procedure.[2] *cis*-[Ru(bpy)$_2$Cl$_2$] (100 mg, 0.206 mmol) and 6,6'-dimethyl-2,2'-bipyridine (32 mg, 0.172 mmol) were dissolved in ethylene glycol (4 mL) in a 25-mL Teflon-lined pressure vessel. The mixture was heated to 200 °C for 6 h. After cooling the reaction mixture to RT, it was poured onto H_2O (25 mL). Addition of saturated aq. KPF$_6$ (5 mL) resulted in a dark orange precipitate, which was collected using filtration, and washed with cold water and diethyl ether. Purification of the obtained solid was performed by silica column chromatography, using a mixture of acetone, water and sat. aq. KPF$_6$ solution (100:10:2) as the eluent. The desired fractions were combined and concentrated *in vacuo*. The resulting orange precipitate was collected by filtration, and washed with water and diethyl ether, yielding compound [**1a**](PF$_6$)$_2$ (87 mg, 0.098 mmol, 57%). ^{1}H NMR (400 MHz, δ in CD$_3$CN): 8.49 (d, J = 8.1 Hz, 2H), 8.40 (d, J = 8.1 Hz, 2H), 8.28 (d, J = 7.9 Hz, 2H), 8.09 (td, J = 7.9, 1.5 Hz, 2H), 7.97 – 7.89 (m, 6H), 7.62 (ddt, J = 26.3, 5.6, 1.4, 0.7 Hz, 2H), 7.46 (ddd, J = 7.6, 5.6, 1.3 Hz, 2H), 7.29 (d, J = 7.6 Hz, 2H), 7.21 (ddd, J = 7.4, 5.7, 1.4 Hz, 2H), 1.65 (s, 6H); ESI-MS (CH$_3$CN) m/z exp. (calc.): 299.5 (299.1, [M−2PF$_6$]$^{2+}$), 597.5 (597.1, [M−2PF$_6$−H]$^+$), 743.7 (743.1, [M−PF$_6$]$^+$). UV-Vis: λ_{max} in nm (ε in M^{-1}·cm^{-1}): 451 (9.87 × 10^3) in CH$_3$CN, 452 (1.25 × 10^4) in acetone/H$_2$O (1:1).

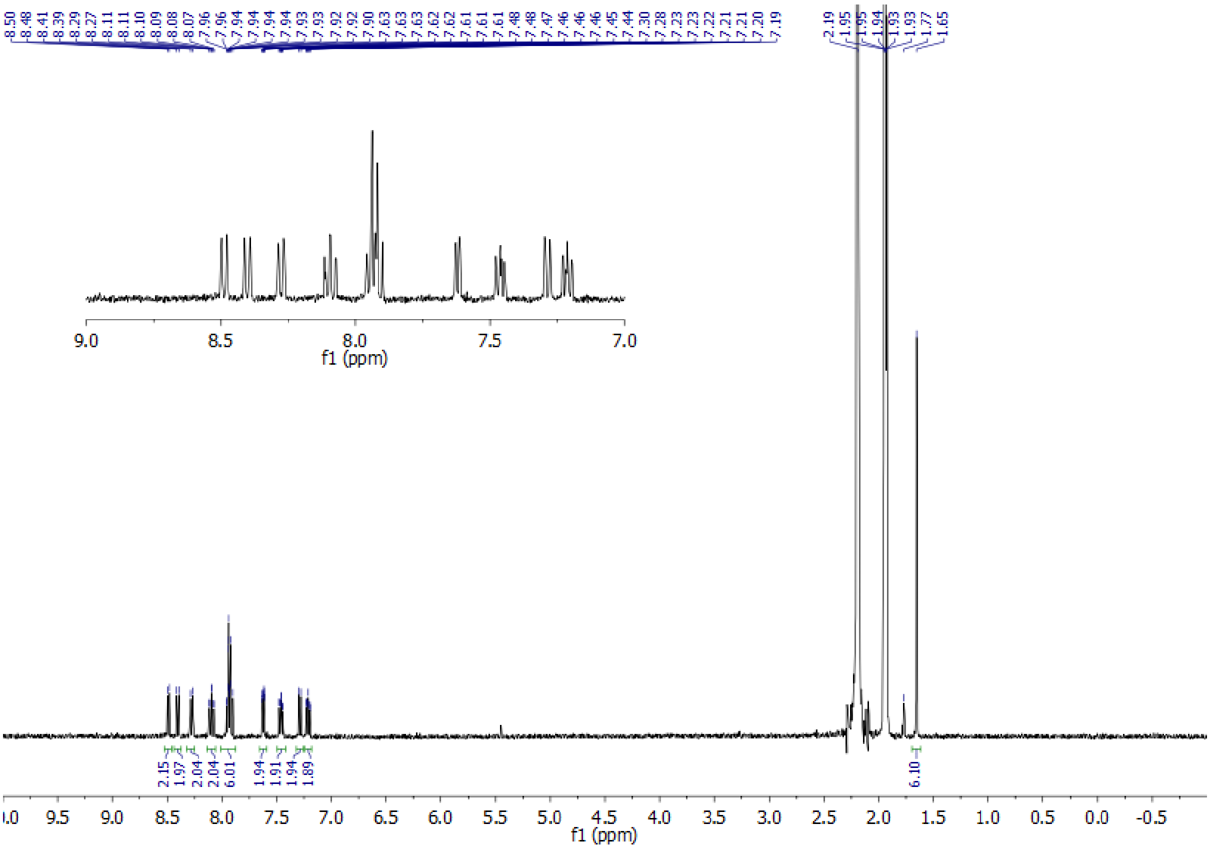

Figure S1. ^{1}H NMR spectrum of compound [1a]$(PF_6)_2$ in CD_3CN.

[Ru(bpy)$_2$(dmphen)]$(PF_6)_2$, [2a]$(PF_6)_2$: Compound [**2a**]$(PF_6)_2$ was prepared by the same procedure used for [**1a**]$(PF_6)_2$, using 2,9-dimethyl-1,10-phenanthroline (36 mg, 0.172 mmol). Yield: 80 mg (0.088 mmol, 51%). ^{1}H NMR spectroscopy and MS spectrometry match literature data.[3] UVVis: λ_{max} in nm (ε in $M^{-1}\cdot cm^{-1}$): 451 (1.39×10^4) in CH_3CN, 452 (1.31×10^4) in acetone/H_2O (1:1).

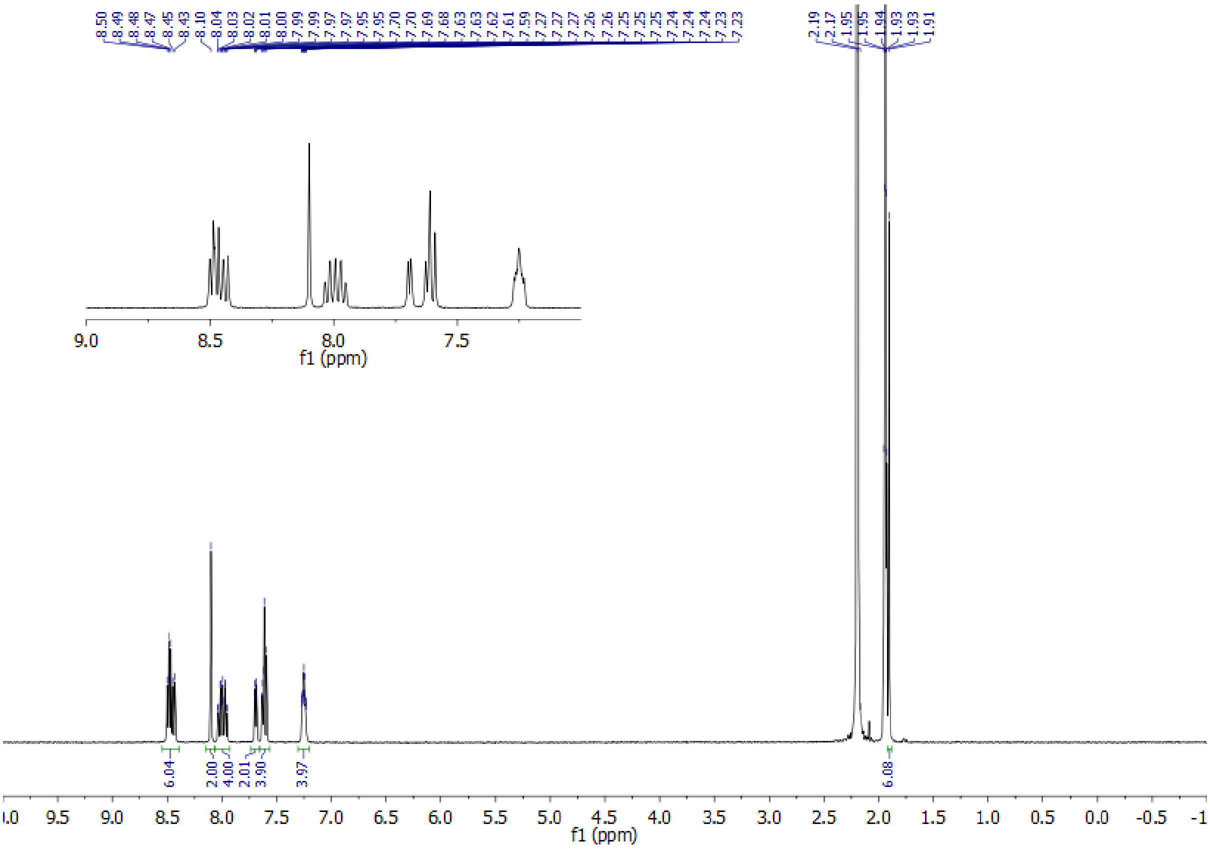

Figure S2. ^{1}**H NMR spectrum of compound [2a](PF_6)$_2$ in CD_3CN.**

***cis*-[Ru(phen)$_2$Cl$_2$], [3]:** *cis*-[Ru(phen)$_2$Cl$_2$] was prepared by a literature method.[4] Yield: 609 mg (1.14 mmol, 75%). For information: ^{1}H NMR (400 MHz, δ in DMSO-*d6*): 10.28 (dd, J = 5.3, 1.3 Hz, 2H), 8.71 (d, J = 8.3, 1.4 Hz, 2H), 8.29 (d, J = 8.8 Hz, 2H), 8.24 (d, J = 7.0 Hz, 2H), 8.21 (dd, J = 7.2, 4.3 Hz, 2H), 8.14 (d, J = 8.9 Hz, 2H), 7.75 (dd, J = 5.5, 1.2 Hz, 2H), 7.33 (dd, J = 8.1, 5.4 Hz, 2H).

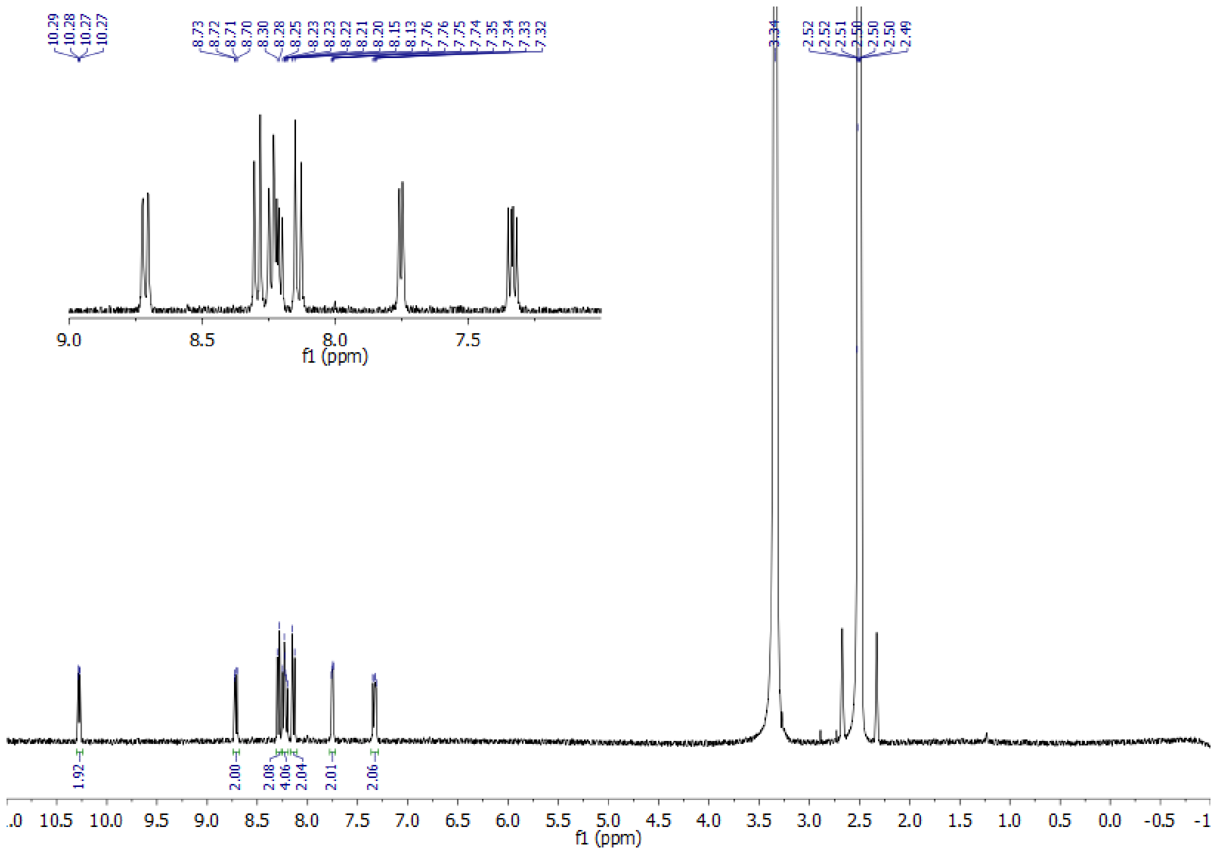

Figure S3. ^{1}H NMR spectrum of compound [3] in DMSO-*d6*.

[Ru(phen)$_2$(dmbpy)](PF$_6$)$_2$, [1b](PF$_6$)$_2$: Compound [**1b**](PF$_6$)$_2$ was prepared by the same procedure used for [**1a**](PF$_6$)$_2$, using *cis*-[Ru(phen)$_2$Cl$_2$] ([**3**], 100 mg, 0.188 mmol). Yield: 96 mg (0.103 mmol, 60%). ^{1}H NMR matches literature data.[5] ESI-MS (CH$_3$CN) m/z exp. (calc.): 323.0 (322.9, [M−2PF$_6$]$^{2+}$). UV-Vis: λ_{max} in nm (ε in M^{-1}·cm^{-1}): 449 (1.58 × 10^4) in CH$_3$CN, 450 (1.50 × 10^4) in acetone/H$_2$O (1:1).

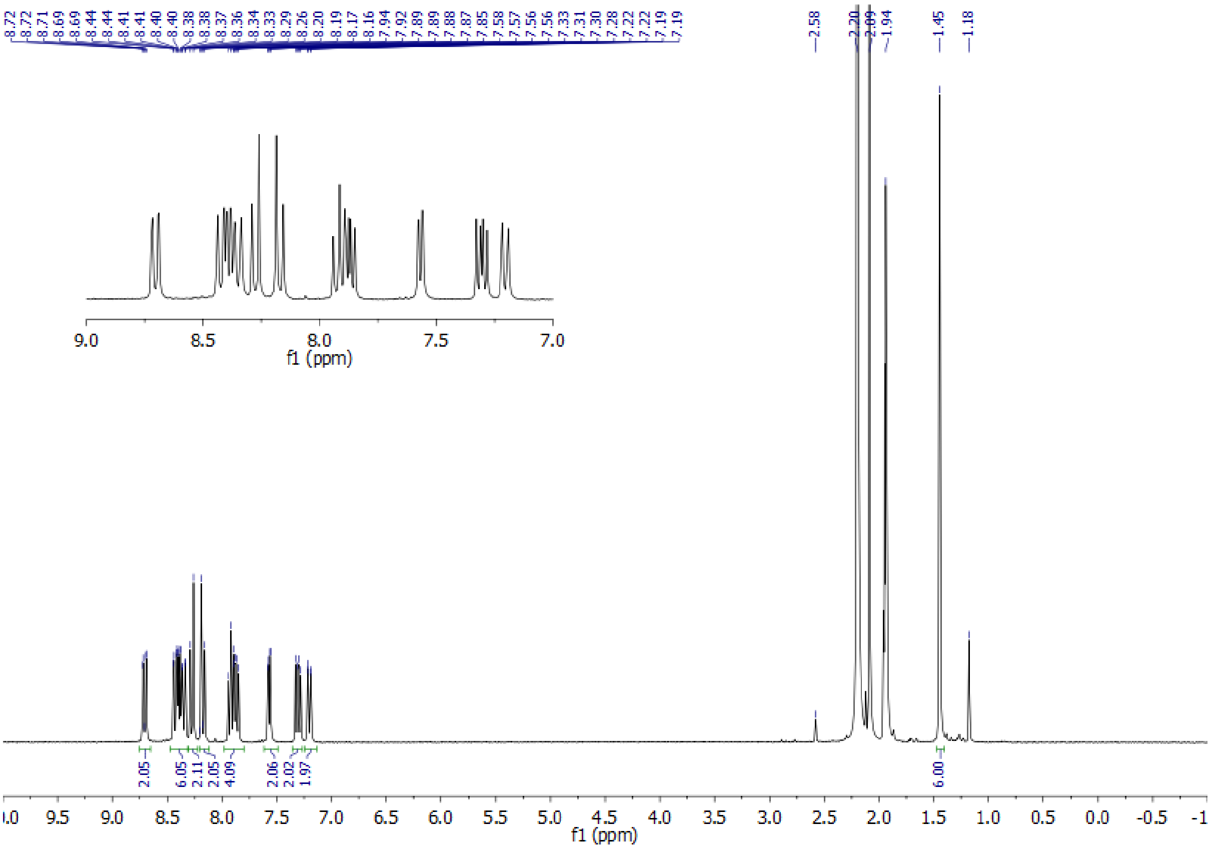

Figure S4. ^{1}H NMR spectrum of compound [1b](PF_6)$_2$ in CD_3CN.

[Ru(phen)$_2$(dmphen)](PF_6)$_2$, [2b](PF_6)$_2$: Compound [**2b**](PF_6)$_2$ was prepared by the same procedure used for [**1a**](PF_6)$_2$, using *cis*-[Ru(phen)$_2$Cl$_2$] ([**3**], 100 mg, 0.188 mmol) and 2,9-dimethyl-1,10phenanthroline (36 mg, 0.172 mmol). Yield: 113 mg (0.118 mmol, 68%). ^{1}H NMR (300 MHz, δ in CD_3CN): 8.64 (dd, J = 8.3, 1.3 Hz, 2H, H_4), 8.47 (d, J = 8.3 Hz, 2H, $H_{4'}$), 8.46 (dd, J = 8.2, 1.1 Hz, 2H, H_7), 8.27 (d, J = 8.9 Hz, 2H, H_5), 8.19 (d, J = 8.9 Hz, 2H, H_6), 8.13 (s, 2H, $H_{5'}$), 8.09 (dd, J = 5.3, 1.3 Hz, 2H, H_2), 7.67 (dd, J = 8.5, 5.6 Hz, 2H, H_3), 7.64 (dd, J = 5.6, 1.5 Hz, 2H, H_9), 7.52 (d, J = 8.3 Hz, 2H, $H_{3'}$), 7.35 (dd, J = 8.2, 5.3 Hz, 2H, H_8), 1.68 (s, 6H, –CH_3); ^{13}C NMR (75 MHz, δ in CD_3CN): 168.4 (C_q), 155.2 (C_9), 153.9 (C_2), 138.5 (C_7), 137.8 ($C_{4'}$), 137.7 (C_4), 132.2 (C_q), 131.9 (C_q), 130.5 (C_q), 129.1 (C_5), 129.0 (C_6), 128.4 ($C_{3'}$), 128.0 ($C_{5'}$), 126.8 (C_3), 126.5 (C_8), 26.2 (–CH_3); HR-MS (CH_3CN) m/z exp. (calc.): 335.07091 (335.07095, [M–2PF$_6$]$^{2+}$); Elemental analysis for $C_{38}H_{28}F_{12}N_6P_2Ru{\cdot}4H_2O$: (calc.): C, 44.24; H, 3.52; N, 8.15; (exp.): C, 44.74; H, 3.43; N, 7.74; UVVis: λ_{max} in nm (ε in $M^{-1}{\cdot}cm^{-1}$): 448 (1.57×10^4) in CH_3CN, 449 (1.66×10^4) in acetone/H_2O (1:1).

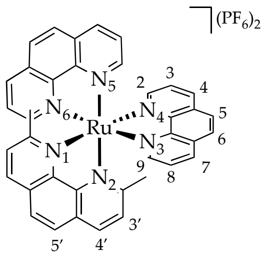

[2b](PF_6)$_2$

Scheme S1. Structural formula of compound [2b](PF_6)$_2$, showing the atom numbering used in NMR attribution.

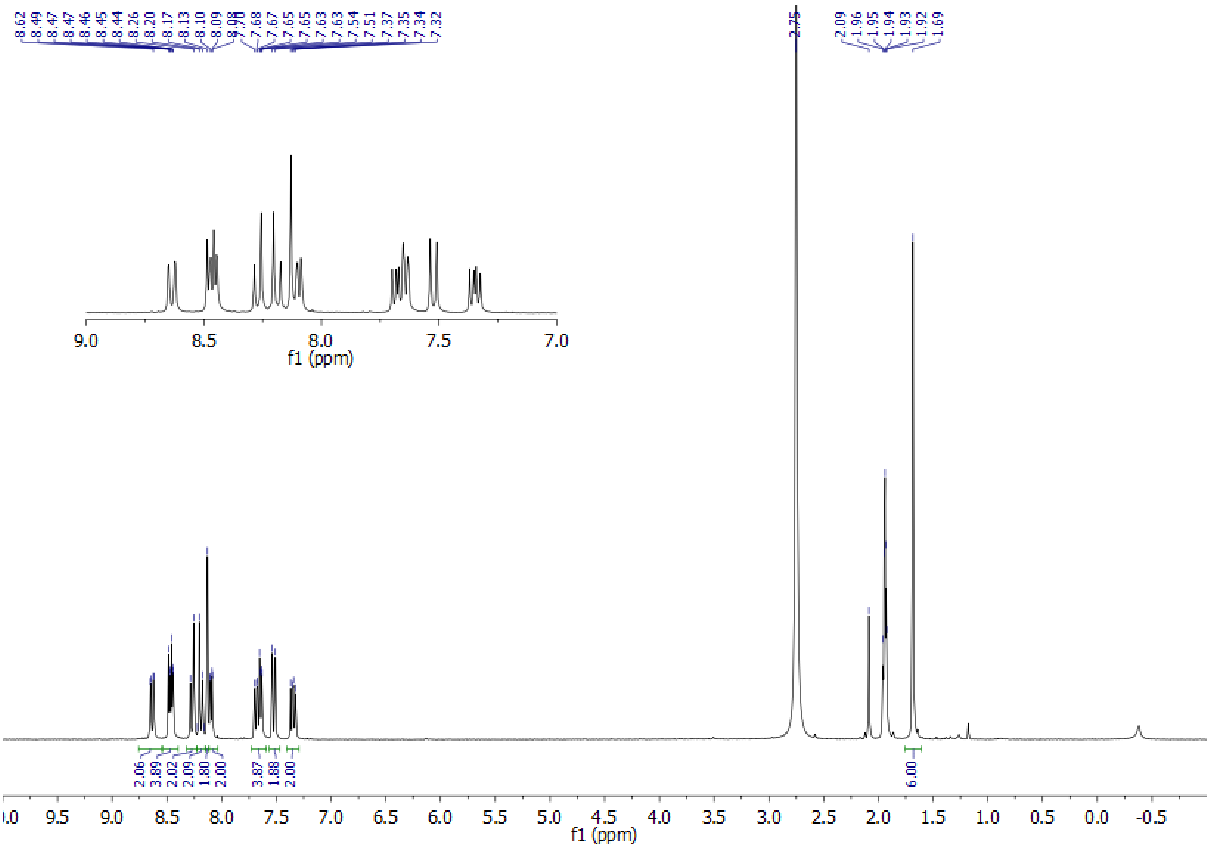

Figure S5. ^{1}H NMR spectrum of compound [2b](PF$_6$)$_2$ in CD$_3$CN.

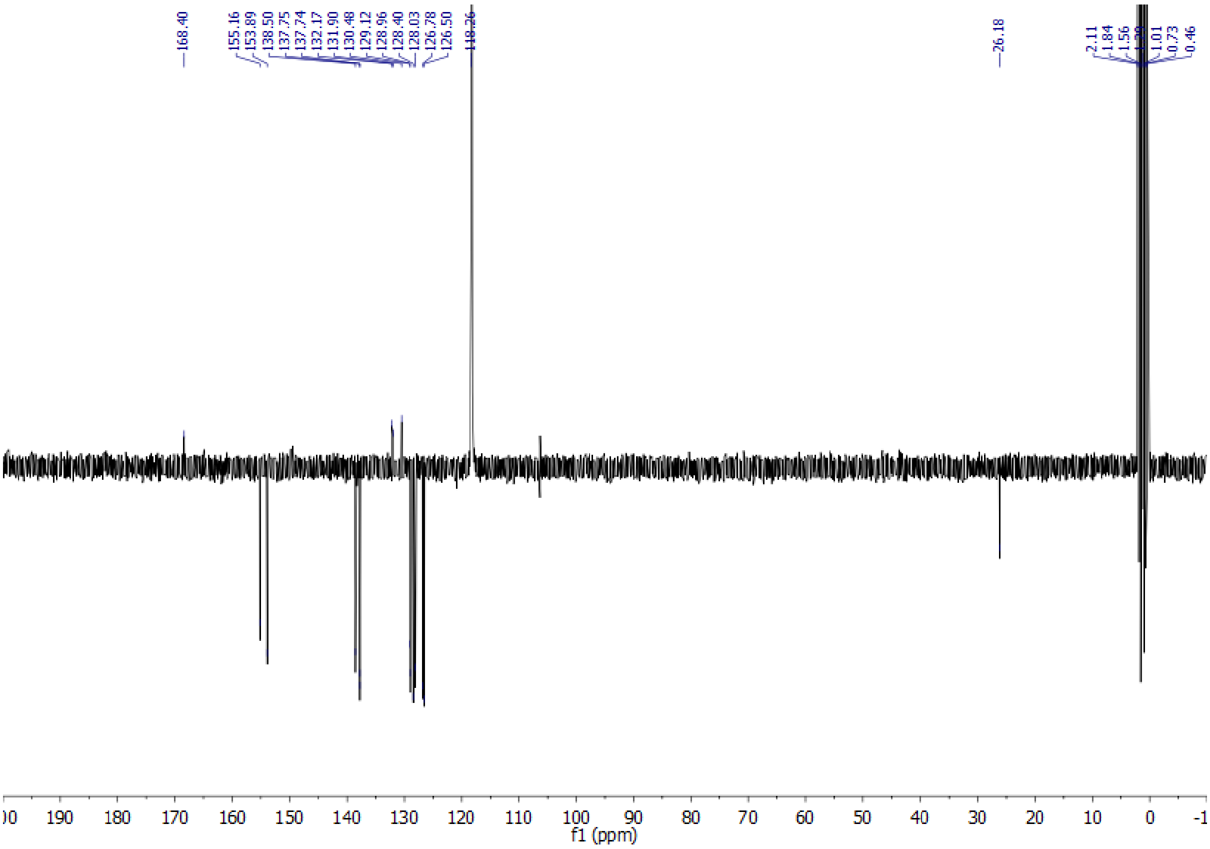

Figure S6. ^{13}C-APT NMR spectrum of compound [2b](PF_6)$_2$ in CD_3CN.

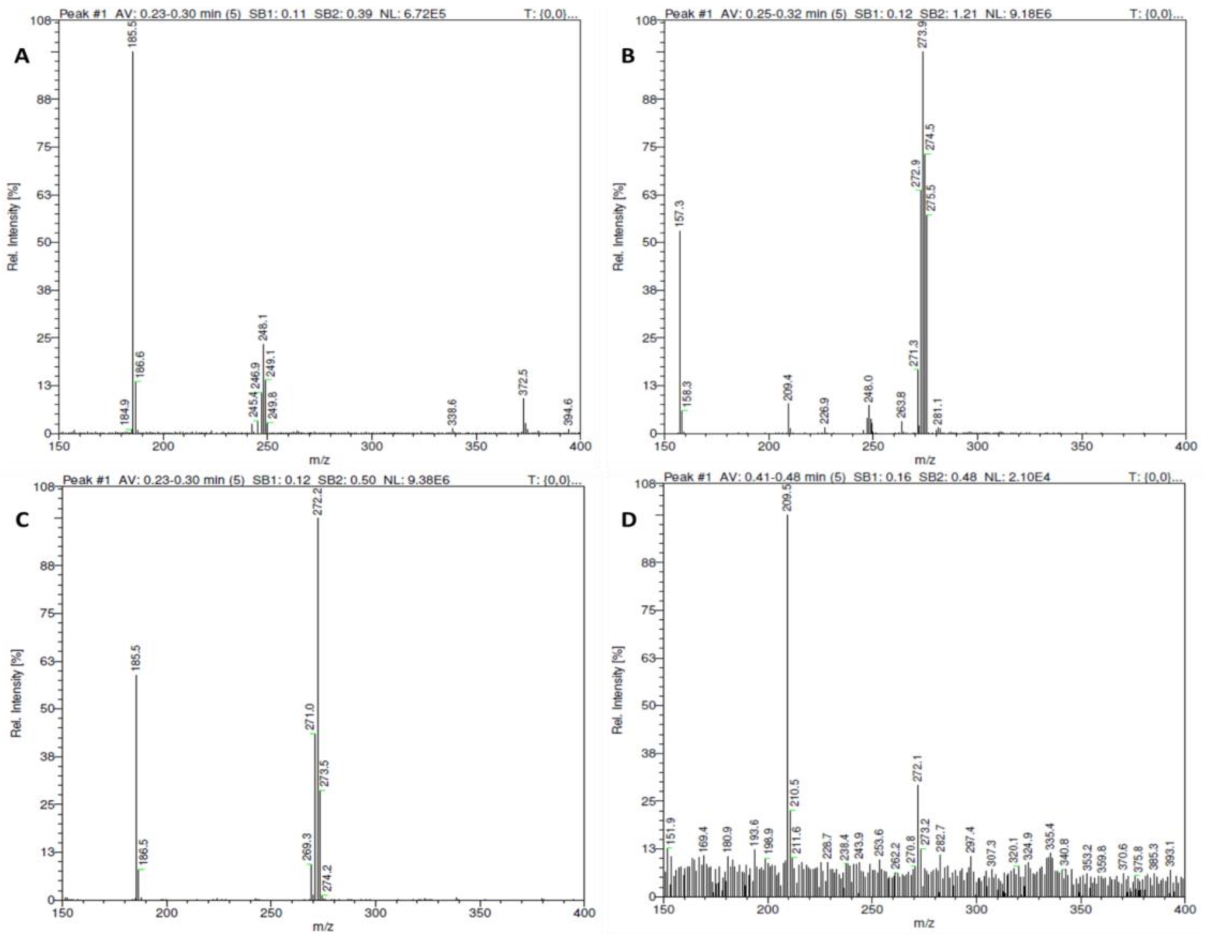

Figure S7. Mass spectra (m/z range = 150–400) of a solution of (A) [1a](PF_6)$_2$, (B) [2a](PF_6)$_2$, (C) [1b](PF_6)$_2$, and (D) [2b](PF_6)$_2$ in CH_3CN after photolysis under N_2 at 298 K, with peaks corresponding to {bpy + H}$^+$ (calcd. m/z = 157.1), {dmbpy + H}$^+$ (calcd. m/z = 185.1), {dmphen + H}$^+$ (calcd. m/z = 209.1), [Ru(bpy)$_2$(CH_3CN)$_2$]$^{2+}$ (calcd. m/z = 248.0), [Ru(phen)$_2$(CH_3CN)$_2$]$^{2+}$ (calcd. m/z = 272.0), and [Ru(bpy)(dmphen)(CH_3CN)$_2$]$^{2+}$ (calcd. m/z = 274.0).

[Ru(phen)$_2$(CO_3)], [4]: Complex [**3**] (50 mg, 0.094 mmol) was in placed in a 25-mL round bottom flask under N_2 atmosphere, and suspended in deoxygenated H_2O (4 mL). The mixture was refluxed for 15 min to obtain a clear solution, to which Na_2CO_3 (430 mg, 1.50 mmol) was added. After a further 3 h of reflux, and subsequent cooling to 0 °C, a black precipitate was obtained, which was collected by filtration and washed with cold water (2 Å~ 10 mL). After drying under vacuum [**3**] was obtained as a black powder (30 mg, 0.058 mmol, 61%). ^{1}H NMR (400 MHz, δ in DMSO-d$_6$): 9.54 (d, J = 5.2 Hz, 2H), 8.73 (d, J = 8.2 Hz, 2H), 8.34 (d, J = 8.9 Hz, 2H), 8.30 – 8.22 (m, 4H), 8.20 (d, J = 8.9 Hz, 2H), 7.72 (d, J = 5.4 Hz, 2H), 7.34 (dd, J = 8.1, 5.4 Hz, 2H).

cis-[Ru(phen)$_2$(CH$_3$CN)$_2$](CF$_3$SO$_3$)$_2$, [5](CF$_3$SO$_3$)$_2$: To a suspension of [**4**] (21 mg, 0.040 mmol) in dry, deoxygenated CH_3CN (4 mL) under N_2 atmosphere was added a drop of trifluoromethylsulfonic acid (~ 0.05 mL). Immediately, gas evolution was observed, and the reaction mixture changed from a black suspension to a yellow-orange solution. The mixture was allowed to stir for another 15 min, after which it was added dropwise to 60 mL of diethyl ether. The resulting yellow precipitate was collected by filtration, washed with diethyl ether (2 Å~ 20 mL) and dried in vacuo. Compound [**5**]$(CF_3SO_3)_2$ was obtained as a yellow powder in 77% yield (26 mg, 0.031 mmol). ^{1}H NMR (400 MHz, δ in CD_3CN): 9.73 (dd, J = 5.2, 1.3 Hz, 2H), 8.86 (dd, J = 8.3, 1.3 Hz, 2H), 8.44 (dd, J = 8.2, 1.3 Hz, 2H), 8.29 (d, J = 8.9 Hz, 2H), 8.22 (dd, J = 8.3, 5.2 Hz, 2H), 8.16 (d, J = 8.9 Hz, 2H), 7.76 (dd, J = 5.3, 1.3 Hz, 2H), 7.42 (dd, J = 8.2, 5.3 Hz, 2H), 2.21 (s, 6H); ^{13}C NMR (101 MHz, δ in CD_3CN): 155.1, 154.1, 138.3, 137.8, 128.8, 127.0, 126.1, 4.6; ESI-MS (CH_3CN) m/z exp. (calc.): 271.8 (272.0, $[M-2CF_3SO_3]^{2+}$).

Single crystal X-ray crystallography of [2a](PF$_6$)$_2$

Single crystals were obtained by slow vapour diffusion of 2-propanol (~ 3 mL) into a solution of [**2a**]$(PF_6)_2$ (1.0 mg, 1.1 μmol) in MeOH (1.0 mL), which was filtered through a 0.2 μm cellulose acetate syringe filter prior to the start of the crystallization. Red, needle-shaped crystals of X-ray quality were obtained after ten days. The structures crystallizes in the C2/*c* space group, unlike the crystal structure of this compound published earlier by Yoshikawa *et al.* (space group $P2_1/c$).[6]

All reflection intensities were measured at 110(2) K using a SuperNova diffractometer (equipped with Atlas detector) with Mo *K*α radiation (λ = 0.71073 Å) under the program CrysAlisPro (Version 1.171.36.32, Agilent Technologies, 2013). The same program was used to refine the cell dimensions and for data reduction. The structure was solved with the program SHELXS-2014/7 (Sheldrick, 2015) and was refined on F^2 with SHELXL-2014/7 (Sheldrick, 2015). Numerical absorption correction based on Gaussian integration over a multifaceted crystal model was applied using CrysAlisPro. The temperature of the data collection was controlled using the system Cryojet (Oxford Instruments). The H atoms were placed at calculated positions using the instructions AFIX 43 or AFIX 137 with isotropic displacement parameters having values 1.2 or 1.5 *U*eq of the attached C atoms. The structure is partly disordered.

The two PF_6^- counter ions are disordered over two orientations, and the occupancy factors of the major components of the disorder refine to 0.875(3) and 0.793(6).

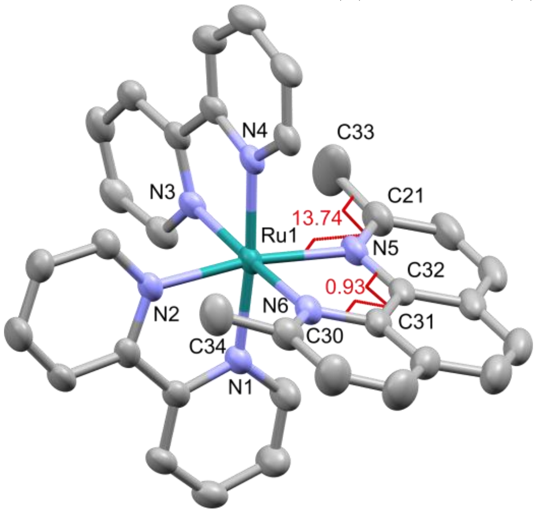

Figure S8. Displacement ellipsoid plot (50% probability level) of the cationic part of [2a](PF_6)$_2$. Counter anions and hydrogen atoms are omitted for clarity. Indicated are the internal twisting torsion angle in the dmphen ligand (N6–C31–C32–N5) and the torsion angle originating from the out-of-plane binding of the dmphen ligand to the ruthenium core (Ru1–N5–C21–C33).

Table S1. Selected bond lengths (Å), bond angles (°), and torsion angles (°) for single crystals of [2a](PF_6)$_2$.

	[2a](PF_6)$_2$
**Ru–N1**	2.069(2)
**Ru–N2**	2.058(2)
**Ru–N3**	2.058(2)
**Ru–N4**	2.050(2)
**Ru–N5**	2.116(2)
**Ru–N6**	2.112(2)
**N1–Ru–N2**	78.65(9)
**N3–Ru–N4**	78.63(9)
**N5–Ru–N6**	79.64(10)
**Ru–N5–C21–C33**	13.7(5)
**N6–C31–C32–N5**	0.9(4)
**C30–C31–C32–C21**	1.4(8)

Table S2. Crystallographic data of [2a](PF_6)$_2$

**Crystal data**	
**Crystal formula**	$C_{34}H_{28}N_6Ru{\cdot}2(F_6P)$
$\boldsymbol{M_r}$	911.64
**Colour**	Red
**Crystal system, space group**	Monoclinic, *C*2/*c*
**Temperature (K)**	110
***a*, *b*, *c* (Å)**	26.3606(7), 9.8635(4), 26.6384(9)
**β (°)**	90.480(3)
***V* (Å^3)**	6925.9(4)
Z	8
**Radiation type**	Mo *K*α
***μ* (mm^{-1})**	0.65
**Crystal size (mm)**	0.34 × 0.13 × 0.11
**Data collection**	
**Diffractometer**	SuperNova, Dual, Cu at zero, Atlas
**Absorption correction**	Gaussian *CrysAlis PRO*, Agilent Technologies, Version 1.171.36.32 (release 02082013 CrysAlis171.NET) (compiled 0208-2013,16:46:58). Numerical absorption correction based on Gaussian integration over a multifaceted crystal model
$\boldsymbol{T_{min}, T_{max}}$	0.714, 1.000
**No. of measured, independent and observed [*I* > 2s(*I*)] reflections**	27172, 7938, 6516
$\boldsymbol{R_{int}}$	0.032

**(sin *q*/*l*)$_{max}$ (Å^{-1})**	0.649
**Refinement**	
R*[*F*^{2} > 2s(*F*^{2})], *wR*(*F*^{2}), *S	0.043, 0.102, 1.03
**No. of reflections**	7938
**No. of parameters**	626
**No. of restraints**	516
**H-atom treatment**	H-atom parameters constrained
**D*r*$_{max}$, D*r*$_{min}$ (e Å^{-3})**	1.27, −0.48
w	$w = 1/[s^2(F_o^2) + (0.0406P)^2 + 17.0653P]$ where $P = (F_o^2 + 2F_c^2)/3$

Photosubstitution studies monitored by UV-Vis absorption spectroscopy and MS

Table S3. Conditions of the photoreactions monitored by UV-Vis absorption spectroscopy and MS.

Entry	Complex	Solvent	[Ru] (μM)	λ_{exc} (nm)	q_P (mol·s^{-1})	*t* (min)
**1**	[**1a**]$(PF_6)_2$	CH_3CN	94	413	5.38×10^{-8}	15
**2**	[**1a**]$(PF_6)_2$	H_2O/acetone (1:1)	72	466	1.07×10^{-7}	10
**3**	[**2a**]$(PF_6)_2$	CH_3CN	73	413	5.38×10^{-8}	60
**4**	[**2a**]$(PF_6)_2$	H_2O/acetone (1:1)	49	466	1.12×10^{-7}	180
**5**	[**1b**]$(PF_6)_2$	CH_3CN	50	413	5.38×10^{-8}	15
**6**	[**1b**]$(PF_6)_2$	H_2O/acetone (1:1)	64	466	1.07×10^{-7}	10
**7**	[**2b**]$(PF_6)_2$	CH_3CN	48	413	5.38×10^{-8}	180
**8**	[**2b**]$(PF_6)_2$	H_2O/acetone (1:1)	44	466	1.12×10^{-7}	180

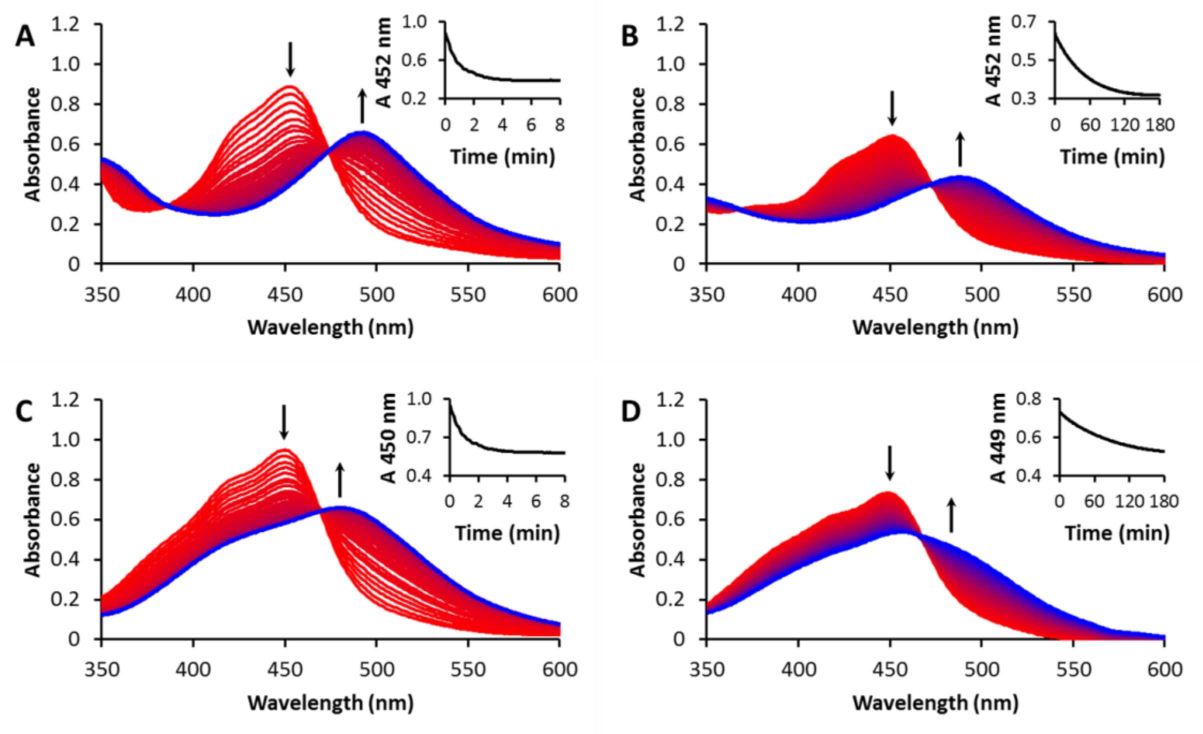

Figure S9. Evolution of the UV-Vis absorption spectra of a solution of (A) [1a](PF_6)$_2$, (B) [2a](PF_6)$_2$, (C) [1b](PF_6)$_2$, and (D) [2b](PF_6)$_2$ in acetone/H_2O (1 : 1, v/v) upon irradiation with a 466 nm LED ($q_p = 1.1 \times 10^{-7}$ mol photons·s^{-1}) under N_2 at 298 K. Conditions: A) 10 min, 72 μM; B) 180 min, 49 μM; C) 10 min, 64 μM; D) 180 min, 44 μM.

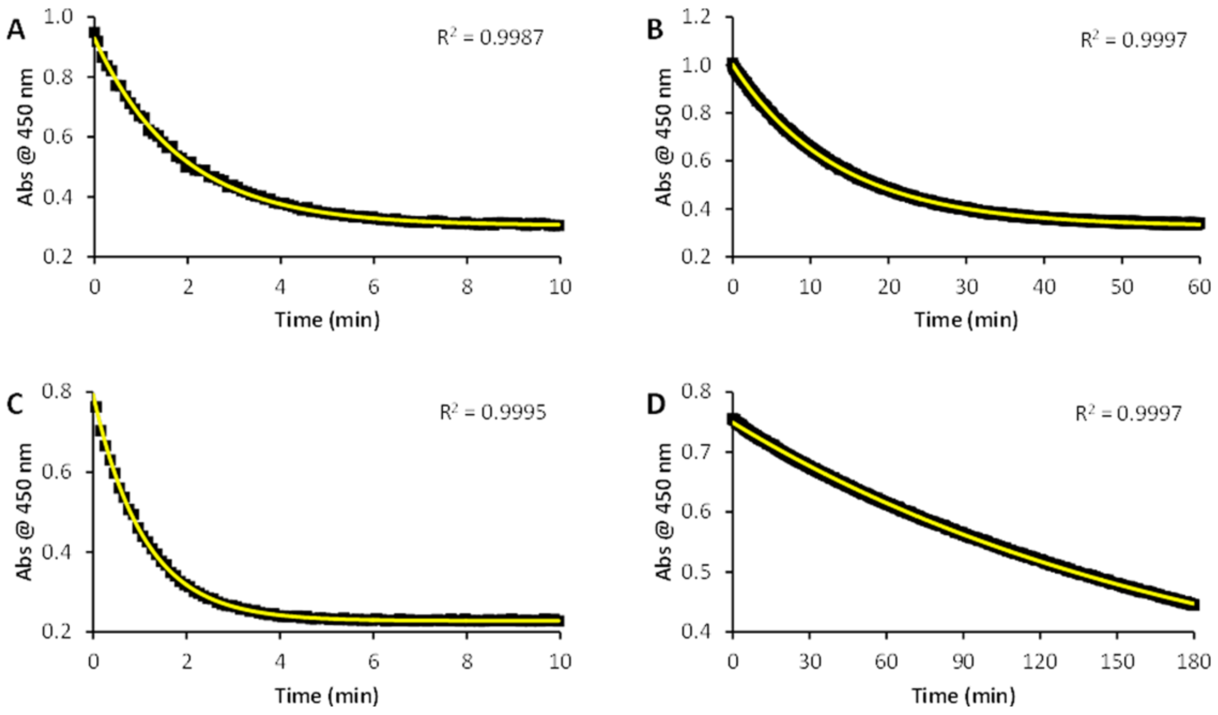

Figure S10. Mono-exponential fitting curves (yellow) of the time evolution of the absorbance at 450 nm (black) during the photolysis in CH_3CN for (A) [1a](PF_6)$_2$, (B) [2a](PF_6)$_2$, (C) [1b](PF_6)$_2$, and (D) [2b](PF_6)$_2$. Conditions: $q_{p,413} = 5.38 \times 10^{-8}$ mol photons·s^{-1}, N_2 atmosphere, T = 298 K, [Ru] = 94 μM (A), 73 μM (B), 50 μM (C), 48 μM (D).

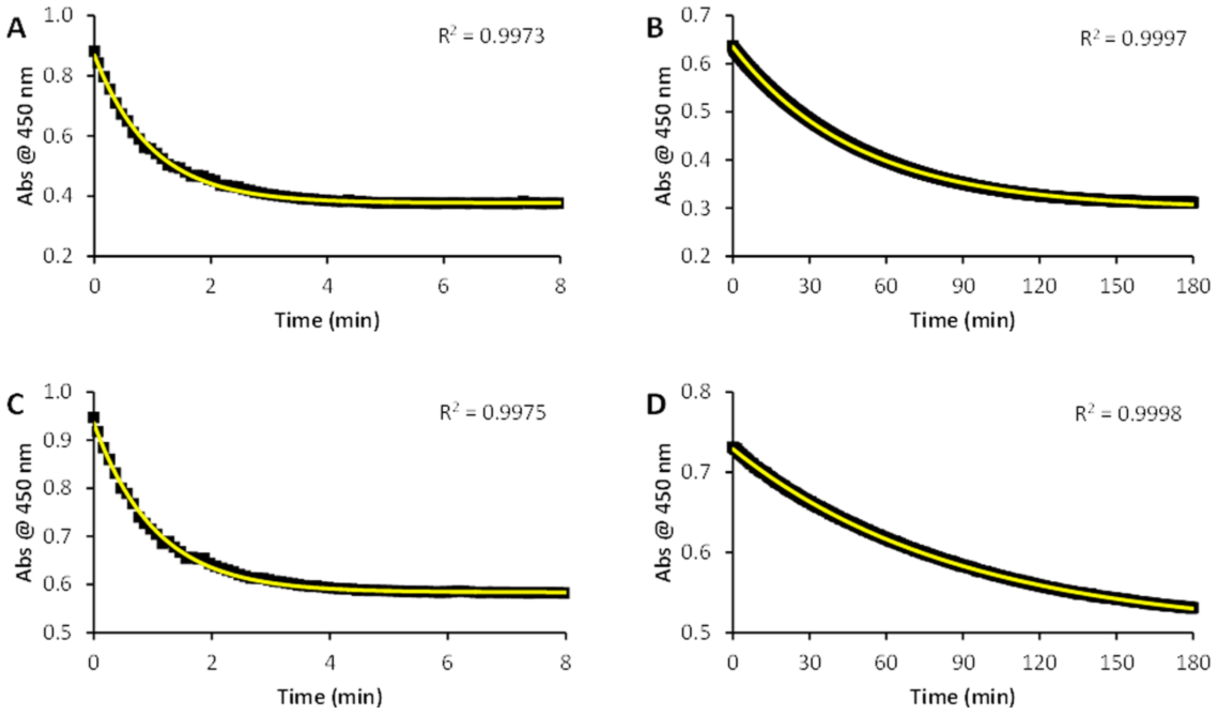

Figure S11. Mono-exponential fitting curves (yellow) of the time evolution of the absorbance at 450 nm (black) during the photolysis in acetone/H_2O (1 : 1) for (A) [1a]$(PF_6)_2$, (B) [2a]$(PF_6)_2$, (C) [1b]$(PF_6)_2$, and (D) [2b]$(PF_6)_2$. Conditions: $q_{p,466}$ = 1.07 × 10^{-7} – 1.12 × 10^{-7} mol photons·s^{-1}, N_2 atmosphere, T = 298 K, [Ru] = 72 µM (A), 49 µM (B), 64 µM (C), 44 µM (D).

Photosubstitution studies monitored by ^{1}H NMR spectroscopy

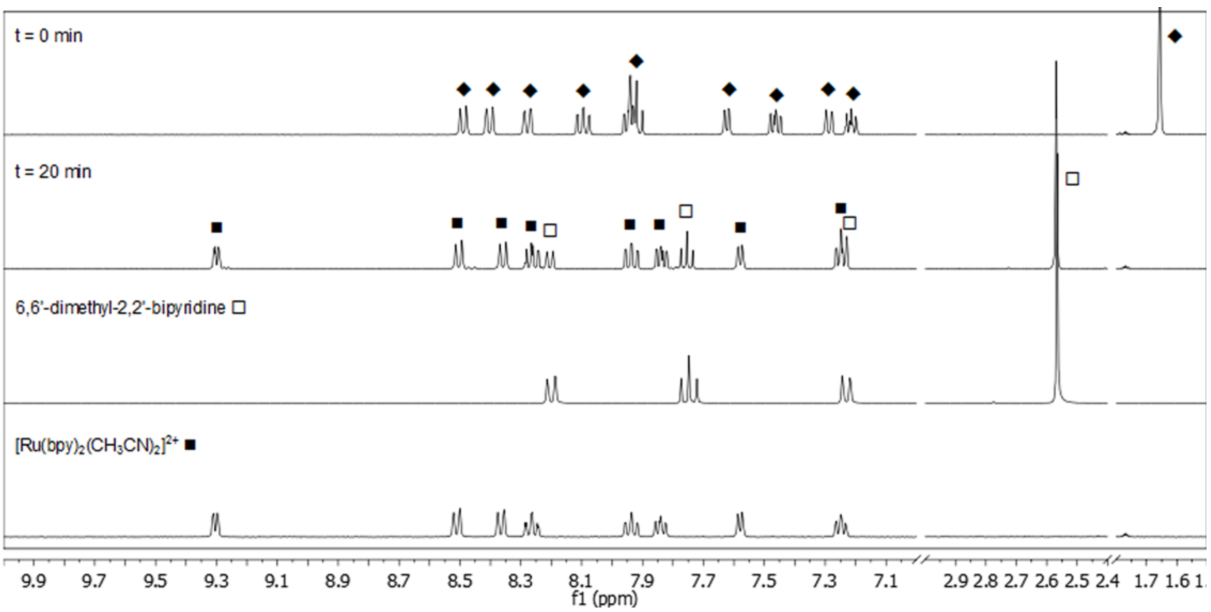

Figure S12. Evolution of the ^{1}H NMR spectra of [1a]$(PF_6)_2$ (◆) under irradiation with white light in CD_3CN at 298 K under N_2, also showing the ^{1}H NMR spectra of the photoproducts 6,6'dimethyl-2,2'bipyridine (□), and *cis*[Ru(bpy)$_2$(CH_3CN)$_2$]$^{2+}$ (■) in CD_3CN.

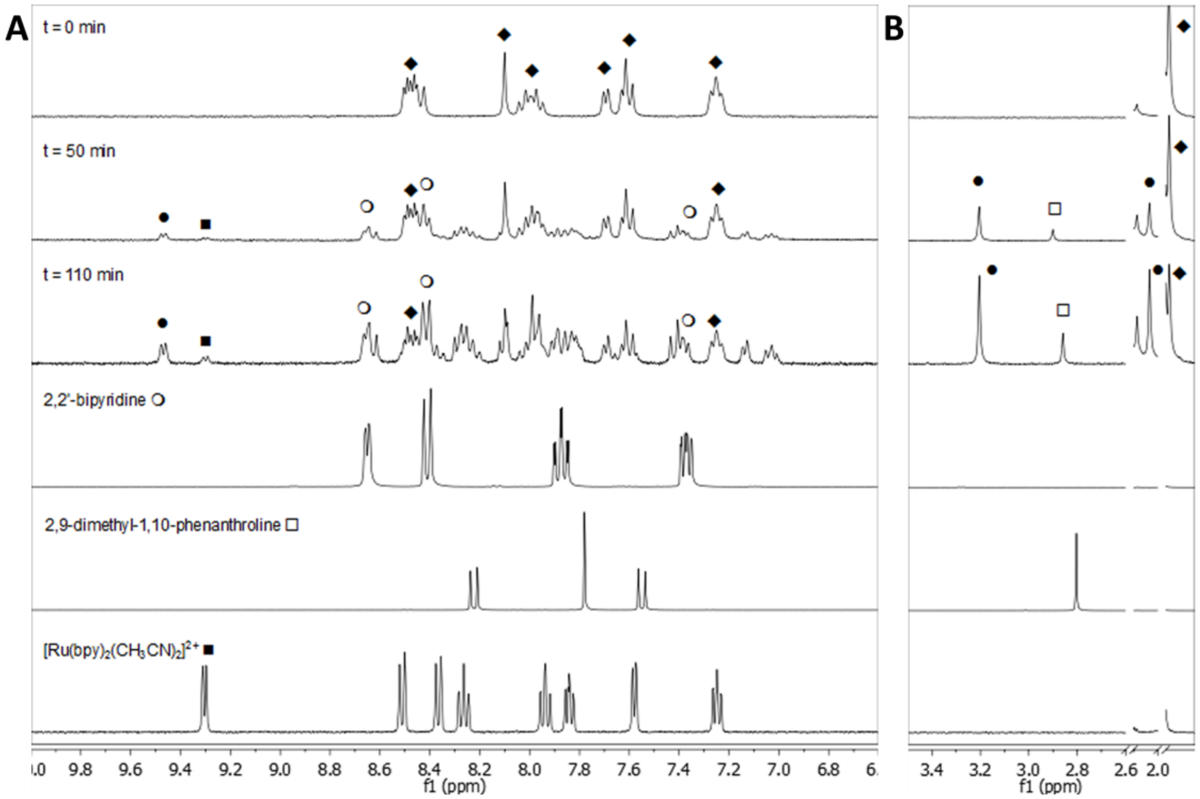

Figure S13. Evolution of (A) the aromatic part, and (B) the aliphatic part of the ${}^1H$ NMR spectrum of [2a]$(PF_6)_2$ (◆) under irradiation with white light in CD_3CN at 298 K under N_2, also showing the ${}^1H$ NMR spectra of the photoproducts 2,2'-bipyridine (○), 2,9-dimethyl-1,10phenanthroline (☐), and *cis*[Ru(bpy)$_2$(CD_3CN)$_2$]$^{2+}$ (■) in CD_3CN. Peaks belonging to the photoproduct *cis*[Ru(bpy)(dmphen)(CD_3CN)$_2$]$^{2+}$ are labelled as ●. Parts A and B are not to scale.

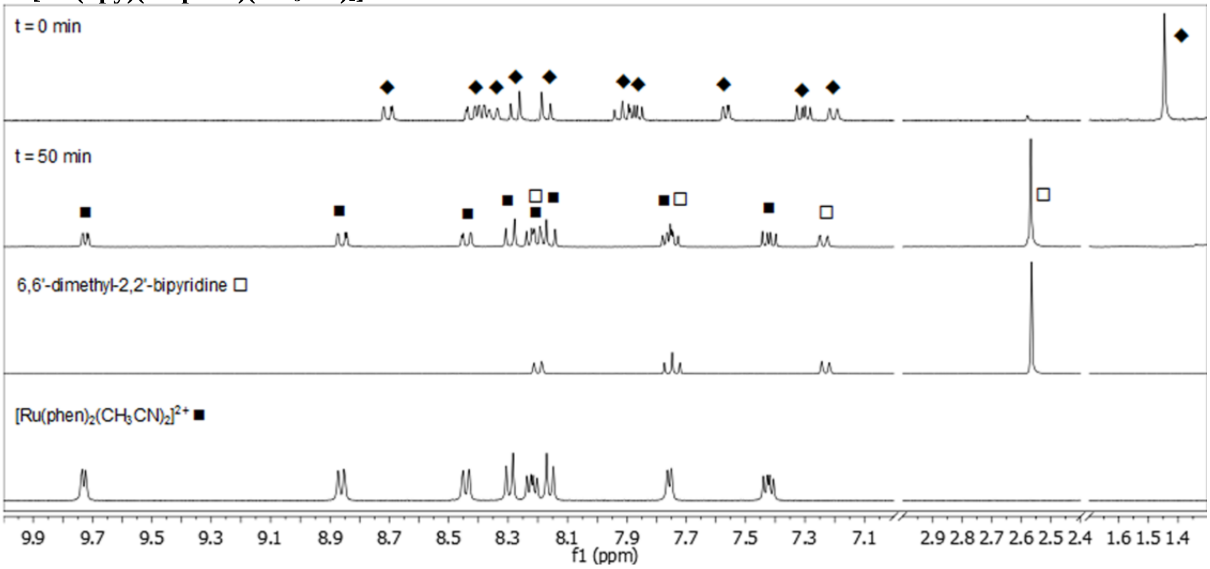

Figure S14. Evolution of the ${}^1H$ NMR spectra of [1b]$(PF_6)_2$ (◆) under irradiation with white light in CD_3CN at 298 K under N_2, also showing the ${}^1H$ NMR spectra of the photoproducts 6,6'dimethyl-2,2'bipyridine (☐), and *cis*[Ru(phen)$_2$(CH_3CN)$_2$]$^{2+}$ (■) in CD_3CN.

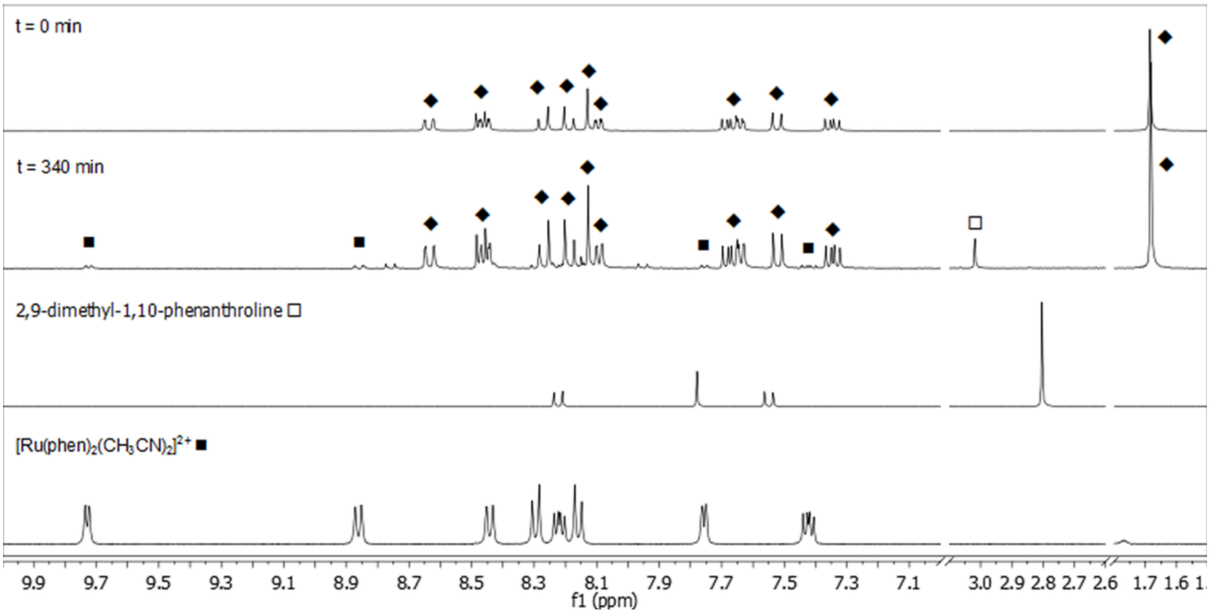

Figure S15. Evolution of the ^{1}H NMR spectra of [2b]$(PF_6)_2$ (◆) under irradiation with white light in CD_3CN at 298 K under N_2, also showing the ^{1}H NMR spectra of the photoproducts 2,9dimethyl-1,10phenanthroline (□), and *cis*[Ru(phen)$_2$(CH_3CN)$_2$]$^{2+}$ (■) in CD_3CN. At low concentrations, the –CH_3 peak in 2,9-dimethyl-1,10phenanthroline appears slightly more downfield that at high concentrations.

Singlet oxygen generation and phosphorescence quantum yield of [1a–2b]$(PF_6)_2$

The quantum yields of singlet oxygen generation and phosphorescence were determined in a custom-built setup described below (Scheme S1). 500 µL of sample, consisting of the compound in deuterated methanol, was added to a 104F-OS semi-micro fluorescence cuvette from Hellma Analytics, and placed in the temperature-controlled cuvette holder. The sample was allowed to equilibrate at 298 K for 5 minutes. Emission spectroscopy was performed with a 450 nm fibre-coupled laser (LRD-0450, Laserglow), which was set to 50 mW at the cuvette (4 mm beam diameter; 0.4 W·cm^{-2}) at a 90° angle with respect to the spectrometer. The excitation power was measured using a S310C thermal sensor connected to a PM100USB power meter (Thorlabs). The emission spectra were recorded using two separate spectrometers for the UV-Vis and NIR emission, i.e. from 300 nm to 1000 nm for the phosphorescence of the complex (Avantes 2048L StarLine spectrometer) and from 1000 nm to 1700 nm for the phosphorescence of singlet oxygen ($^1\Delta_g$) around 1275 nm (Avantes NIR256-1.7TEC spectrometer, detector set to −12 °C). The infrared emission spectrum was acquired within 9 seconds, after which the laser was turned off directly. Similarly, the visible emission spectrum was acquired within 2 seconds. UV-Vis absorption spectra before and after emission spectroscopy were measured using an Avalight-DHc halogen-deuterium lamp (Avantes) as light source (turned off during emission spectroscopy) and the before mentioned UV-Vis spectrometer as detector, both connected to the cuvette holder at a 180° angle. All spectra were recorded using Avasoft 8.5 software from Avantes and further processed using Microsoft Office Excel 2010 and Origin Pro 9.1 software.

The quantum yields of phosphorescence and singlet oxygen production was calculated using the relative method with $[Ru(bpy)_3]Cl_2$ as the standard (Φ_Δ = 0.73,[7,8] Φ_P = 0.015 in CD_3OD), according to Equation S2. The phosphorescence quantum yield of $[Ru(bpy)_3Cl_2]$ in CD_3OD was determined by absolute methods (see below).

$$\text{(S2)}\quad \Phi_{\mathrm{sam}} = \Phi_{\mathrm{std}} \times \frac{A_{std}^{450}}{A_{sam}^{450}} \times \frac{E_{sam}}{E_{std}}$$

where Φ is the quantum yield, A^{450} is the absorbance at 450 nm (always kept below 0.1 for a 4 mm path length), E is the integrated emission peak of singlet oxygen at 1270 nm or the integrated phosphorescence emission peak between 520 and 950 nm, and *sam* and *std* denote the sample and standard, respectively.

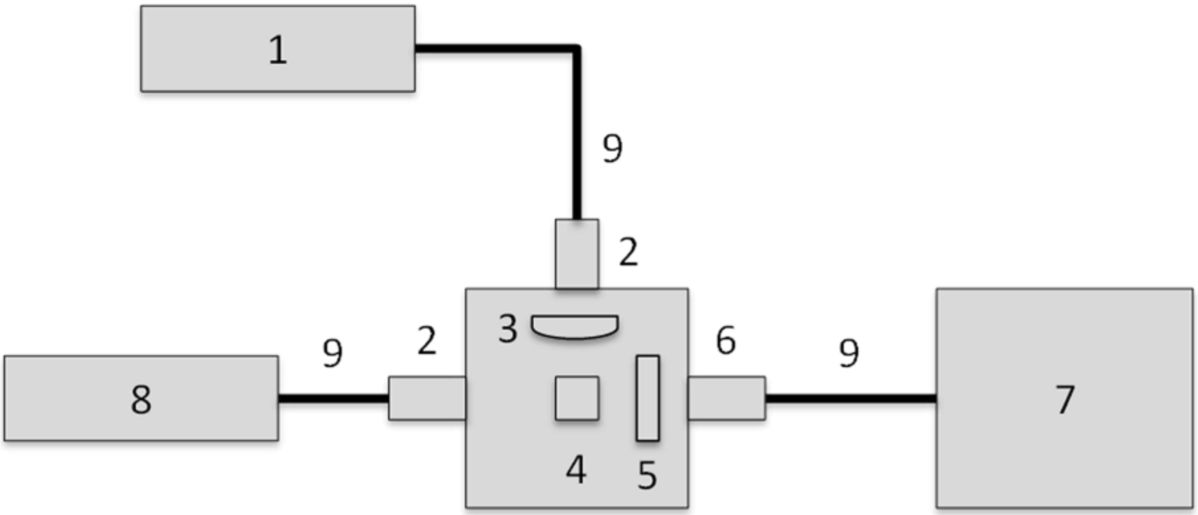

Scheme S2. Setup used for phosphorescence and singlet oxygen quantum yield experiments. (1) CW laser light source, (2) collimating lens, (3) focusing lenses (only for NIR excitation), (4) temperature-controlled cuvette holder, (5) short pass filter (only for emission spectra under NIR excitation), (6) double collimator, (7) UV-Vis (300–1000 nm) or NIR (1000–1700 nm) CCD spectrometer, (8) UV-Vis halogen-deuterium light source, and (9) optical fibres.

Absolute phosphorescence quantum yield of $[Ru(bpy)_3Cl_2]$

The phosphorescence quantum yield (Φ_P) of $[Ru(bpy)_3Cl_2]$ in deuterated methanol in air at room temperature (293 ± 2 K) was determined by absolute methods using an integrating sphere-based setup, to serve as a standard for relative phosphorescence quantum yield measurements. Φ_P was found to be 0.015 ± 0.002. In order to validate the method used, Φ_P was also determined in water, where we obtained a Φ_P in air of 0.044 ± 0.005, which compares well to the literature value (0.040 ± 0.002).[9]

Experimental setup: The integrating sphere setup used for determining the phosphorescence quantum yield is depicted in Scheme S2. The excitation source was a fibre-coupled CW 450-nm diode laser (LRD-0450, Laserglow), coupled into a 200-μm multimode optical fibre, leading to a collimating lens (F220SMA-B, Thorlabs). After collimation, the light passed a

mechanical iris to produce a 4-mm diameter beam (*vide infra*) with 10 mW optical power (P_{exc} = 80 mW·cm^{-2}). The excitation power was measured using a S310C thermal sensor connected to a PM100USB power meter (Thorlabs). A PTFE-coated AvaSphere-30-IRRAD integrating sphere (30 mm diameter, reflectance > 98%), fitted with three ports (entry, exit and sample port), and an AvaSpec-ULS2048L StarLine CCD spectrometer were obtained from Avantes. The integrating sphere and spectrometer were calibrated together using an AvalightHALCALISP30 NIST traceable calibration lamp from Avantes (9.5% relative uncertainty *versus* NIST standard), so that the observed intensities are expressed as a photon flux (mol photons·s^{-1}·nm^{-1}). The filter holder was fabricated by our own mechanical department, and held a NE520A (OD = 2) absorptive neutral density filter (Thorlabs) or a 500-nm longpass filter (FEL0500, Thorlabs, $T_{520\text{-}1000\ nm}$ = 92.1 ± 0.9%). An Avalight-DHc (Avantes) deuterium-halogen lamp was used as a white-light source for the determination of the transmission functions of the filters used. The spectra were recorded with Avasoft 8.5 software from Avantes, and further processed with Microsoft Office Excel 2010 and Origin Pro 9.1 software.

Experimental procedure: A measurement tube, made of a quartz EPRtube bottom (± 7 cm length) fused to a NS14 glass connector (± 2 cm length), was filled with a solution of [Ru(bpy)$_3$Cl$_2$] (50 µL, 50 µM in CD_3OD), and closed with a septum under air. A second tube was filled with CD_3OD (50 µL) and served as the blank. The tube precisely fitted into a hole made in the integrating sphere, and was suspended in the centre of the sphere, in the middle of the excitation laser beam. The laser diode was allowed to warm up for 10 minutes prior to the experiment to guarantee a stable optical power output. The measurements were always performed in the same order, i.e. (1) absorption measurement of the blank, (2) absorption measurement of the sample, and (3) emission measurement of the sample. In this way, the neutral density filter is not moved between the measurement of the blank and sample, ensuring equal attenuation of the non-absorbed excitation light for both spectra. Equally, the sample is not moved between the measurement of its absorption and emission. For the absorption measurements, the neutral density filter was placed in the filter, and replaced by the 500-nm longpass filter for the emission measurement.

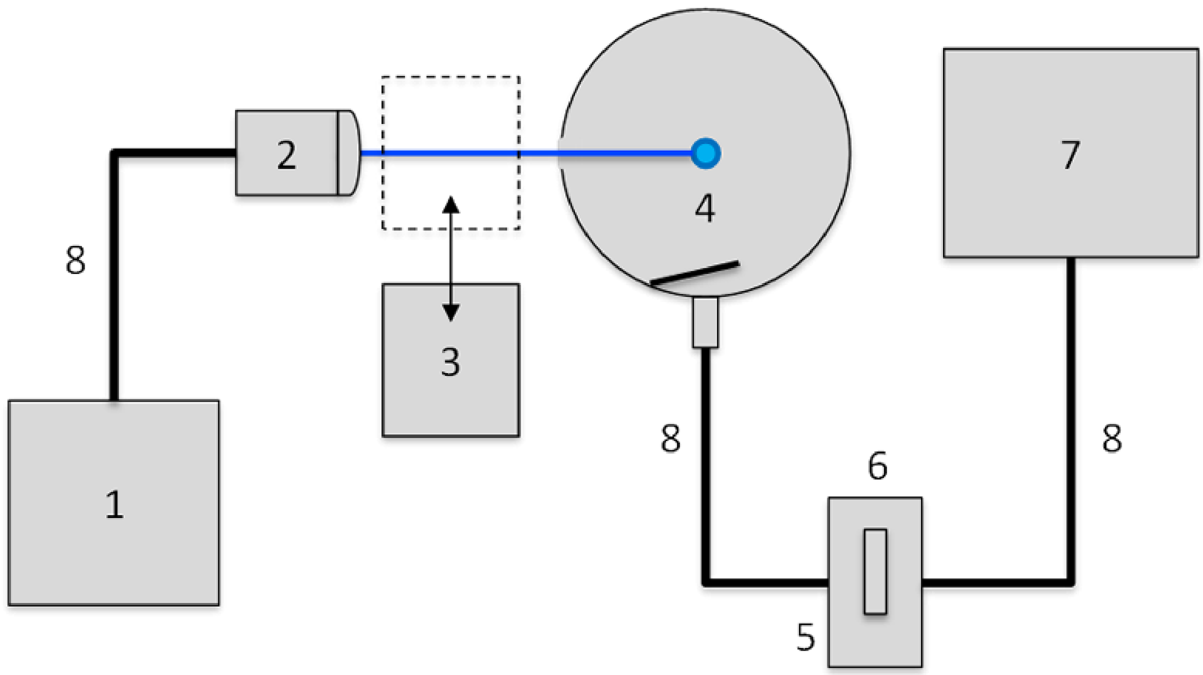

Scheme S3. Setup used for absolute quantum yield measurements. (1) laser source, (2) collimating lens, and mechanical iris, (3) power meter (adjustable in position), (4) integrating sphere with sample tube in the centre, (5) filter holder, (6) 500-nm longpass filter or OD2 neutral density filter, (7) CCD spectrometer, (8) optical fibres.

Quantum yield calculation method: The phosphorescence quantum yield (Φ_P) is defined by Equation S3:

$$\text{(S3) } \Phi_P = \frac{q_{\text{p-em}}}{q_{\text{p-abs}}} = \frac{\int_{\lambda_1}^{\lambda_2} I_P(\lambda)d\lambda}{\int_{\lambda_3}^{\lambda_4}(I_{\text{exc-blank}}(\lambda) - I_{\text{exc-sample}}(\lambda))d\lambda}$$

Here, q_{pem} is the emission photon flux (in photons·s^{-1}) integrated over the spectral range λ_1 to λ_2 (520–950 nm), q_{pabs} is the photon flux absorbed by the ruthenium complex (in photons·s^{-1}), and $I_P(\lambda)$ is the spectral luminescence intensity (in photons·s^{-1}·nm^{-1}). q_{pabs} is determined by subtracting the spectral light intensity of the excitation source that has passed through the sample ($I_{excsample}$, in photons·s^{-1}·nm^{-1}) from the spectral light intensity of the excitation source that has passed through the blank sample ($I_{excblank}$, in photons·s^{-1}·nm^{-1}), and by integrating over the excitation wavelength range λ_3 to λ_4 (400–500 nm).

The spectrometer and the integrating sphere were calibrated so that the observed intensities are directly proportional to the photon flux, i.e. $I_P(\lambda) \propto$ [mol photons·s^{-1}·nm^{-1}] Therefore, integrating these values over the relevant wavelength regions directly provided the flux of photons that arrived at the spectrometer.

Because the intensity of the emitted light is relatively low compared to that of the exciting laser source the absorption and emission of the sample cannot be measured at the same time. In other words, the laser light saturates the spectrometer, which prevents the emission to be measured simultaneously. To circumvent this problem, the absorption was measured using a neutral density filter with known transmittance (typically $F_{attn} \approx 0.0062$, i.e. ~ 99.4% attenuation). This filter was placed between the integrating sphere and the spectrometer to measure the absorbed photon flux. The data was corrected for the attenuation by the neutral density filter ($F_{attn}(\lambda)$) at each wavelength. For the measurement of the emission, this filter was replaced by a longpass filter (> 500 nm) to remove the excitation light. Additionally, the intensity of the emission measured was corrected for the minimal absorbance of this light by the shortpass filter used. This was performed by dividing the luminescence intensity at each wavelength by the transmission curve *T(λ)* of the longpass filter at this wavelength. The accordingly corrected equation for Φ_{UC} is Equation S4:

$$\text{(S4) } \Phi_P = \frac{\int_{\lambda_1}^{\lambda_2}\left(\frac{I_P(\lambda)}{T(\lambda)}\right)d\lambda}{\int_{\lambda_3}^{\lambda_4}\frac{I_{\text{exc-blank}}(\lambda) - I_{\text{exc-sample}}(\lambda)}{F_{\text{attn}}(\lambda)}d\lambda} \equiv \frac{q_{\text{p-em}}}{q_{p\text{-abs}}}$$

Activation enthalpy of photosubstitution reactions

Table S4. The data obtained from the temperature dependent photosubstitution reaction for complex [1a](PF_6)$_2$ in H_2O:acetone 1:1. The photon flux of the 466 nm LED was determined at 7.21 x 10^{-9} mol·s^{-1}·mW^{-1}.

Temperature (K)	Optical Power (mW)	1/T (K^{-1})	k_{Φ} (s^{-1})	A_{466}	n_{Ru} (mol)	q_p (mol·s^{-1})	Φ	ln (Φ)
283.15	6.24	3.53×10^{-3}	7.65×10^{-3}	0.75	2.49×10^{-7}	4.50×10^{-8}	4.24×10^{-2}	-3.16
285.65	5.84	3.50×10^{-3}	8.66×10^{-3}	0.56	1.95×10^{-7}	4.21×10^{-8}	4.01×10^{-2}	-3.22
288.15	5.75	3.47×10^{-3}	1.02×10^{-2}	0.48	1.89×10^{-7}	4.14×10^{-8}	4.64×10^{-2}	-3.07
290.65	5.90	3.44×10^{-3}	1.35×10^{-2}	0.47	1.88×10^{-7}	4.25×10^{-8}	5.98×10^{-2}	-2.82
290.65	6.77	3.44×10^{-3}	1.21×10^{-2}	0.48	1.94×10^{-7}	4.88×10^{-8}	4.81×10^{-2}	-3.03
293.15	5.76	3.41×10^{-3}	1.31×10^{-2}	0.47	1.87×10^{-7}	4.15×10^{-8}	5.88×10^{-2}	-2.83
295.65	6.03	3.38×10^{-3}	1.42×10^{-2}	0.46	1.87×10^{-7}	4.35×10^{-8}	6.08×10^{-2}	-2.80
298.15	6.15	3.35×10^{-3}	1.52×10^{-2}	0.46	1.85×10^{-7}	4.43×10^{-8}	6.35×10^{-2}	-2.76

Table S5. The data obtained from the temperature dependent photosubstitution reaction for complex [2a](PF_6)$_2$ in H_2O:acetone 1:1. The photon flux of the 466 nm LED was determined at 7.21 x 10^{-9} mol·s^{-1}·mW^{-1}.

Temperature (K)	Optical Power (mW)	1/T (K^{-1})	k_{Φ} (s^{-1})	A_{466}	n_{Ru} (mol)	q_p (mol·s^{-1})	Φ	ln (Φ)
298.15	5.77	3.35×10^{-3}	1.60×10^{-4}	0.72	1.92×10^{-7}	4.16×10^{-8}	7.38×10^{-4}	-7.21
303.15	6.08	3.30×10^{-3}	2.55×10^{-4}	0.69	1.67×10^{-7}	4.38×10^{-8}	9.84×10^{-4}	-6.92
305.65	6.75	3.27×10^{-3}	2.80×10^{-4}	0.56	1.94×10^{-7}	4.87×10^{-8}	1.12×10^{-3}	-6.79
308.15	6.77	3.25×10^{-3}	3.03×10^{-4}	0.47	1.69×10^{-7}	4.88×10^{-8}	1.05×10^{-3}	-6.86
313.15	6.73	3.19×10^{-3}	4.14×10^{-4}	0.50	1.74×10^{-7}	4.85×10^{-8}	1.49×10^{-3}	-6.51
318.15	7.04	3.14×10^{-3}	7.76×10^{-4}	0.50	1.85×10^{-7}	5.07×10^{-8}	2.83×10^{-3}	-5.87
320.65	6.73	3.12×10^{-3}	8.07×10^{-4}	0.56	1.94×10^{-7}	4.85×10^{-8}	3.23×10^{-3}	-5.73
323.15	6.90	3.09×10^{-3}	1.19×10^{-4}	0.48	1.85×10^{-7}	4.97×10^{-8}	4.44×10^{-3}	-5.42

Table S6. The data obtained from the temperature dependent photosubstitution reaction for complex [1a](PF_6)$_2$ in acetonitrile. The photon flux of the 413 nm LED was determined at 9.61 x 10^{-9} mol·s^{-1}·mW^{-1}.

Temperature (K)	Optical Power (mW)	1/T (K^{-1})	k_Φ (s^{-1})	A_{466}	n_{Ru} (mol)	q_p (mol·s^{-1})	Φ	ln (Φ)
280.65	3.06	3.56×10^{-3}	8.07×10^{-3}	0.50	8.00×10^{-8}	2.94×10^{-8}	2.20×10^{-2}	-3.82
283.15	3.02	3.53×10^{-3}	9.07×10^{-3}	0.50	8.02×10^{-8}	2.90×10^{-8}	2.51×10^{-2}	-3.69
285.65	3.07	3.50×10^{-3}	1.02×10^{-2}	0.62	8.09×10^{-8}	2.95×10^{-8}	2.80×10^{-2}	-3.58
285.65	3.10	3.50×10^{-3}	8.78×10^{-3}	0.54	8.42×10^{-8}	2.97×10^{-8}	2.49×10^{-2}	-3.69
288.15	3.04	3.47×10^{-3}	1.05×10^{-2}	0.49	7.94×10^{-8}	2.92×10^{-8}	2.87×10^{-2}	-3.55
290.65	3.14	3.44×10^{-3}	1.25×10^{-2}	0.49	7.85×10^{-8}	3.02×10^{-8}	3.25×10^{-2}	-3.42
293.15	3.03	3.41×10^{-3}	1.29×10^{-2}	0.49	7.84×10^{-8}	2.91×10^{-8}	3.49×10^{-2}	-3.36
295.65	3.08	3.38×10^{-3}	1.33×10^{-2}	0.49	7.66×10^{-8}	2.95×10^{-8}	3.44×10^{-2}	-3.37
295.65	3.15	3.38×10^{-3}	1.43×10^{-2}	0.58	8.47×10^{-8}	3.03×10^{-8}	4.01×10^{-2}	-3.22
298.15	3.06	3.35×10^{-3}	1.60×10^{-2}	0.48	7.49×10^{-8}	2.93×10^{-8}	4.07×10^{-2}	-3.20

Table S7. The data obtained from the temperature dependent photosubstitution reaction for complex [2a](PF_6)$_2$ in acetonitrile. The photon flux of the 413 nm LED was determined at 9.21 x 10^{-9} mol·s^{-1}·mW^{-1}.

Temperature (K)	Optical Power (mW)	1/T (K^{-1})	k_Φ (s^{-1})	A_{466}	n_{Ru} (mol)	q_p (mol·s^{-1})	Φ	ln (Φ)
298.15	6.70	3.35×10^{-3}	2.70×10^{-3}	0.35	1.47×10^{-7}	6.44×10^{-8}	6.16×10^{-3}	-5.09
303.15	5.97	3.30×10^{-3}	2.34×10^{-3}	0.36	1.44×10^{-7}	5.73×10^{-8}	5.18×10^{-3}	-5.14
308.15	5.97	3.25×10^{-3}	2.10×10^{-3}	0.35	1.41×10^{-7}	5.73×10^{-8}	5.16×10^{-3}	-5.27
313.15	5.91	3.19×10^{-3}	1.58×10^{-3}	0.39	1.44×10^{-7}	5.67×10^{-8}	4.02×10^{-3}	-5.52
313.15	5.98	3.19×10^{-3}	1.72×10^{-3}	0.36	1.50×10^{-7}	5.74×10^{-8}	4.49×10^{-3}	-5.41
318.15	5.92	3.14×10^{-3}	1.27×10^{-3}	0.41	1.44×10^{-7}	5.68×10^{-8}	3.23×10^{-3}	-5.73
318.15	5.87	3.14×10^{-3}	1.41×10^{-3}	0.36	1.44×10^{-7}	5.64×10^{-8}	3.60×10^{-3}	-5.63
323.15	5.81	3.09×10^{-3}	1.43×10^{-3}	0.46	1.65×10^{-7}	5.58×10^{-8}	4.24×10^{-3}	-5.46
328.15	5.80	3.05×10^{-3}	1.06×10^{-3}	0.38	1.38×10^{-7}	5.57×10^{-8}	2.64×10^{-3}	-5.94

328.15	5.72	3.05×10^{-3}	1.04×10^{-3}	0.48	1.68×10^{-7}	5.49×10^{-8}	3.17×10^{-3}	-5.75
333.15	5.83	3.00×10^{-3}	1.18×10^{-3}	0.46	1.47×10^{-7}	5.60×10^{-8}	3.10×10^{-3}	-5.77
333.15	5.72	3.00×10^{-3}	9.18×10^{-3}	0.49	1.68×10^{-7}	5.49×10^{-8}	2.81×10^{-3}	-5.87
338.15	5.65	2.96×10^{-3}	8.28×10^{-3}	0.50	1.65×10^{-7}	5.42×10^{-8}	2.52×10^{-3}	-5.98
343.15	5.63	2.91×10^{-3}	6.67×10^{-3}	0.54	1.65×10^{-7}	5.40×10^{-8}	2.04×10^{-3}	-6.20

Computational Supporting Information

Structures Singlet

	Water			Acetonitrile		
Starting Complex	Reaction Pathway 1	Reaction Pathway 2	Reaction Pathway 3	Reaction Pathway 1	Reaction Pathway 2	Reaction Pathway 3
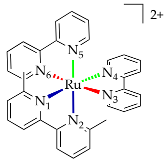 $^{1}[1a]^{2+}$	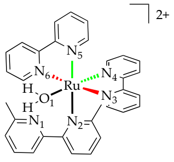 $^{1}[3a]^{2+}$	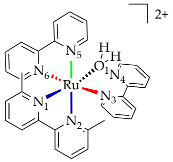 $^{1}[5a]^{2+}$	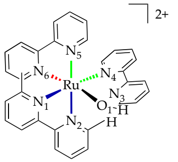 $^{1}[7a]^{2+}$	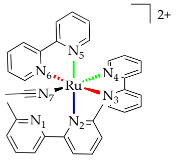 $^{1}[12a]^{2+}$	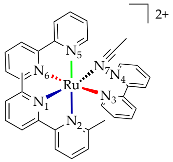 $^{1}[14a]^{2+}$	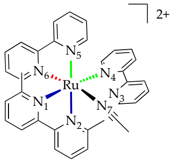 $^{1}[16a]^{2+}$
	Product Pathway 1	Product Pathway 2	Product Pathway 3	Product Pathway 1	Product Pathway 2	Product Pathway 3
	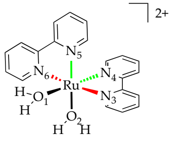 $^{1}[9a]^{2+}$	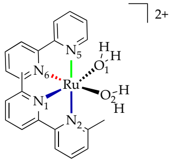 $^{1}[10a]^{2+}$	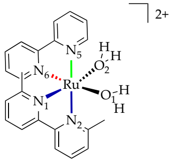 $^{1}[10a]^{2+}$	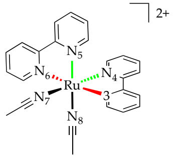 $^{1}[18a]^{2+}$	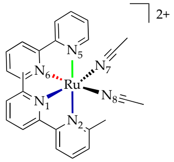 $^{1}[19a]^{2+}$	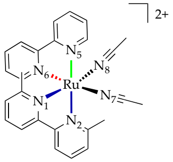 $^{1}[19a]^{2+}$

Starting Complex	Reaction Pathway 1	Reaction Pathway 2	Reaction Pathway 3	Reaction Pathway 1	Reaction Pathway 2	Reaction Pathway 3
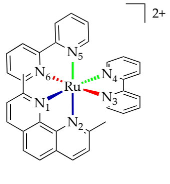 $^{1}[2a]^{2+}$	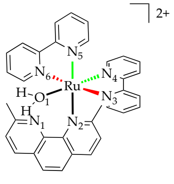 $^{1}[4a]^{2+}$	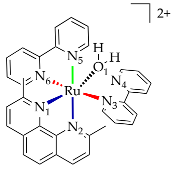 $^{1}[6a]^{2+}$	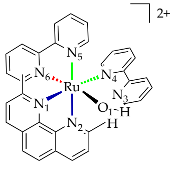 $^{1}[8a]^{2+}$	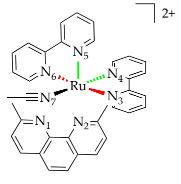 $^{1}[13a]^{2+}$	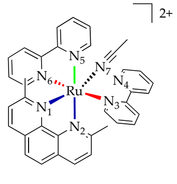 $^{1}[15a]^{2+}$	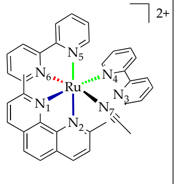 $^{1}[17a]^{2+}$
	Product Pathway 1	Product Pathway 2	Product Pathway 3	Product Pathway 1	Product Pathway 2	Product Pathway 3
	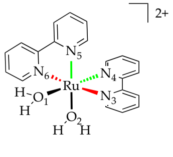 $^{1}[9a]^{2+}$	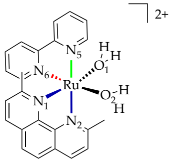 $^{1}[11a]^{2+}$	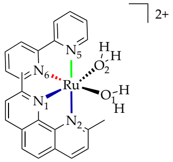 $^{1}[11a]^{2+}$	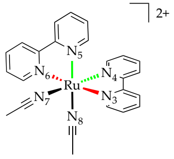 $^{1}[18a]^{2+}$	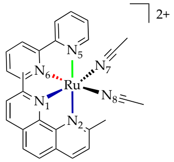 $^{1}[20a]^{2+}$	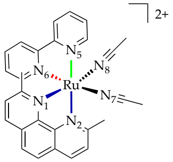 $^{1}[20a]^{2+}$

Scheme S4. The structure of the different singlet states in the photosubstitution reaction of complex $[1a]^{2+}$ and $[2a]^{2+}$ in water and acetonitrile

Table S8. The relative energy to the ground state of the starting complex (kJ/mol), the bond distances (Å), the dihedral angle of the straining ligand (°) and the Mulliken spin on ruthenium of the singlet states found in the photodissociation reaction pathways of complex $^{1}[1a]^{2+}$ in water.

	$^{1}[1a]^{2+}$	$^{1}[3a]^{2+}$	$^{1}[5a]^{2+}$	$^{1}[7a]^{2+}$	$^{1}[9a]^{2+}$	$^{1}[10a]^{2+}$
**Energy vs. $^{1}[1a]^{2+}$**	0	106.0	74.5	69.7	82.9	124.3
**Ru-O1**	-	2.314	2.157	2.169	2.196	2.383
**Ru-N1**	2.108	3.731	2.077	2.088	-	2.076
**Ru-N2**	2.110	2.120	2.088	2.080	-	2.122
**Ru-N3**	2.079	2.095	2.154	3.473	2.055	-
**Ru-N4**	2.074	2.041	3.464	2.177	2.014	-
**Ru-N5**	2.061	2.028	2.073	2.068	2.008	2.087
**Ru-N6**	2.051	2.097	2.066	2.023	2.063	2.032
**N1-C-C-N2**	177.6	204.5	173.6	186.0	-	173.8
**Mulliken spin (Ru)**	-	-	-	-	-	-

Table S9. The relative energy to the ground state of the starting complex (kJ/mol), the bond distances (Å), the dihedral angle of the straining ligand (°) and the Mulliken spin on ruthenium of the singlet states found in the photodissociation reaction pathways of complex $^{1}[2a]^{2+}$ in water.

	$^{1}[2a]^{2+}$	$^{1}[4a]^{2+}$	$^{1}[6a]^{2+}$	$^{1}[8a]^{2+}$	$^{1}[9a]^{2+}$	$^{1}[11a]^{2+}$
**Energy vs. $^{1}[2a]^{2+}$**	0	98.6	77.3	71.3	82.9	315.3
**Ru-O1**	-	2.282	2.156	2.171	2.196	2.209
**Ru-N1**	2.116	3.721	2.085	2.096	-	2.062
**Ru-N2**	2.117	2.655	2.097	2.089	-	2.104
**Ru-N3**	2.077	2.095	2.148	3.465	2.055	-
**Ru-N4**	2.067	2.045	3.458	2.165	2.014	-
**Ru-N5**	2.059	2.020	2.065	2.065	2.008	2.054
**Ru-N6**	2.050	2.089	2.067	2.022	2.063	2.018
**N1-C-C-N2**	179.4	181.6	178.1	182.4	-	178.3
**Mulliken spin (Ru)**	-	-	-	-	-	-

Table S10. The relative energy to the ground state of the starting complex (kJ/mol), the bond distances (Å), the dihedral angle of the straining ligand (°) and the Mulliken spin on ruthenium of the singlet states found in the photodissociation reaction pathways of complex $^{1}[1a]^{2+}$ in acetonitrile.

	$^{1}[1a]^{2+}$	$^{1}[12a]^{2+}$	$^{1}[14a]^{2+}$	$^{1}[16a]^{2+}$	$^{1}[18a]^{2+}$	$^{1}[19a]^{2+}$
**Energy vs. $^{1}[1a]^{2+}$**	0	54.7	56.3	36.5	73.0	85.7
**Ru-N7**	-	2.023	2.017	2.022	2.017	2.015
**Ru-N1**	2.110	3.577	2.110	2.113	-	2.096
**Ru-N2**	2.106	2.224	2.109	2.094	-	2.096
**Ru-N3**	2.051	2.055	2.157	3.500	2.068	-
**Ru-N4**	2.060	2.056	3.924	2.177	2.058	-
**Ru-N5**	2.074	2.064	2.086	2.063	2.059	2.066
**Ru-N6**	2.080	2.081	2.068	2.048	2.069	2.066
**N1-C-C-N2**	177.6	265.8	190.5	184.5	-	173.7
**Mulliken spin (Ru)**	-	-	-	-	-	-

Table S11. The relative energy to the ground state of the starting complex (kJ/mol), the bond distances (Å), the dihedral angle of the straining ligand (°) and the Mulliken spin on ruthenium of the singlet states found in the photodissociation reaction pathways of complex $^{1}[2a]^{2+}$ in acetonitrile.

	$^{1}[2a]^{2+}$	$^{1}[13a]^{2+}$	$^{1}[15a]^{2+}$	$^{1}[17a]^{2+}$	$^{1}[18a]^{2+}$	$^{1}[20a]^{2+}$
**Energy vs. $^{1}[2a]^{2+}$**	0	72.6	56.4	39.5	73.0	89.1
**Ru-N7**	-	2.005	2.017	2.027	2.017	2.022
**Ru-N1**	2.111	3.308	2.114	2.122	-	2.104
**Ru-N2**	2.115	3.400	2.113	2.101	-	2.109
**Ru-N3**	2.080	2.070	2.159	3.518	2.068	-
**Ru-N4**	2.066	1.995	3.912	2.165	2.058	-
**Ru-N5**	2.055	2.057	2.081	2.057	2.059	2.064
**Ru-N6**	2.049	2.068	2.066	2.046	2.069	2.046
**N1-C-C-N2**	179.0	180.3	182.8	181.1	-	179.2
**Mulliken spin (Ru)**	-	-	-	-	73.0	-

Structures Triplet

	Water			Acetonitrile		
Starting Complex	Reaction Pathway 1	Reaction Pathway 2	Reaction Pathway 3	Reaction Pathway 1	Reaction Pathway 2	Reaction Pathway 3
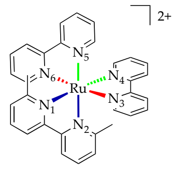 ^{3}[1a]$^{2+}$	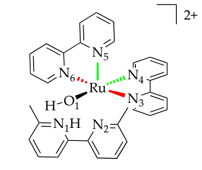 ^{3}[3a]$^{2+}$	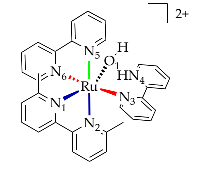 ^{3}[5a]$^{2+}$	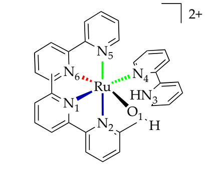 ^{3}[7a]$^{2+}$	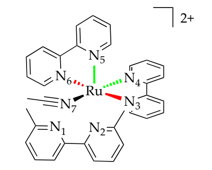 ^{3}[12a]$^{2+}$	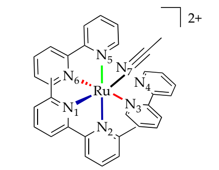 ^{3}[14a]$^{2+}$	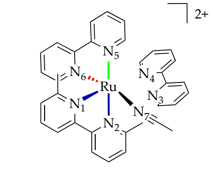 ^{3}[16a]$^{2+}$
	Product Pathway 1	Product Pathway 2	Product Pathway 3	Product Pathway 1	Product Pathway 2	Product Pathway 3
	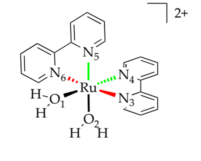 ^{3}[9a]$^{2+}$	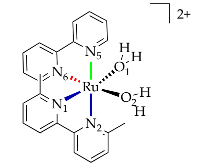 ^{3}[10a]$^{2+}$	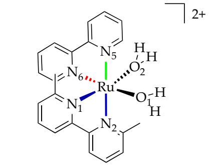 ^{3}[10a]$^{2+}$	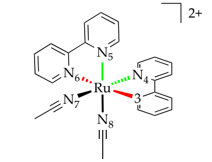 ^{3}[18a]$^{2+}$	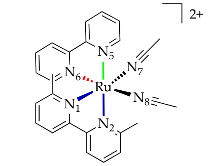 ^{3}[19a]$^{2+}$	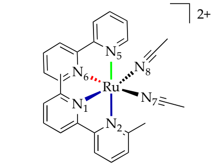 ^{3}[19a]$^{2+}$

Starting Complex	Reaction Pathway 1	Reaction Pathway 2	Reaction Pathway 3	Reaction Pathway 1	Reaction Pathway 2	Reaction Pathway 3
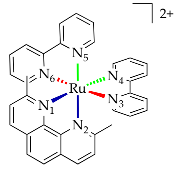 ^{3}[2a]$^{2+}$	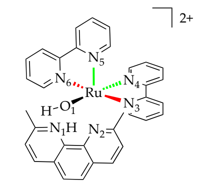 ^{3}[4a]$^{2+}$	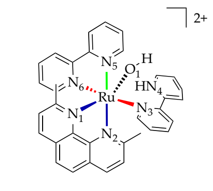 ^{3}[6a]$^{2+}$	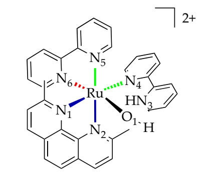 ^{3}[8a]$^{2+}$	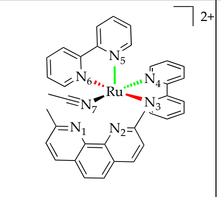 ^{3}[13a]$^{2+}$	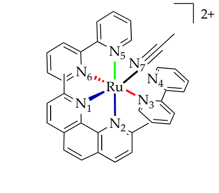 ^{3}[15a]$^{2+}$	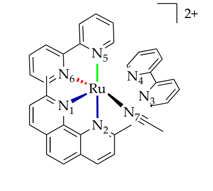 ^{3}[17a]$^{2+}$
	Product Pathway 1	Product Pathway 2	Product Pathway 3	Product Pathway 1	Product Pathway 2	Product Pathway 3
	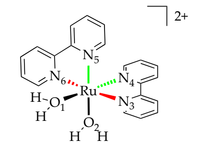 ^{3}[9a]$^{2+}$	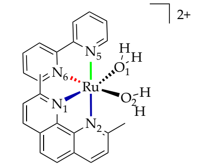 ^{3}[11a]$^{2+}$	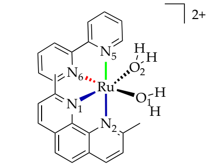 ^{3}[11a]$^{2+}$	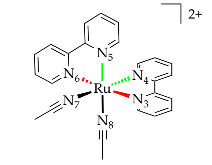 ^{3}[18a]$^{2+}$	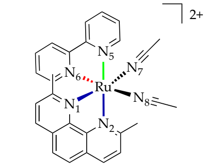 ^{3}[20a]$^{2+}$	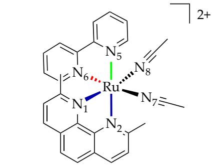 ^{3}[20a]$^{2+}$

Scheme S5. The structure of the different triplet states in the photosubstitution reaction of complex [1a]$^{2+}$ and [2a]$^{2+}$ in water and acetonitrile

Table S12. The relative energy to the ground state of the starting complex (kJ/mol), the bond distances (Å), the dihedral angle of the straining ligand (°) and the Mulliken spin on ruthenium of the triplet states found in the photodissociation reaction pathways of complex ^{1}[1a]$^{2+}$ in water.

	^{3}[1a]$^{2+}$	^{3}[3a]$^{2+}$	^{3}[5a]$^{2+}$	^{3}[7a]$^{2+}$	^{3}[9a]$^{2+}$	^{3}[10a]$^{2+}$
**Energy vs. ^{1}[1a]$^{2+}$**	194.2	214.5	237.2	233.1	439.4	460.1
**Ru-O1**	-	1.986	1.995	2.007	2.434	2.516
**Ru-N1**	2.097	3.545	2.121	2.099	-	2.281
**Ru-N2**	2.094	3.180	2.120	2.078	-	2.122
**Ru-N3**	2.082	2.056	2.194	3.447	2.057	-
**Ru-N4**	2.066	2.092	3.434	2.156	2.081	-
**Ru-N5**	2.080	2.159	2.070	2.095	2.085	2.058
**Ru-N6**	2.064	2.079	2.057	2.077	2.063	2.047
**N1-C-C-N2**	176.8	181.4	172.6	182.2	-	147.6
**Mulliken spin (Ru)**	0.900	1.582	0.815	0.803	1.663	1.673

Table S13. The relative energy to the ground state of the starting complex (kJ/mol), the bond distances (Å), the dihedral angle of the straining ligand (°) and the Mulliken spin on ruthenium of the triplet states found in the photodissociation reaction pathways of complex ^{1}[2a]$^{2+}$ in water.

	^{3}[2a]$^{2+}$	^{3}[4a]$^{2+}$	^{3}[6a]$^{2+}$	^{3}[8a]$^{2+}$	^{3}[9a]$^{2+}$	^{3}[11a]$^{2+}$
**Energy vs. ^{1}[2a]$^{2+}$**	195.0	214.6	241.4	236.4	439.4	467.6
**Ru-O1**	-	1.986	1.989	2.004	2.434	3.362
**Ru-N1**	2.096	3.545	2.133	2.109	-	2.271
**Ru-N2**	2.141	3.180	2.125	2.089	-	2.122
**Ru-N3**	2.092	2.056	2.191	3.435	2.057	-
**Ru-N4**	2.070	2.092	3.437	2.148	2.081	-
**Ru-N5**	2.056	2.159	2.066	2.091	2.085	2.044
**Ru-N6**	2.038	2.079	2.059	2.076	2.063	2.033
**N1-C-C-N2**	180.5	181.4	177.2	181.1	-	177.5
**Mulliken spin (Ru)**	0.829	1.425	0.813	0.802	1.663	1.796

Table S14. The relative energy to the ground state of the starting complex (kJ/mol), the bond distances (Å), the dihedral angle of the straining ligand (°) and the Mulliken spin on ruthenium of the triplet states found in the photodissociation reaction pathways of complex $^1[1a]^{2+}$ in acetonitrile.

	$^3[1a]^{2+}$	$^3[12a]^{2+}$	$^3[14a]^{2+}$	$^3[16a]^{2+}$	$^3[18a]^{2+}$	$^3[19a]^{2+}$
**Energy vs. $^1[1a]^{2+}$**	194.3	195.9	244.2	217.2	297.1	305.3
**Ru-N7**	-	2.025	2.021	2.045	2.045	2.040
**Ru-N1**	2.094	3.776	2.086	2.334	-	2.085
**Ru-N2**	2.099	3.025	2.144	2.156	-	2.098
**Ru-N3**	2.063	2.049	2.394	3.552	2.060	-
**Ru-N4**	2.079	2.052	3.819	2.679	2.029	-
**Ru-N5**	2.066	2.210	2.116	2.060	2.037	2.041
**Ru-N6**	2.081	2.144	2.553	2.053	2.070	2.045
**N1-C-C-N2**	175.3	184.5	186.4	201.0	-	175.8
**Mulliken spin (Ru)**	0.901	1.683	1.652	1.694	1.601	1.673

Table S15. The relative energy to the ground state of the starting complex (kJ/mol), the bond distances (Å), the dihedral angle of the straining ligand (°) and the Mulliken spin on ruthenium of the triplet states found in the photodissociation reaction pathways of complex $^1[2a]^{2+}$ in acetonitrile.

	$^3[2a]^{2+}$	$^3[13a]^{2+}$	$^3[15a]^{2+}$	$^3[17a]^{2+}$	$^3[18a]^{2+}$	$^3[20a]^{2+}$
**Energy vs. $^1[2a]^{2+}$**	195.5	189.5	241.9	217.9	297.1	296.2
**Ru-N7**	-	2.022	2.015	2.032	2.045	2.029
**Ru-N1**	2.091	3.690	2.090	2.259	-	2.055
**Ru-N2**	2.139	3.494	2.154	2.143	-	2.105
**Ru-N3**	2.095	2.054	2.363	3.734	2.060	-
**Ru-N4**	2.69	2.068	3.793	3.098	2.029	-
**Ru-N5**	2.052	2.224	2.110	2.044	2.037	2.033
**Ru-N6**	2.041	2.108	2.597	2.044	2.070	2.055
**N1-C-C-N2**	179.9	182.1	181.7	183.3	-	178.6
**Mulliken spin (Ru)**	0.813	1.738	1.649	1.719	1.601	1.664

Reaction Pathways of First Solvent Molecule

Complex $[1a]^{2+}$ - H_2O – Pathway 1

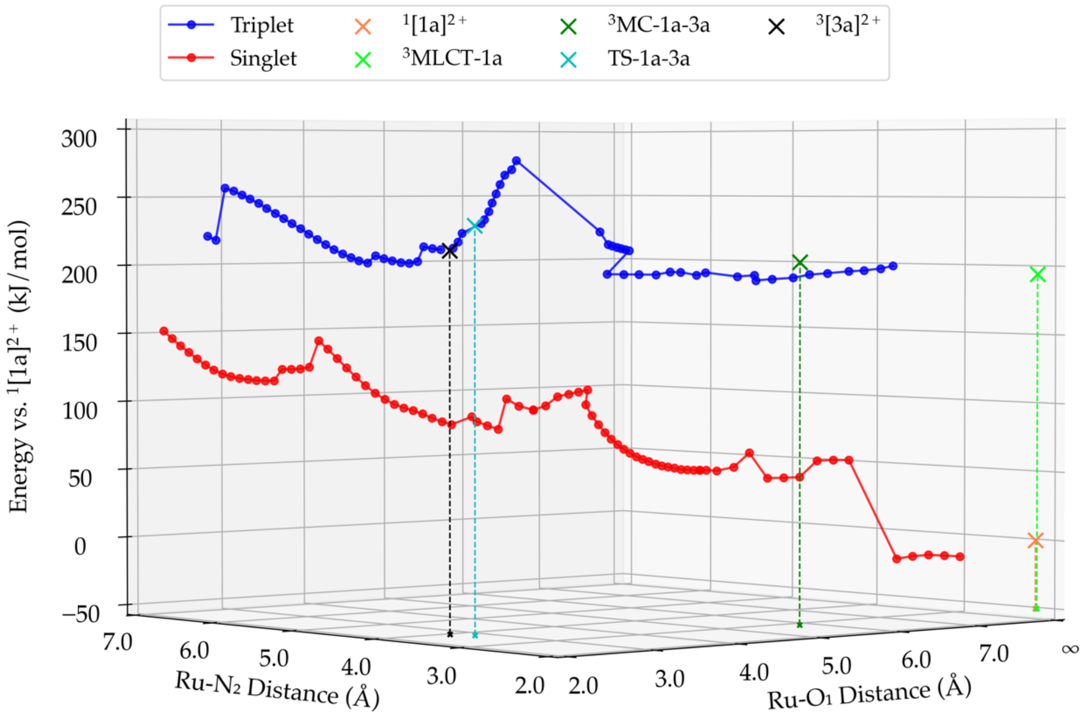

Figure S16. Plot of the linear transits and the obtained mechanistic states for the singlet ground state (red) and for the triplet excited state (blue) for pathway 1 of complex $[1a]^{2+}$ in H_2O. The Ru-O_1 distance is plotted on the x-axis, the Ru-N_2 distance on the y-axis, and the energy relative to the ground state $^1[1a]^{2+}$ is plotted on the z-axis.

Table S16. The relative energy to the ground state of the starting complex (kJ/mol), the bond distances (Å), the dihedral angle of the straining ligand (°) and the Mulliken spin on ruthenium of the states found in reaction pathway 1 of complex $^1[1a]^{2+}$ in water.

	$^1[1a]^{2+}$	^{3}MLCT-1a	^{3}MC-1a-3a	TS-1a-3a	$^3[3a]^{2+}$
**Energy vs. $^1[1a]^{2+}$**	0.0	194.2	205.2	231.7	214.5
**Ru-O1**	-	-	5.048	2.102	2.012
**Ru-N1**	2.108	2.097	2.450	3.847	3.957
**Ru-N2**	2.110	2.094	2.175	3.047	3.246
**Ru-N3**	2.079	2.082	2.153	2.044	2.051
**Ru-N4**	2.074	2.066	2.451	2.060	2.085
**Ru-N5**	2.061	2.080	2.070	2.235	2.184
**Ru-N6**	2.051	2.064	2.076	2.099	2.070
**N1-C-C-N2**	177.6	176.8	175.0	135.5	147.0
**Mulliken spin (Ru)**	-	0.900	1.726	1.742	1.582

Complex $[2a]^{2+}$ - H_2O – Pathway 1

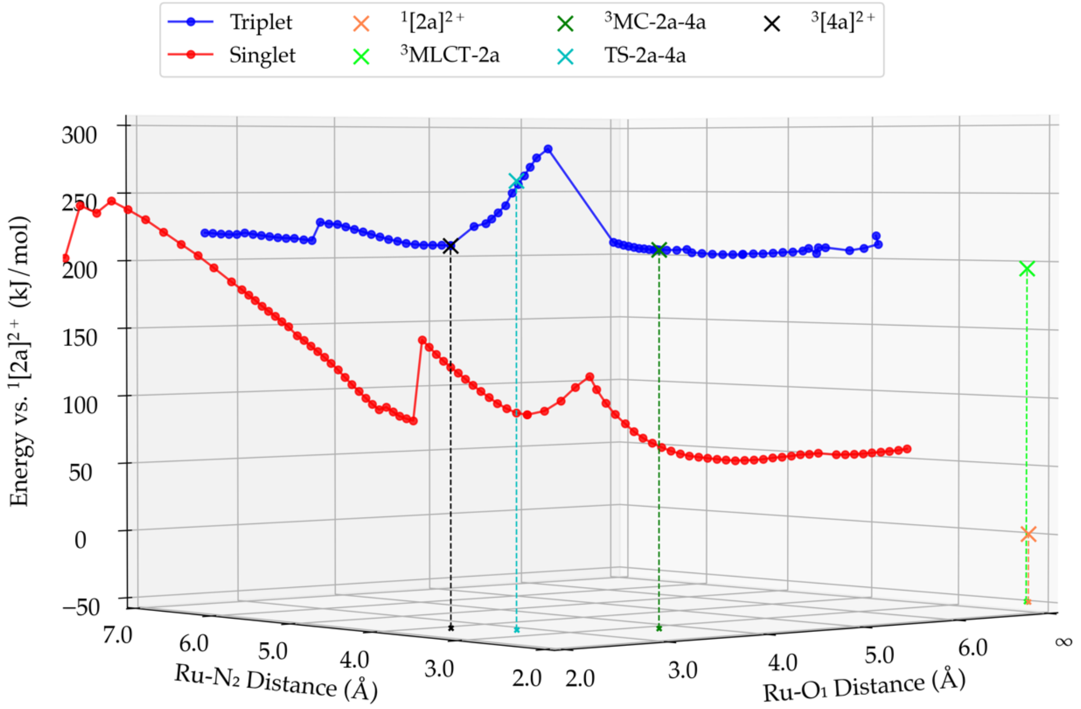

Figure S17. Plot of the linear transits and the obtained mechanistic states for the singlet ground state (red) and for the triplet excited state (blue) for pathway 1 of complex $[2a]^{2+}$ in H_2O. The Ru-O_1 distance is plotted on the x-axis, the Ru-N_2 distance on the y-axis, and the energy relative to the ground state $^1[2a]^{2+}$ is plotted on the z-axis.

Table S17. The relative energy to the ground state of the starting complex (kJ/mol), the bond distances (Å), the dihedral angle of the straining ligand (°) and the Mulliken spin on ruthenium of the states found in reaction pathway 1 of complex $^1[2a]^{2+}$ in water.

	$^1[2a]^{2+}$	^{3}MLCT-2a	^{3}MC-2a-4a	TS-2a-4a	$^3[4a]^{2+}$
**Energy vs. $^1[2a]^{2+}$**	0.0	195.0	212.2	259.7	214.6
**Ru-O1**	-	-	3.115	2.240	1.986
**Ru-N1**	2.116	2.096	2.294	3.091	3.545
**Ru-N2**	2.117	2.141	2.126	2.726	3.180
**Ru-N3**	2.077	2.092	2.117	2.049	2.056
**Ru-N4**	2.067	2.070	2.722	2.037	2.092
**Ru-N5**	2.059	2.056	2.059	2.262	2.159
**Ru-N6**	2.050	2.038	2.146	2.097	2.079
**N1-C-C-N2**	179.4	180.6	178.7	180.5	181.4
**Mulliken spin (Ru)**	-	0.829	1.690	1.653	1.425

Complex $[1a]^{2+}$ - H_2O – Pathway 2

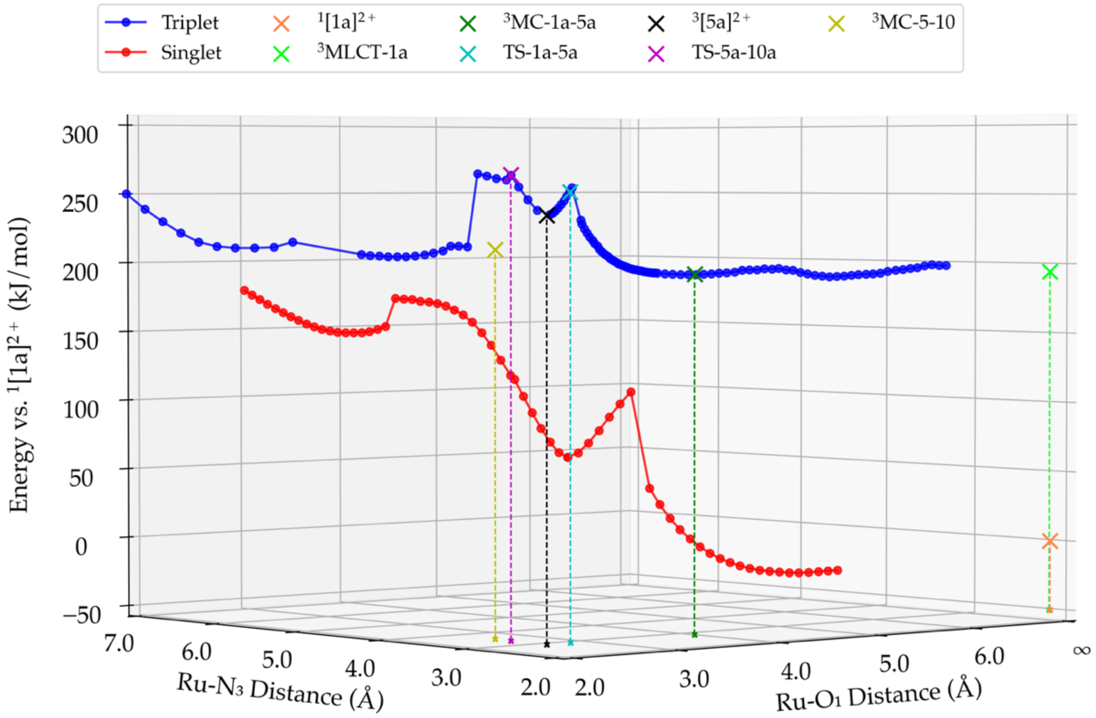

Figure S18. Plot of the linear transits and the obtained mechanistic states for the singlet ground state (red) and for the triplet excited state (blue) for pathway 2 of complex $[1a]^{2+}$ in H_2O. The Ru-O_1 distance is plotted on the x-axis, the Ru-N_3 distance on the y-axis, and the energy relative to the ground state $^1[1a]^{2+}$ is plotted on the z-axis.

Table S18. The relative energy to the ground state of the starting complex (kJ/mol), the bond distances (Å), the dihedral angle of the straining ligand (°) and the Mulliken spin on ruthenium of the states found in reaction pathway 2 of complex $^1[1a]^{2+}$ in water.

	$^1[1a]^{2+}$	^{3}MLCT-1a	^{3}MC-1a-5a	TS-1a-5a	$^3[5a]^{2+}$	TS-5a-10a	^{3}MC-5a-10a
**Energy vs. $^1[1a]^{2+}$**	0.0	194.2	196.3	252.5	237.2	264.0	213.5
**Ru-O1**	-	-	3.357	2.218	1.995	2.008	2.022
**Ru-N1**	2.108	2.097	2.582	2.109	2.121	2.106	2.176
**Ru-N2**	2.110	2.094	2.172	2.127	2.120	2.104	2.088
**Ru-N3**	2.079	2.082	2.128	2.195	2.194	2.608	2.801
**Ru-N4**	2.074	2.066	2.353	3.402	3.434	3.583	3.560
**Ru-N5**	2.061	2.080	2.102	2.060	2.070	2.056	2.078
**Ru-N6**	2.051	2.064	2.077	2.068	2.057	2.074	2.159
**N1-C-C-N2**	177.6	176.8	142.9	161.8	172.6	174.0	178.6
**Mulliken spin (Ru)**	-	0.900	1.710	1.144	0.815	1.038	1.388

Complex $[2a]^{2+}$ - H_2O – Pathway 2

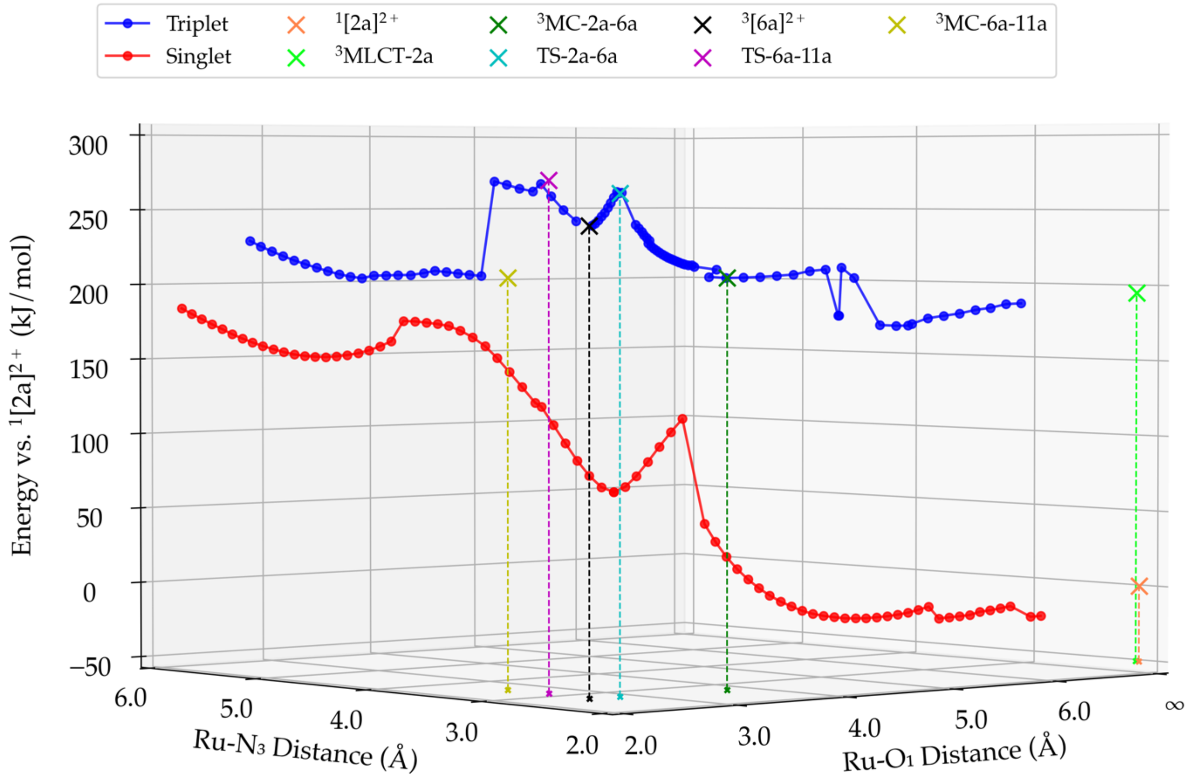

Figure S19. Plot of the linear transits and the obtained mechanistic states for the singlet ground state (red) and for the triplet excited state (blue) for pathway 2 of complex $[2a]^{2+}$ in H_2O. The Ru-O_1 distance is plotted on the x-axis, the Ru-N_3 distance on the y-axis, and the energy relative to the ground state $^1[2a]^{2+}$ is plotted on the z-axis.

Table S19. The relative energy to the ground state of the starting complex (kJ/mol), the bond distances (Å), the dihedral angle of the straining ligand (°) and the Mulliken spin on ruthenium of the states found in reaction pathway 2 of complex $^1[2a]^{2+}$ in water.

	$^1[2a]^{2+}$	^{3}MLCT-2a	^{3}MC-2a-6a	TS-2a-6a	$^3[6a]^{2+}$	TS-6a-11a	^{3}MC-6a-11a
**Energy vs. $^1[2a]^{2+}$**	0.0	195.0	208.4	261.5	241.4	269.5	208.6
**Ru-O1**	-	-	3.150	2.253	1.989	2.045	2.018
**Ru-N1**	2.116	2.096	2.527	2.123	2.133	2.135	2.167
**Ru-N2**	2.117	2.141	2.169	2.139	2.125	2.140	2.083
**Ru-N3**	2.077	2.092	2.139	2.188	2.191	2.576	2.893
**Ru-N4**	2.067	2.070	2.387	3.380	3.437	3.575	3.602
**Ru-N5**	2.059	2.056	2.096	2.049	2.066	2.105	2.069
**Ru-N6**	2.050	2.038	2.086	2.077	2.059	2.234	2.159
**N1-C-C-N2**	179.4	180.5	168.2	174.1	177.2	177.7	178.3
**Mulliken spin (Ru)**	-	0.829	1.698	1.186	0.813	1.449	1.434

Complex [1a]$^{2+}$ - H_2O – Pathway 3

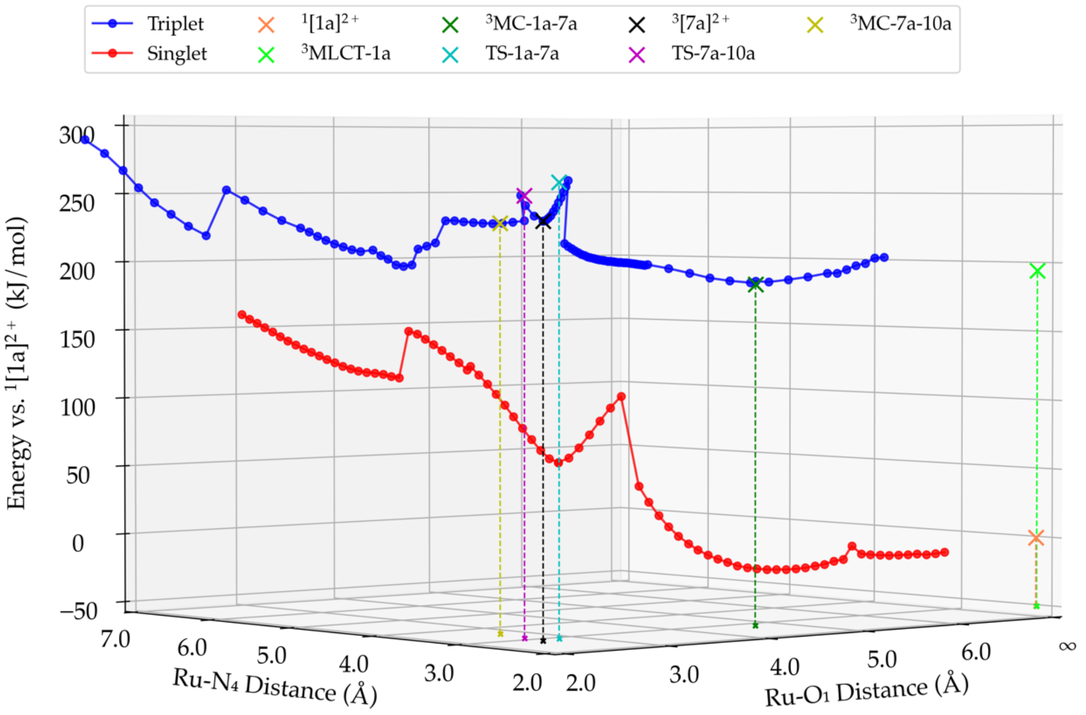

Figure S20. Plot of the linear transits and the obtained mechanistic states for the singlet ground state (red) and for the triplet excited state (blue) for pathway 3 of complex [1a]$^{2+}$ in H_2O. The Ru-O_1 distance is plotted on the x-axis, the Ru-N_4 distance on the y-axis, and the energy relative to the ground state ^{1}[1a]$^{2+}$ is plotted on the z-axis.

Table S20. The relative energy to the ground state of the starting complex (kJ/mol), the bond distances (Å), the dihedral angle of the straining ligand (°) and the Mulliken spin on ruthenium of the states found in reaction pathway 3 of complex ^{1}[1a]$^{2+}$ in water.

	^{1}[1a]$^{2+}$	^{3}MLCT-1a	^{3}MC-1a-7a	TS-1a-7a	^{3}[7a]$^{2+}$	TS-7a-10a	^{3}MC-7a-10a
**Energy vs. ^{1}[1a]$^{2+}$**	0.0	194.2	187.8	259.0	233.1	250.1	200.3
**Ru-O1**	-	-	4.070	2.206	2.007	2.045	2.138
**Ru-N1**	2.108	2.097	2.240	2.281	2.099	2.156	2.243
**Ru-N2**	2.110	2.094	2.090	2.085	2.078	2.127	2.114
**Ru-N3**	2.079	2.082	3.241	3.452	3.447	3.477	3.784
**Ru-N4**	2.074	2.066	2.130	2.210	2.156	2.409	3.923
**Ru-N5**	2.061	2.080	2.063	2.089	2.095	2.067	2.041
**Ru-N6**	2.051	2.064	2.088	2.035	2.077	2.070	2.041
**N1-C-C-N2**	177.6	176.8	175.3	185.3	182.2	199.4	172.4
**Mulliken spin (Ru)**	-	0.900	1.711	1.303	0.803	1.233	1.797

Complex [2a]$^{2+}$ - H_2O – Pathway 3

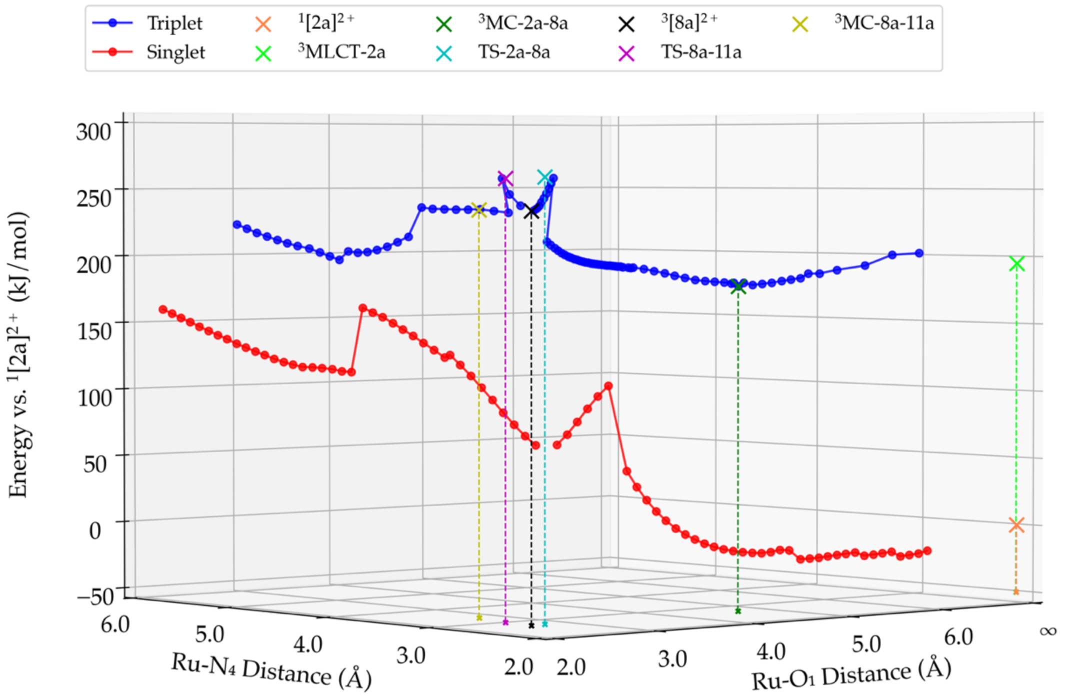

Figure S21. Plot of the linear transits and the obtained mechanistic states for the singlet ground state (red) and for the triplet excited state (blue) for pathway 3 of complex [2a]$^{2+}$ in H_2O. The Ru-O_1 distance is plotted on the x-axis, the Ru-N_4 distance on the y-axis, and the energy relative to the ground state ^{1}[2a]$^{2+}$ is plotted on the z-axis.

Table S21. The relative energy to the ground state of the starting complex (kJ/mol), the bond distances (Å), the dihedral angle of the straining ligand (°) and the Mulliken spin on ruthenium of the states found in reaction pathway 3 of complex ^{1}[2a]$^{2+}$ in water.

	^{1}[2a]$^{2+}$	^{3}MLCT-2a	^{3}MC-2a-8a	TS-2a-8a	^{3}[8a]$^{2+}$	TS-8a-11a	^{3}MC-8a-11a
**Energy vs. ^{1}[2a]$^{2+}$**	0.0	195.0	181.8	259.9	236.4	258.7	197.3
**Ru-O1**	-	-	4.053	2.163	2.004	2.046	2.133
**Ru-N1**	2.116	2.096	2.240	2.229	2.109	2.226	2.72
**Ru-N2**	2.117	2.141	2.091	2.088	2.089	2.136	2.109
**Ru-N3**	2.077	2.092	3.257	3.400	3.435	3.495	3.741
**Ru-N4**	2.067	2.070	2.127	2.174	2.148	2.425	3.934
**Ru-N5**	2.059	2.056	2.064	2.090	2.091	2.061	2.041
**Ru-N6**	2.050	2.038	2.091	2.027	2.076	2.062	2.044
**N1-C-C-N2**	179.4	180.5	179.0	183.4	181.1	185.8	178.7
**Mulliken spin (Ru)**	-	0.829	1.707	1.106	0.802	1.376	1.823

Complex $[1a]^{2+}$ - CH_3CN – Pathway 1

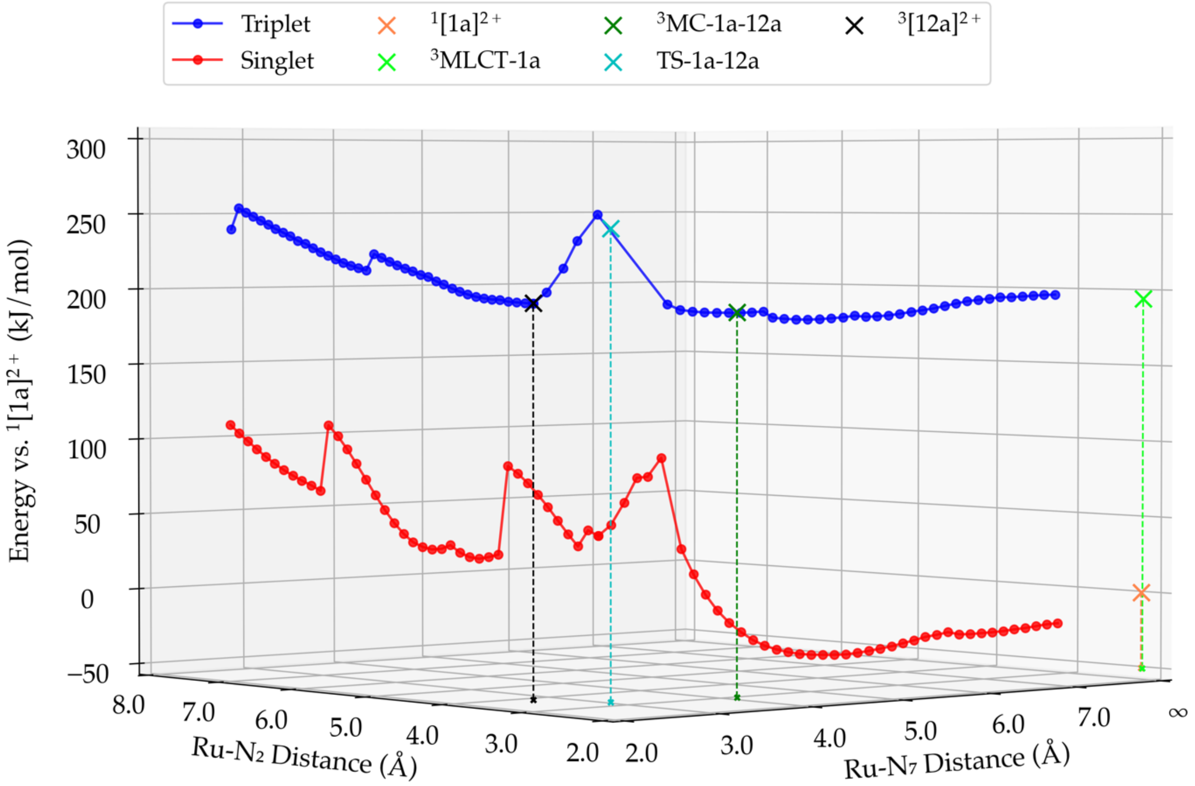

Figure S22. Plot of the linear transits and the obtained mechanistic states for the singlet ground state (red) and for the triplet excited state (blue) for pathway 1 of complex $[1a]^{2+}$ in CH_3CN. The Ru-N_7 distance is plotted on the x-axis, the Ru-N_2 distance on the y-axis, and the energy relative to the ground state $^1[1a]^{2+}$ is plotted on the z-axis.

Table S22. The relative energy to the ground state of the starting complex (kJ/mol), the bond distances (Å), the dihedral angle of the straining ligand (°) and the Mulliken spin on ruthenium of the states found in reaction pathway 1 of complex $^1[1a]^{2+}$ in acetonitrile.

	$^1[1a]^{2+}$	^{3}MLCT-1a	^{3}MC-1a-12a	TS-1a-12a	$^3[12a]^{2+}$
**Energy vs. $^1[1a]^{2+}$**	0.0	194.3	190.0	242.2	195.9
**Ru-N7**	-	-	3.398	2.333	2.025
**Ru-N1**	2.110	2.094	2.301	2.946	3.776
**Ru-N2**	2.106	2.099	2.124	2.436	3.025
**Ru-N3**	2.051	2.063	2.132	2.132	2.049
**Ru-N4**	2.060	2.079	2.695	2.224	2.052
**Ru-N5**	2.074	2.066	2.061	2.284	2.210
**Ru-N6**	2.080	2.081	2.136	2.071	2.144
**N1-C-C-N2**	177.6	175.3	174.2	199.0	203.5
**Mulliken spin (Ru)**	-	0.901	1.682	1.679	1.683

Complex $[2a]^{2+}$ - CH_3CN – Pathway 1

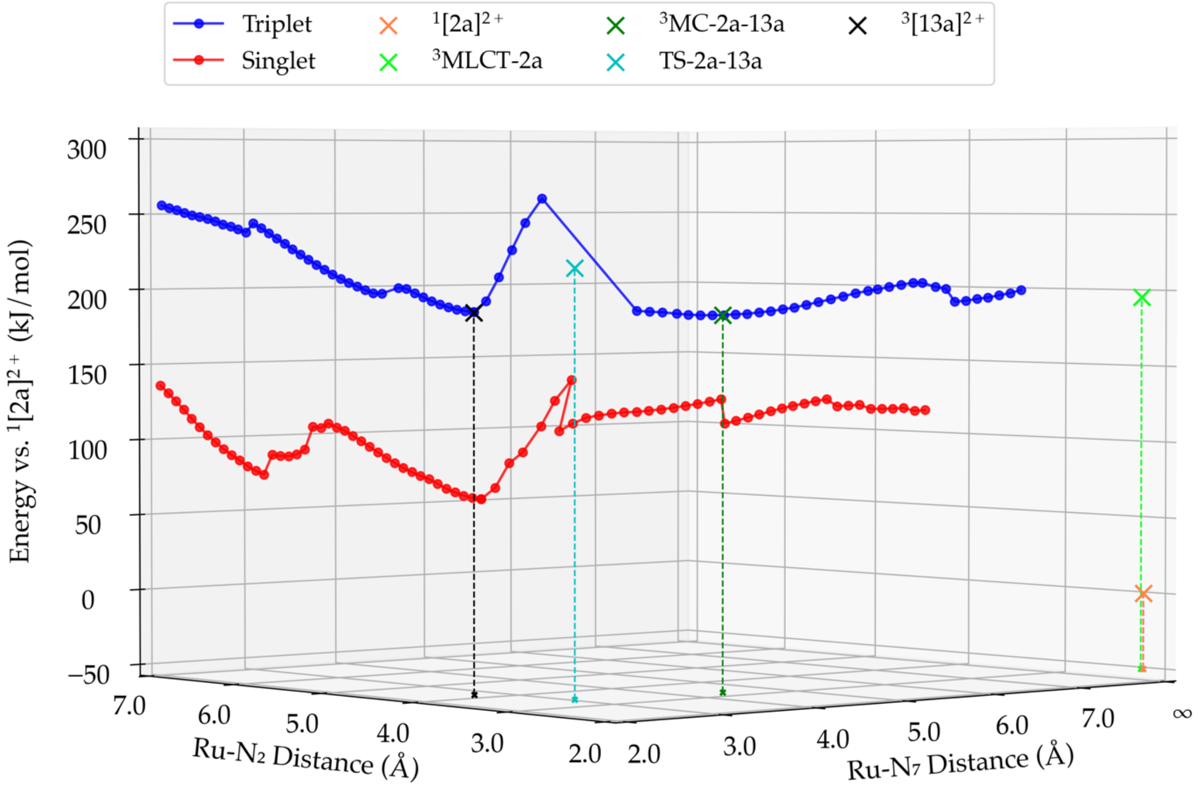

Figure S23. Plot of the linear transits and the obtained mechanistic states for the singlet ground state (red) and for the triplet excited state (blue) for pathway 1 of complex $[2a]^{2+}$ in CH_3CN. The Ru-N_7 distance is plotted on the x-axis, the Ru-N_2 distance on the y-axis, and the energy relative to the ground state $^1[2a]^{2+}$ is plotted on the z-axis.

Table S23. The relative energy to the ground state of the starting complex (kJ/mol), the bond distances (Å), the dihedral angle of the straining ligand (°) and the Mulliken spin on ruthenium of the states found in reaction pathway 1 of complex $^1[1a]^{2+}$ in acetonitrile.

	$^1[2a]^{2+}$	^{3}MLCT-2a	^{3}MC-2a-13a	TS-2a-13a	$^3[13a]^{2+}$
**Energy vs. $^1[2a]^{2+}$**	0.0	195.5	187.6	218.0	189.5
**Ru-N7**	-	-	3.676	2.321	2.022
**Ru-N1**	2.111	2.091	2.186	2.471	3.690
**Ru-N2**	2.115	2.139	2.535	2.743	3.494
**Ru-N3**	2.080	2.095	2.152	2.086	2.054
**Ru-N4**	2.066	2.069	2.417	2.214	2.068
**Ru-N5**	2.055	2.052	2.079	2.341	2.224
**Ru-N6**	2.049	2.041	2.046	2.082	2.108
**N1-C-C-N2**	179.0	179.9	174.0	175.8	182.1
**Mulliken spin (Ru)**	-	0.813	1.696	1.639	1.738

Complex $[1a]^{2+}$ - CH_3CN – Pathway 2

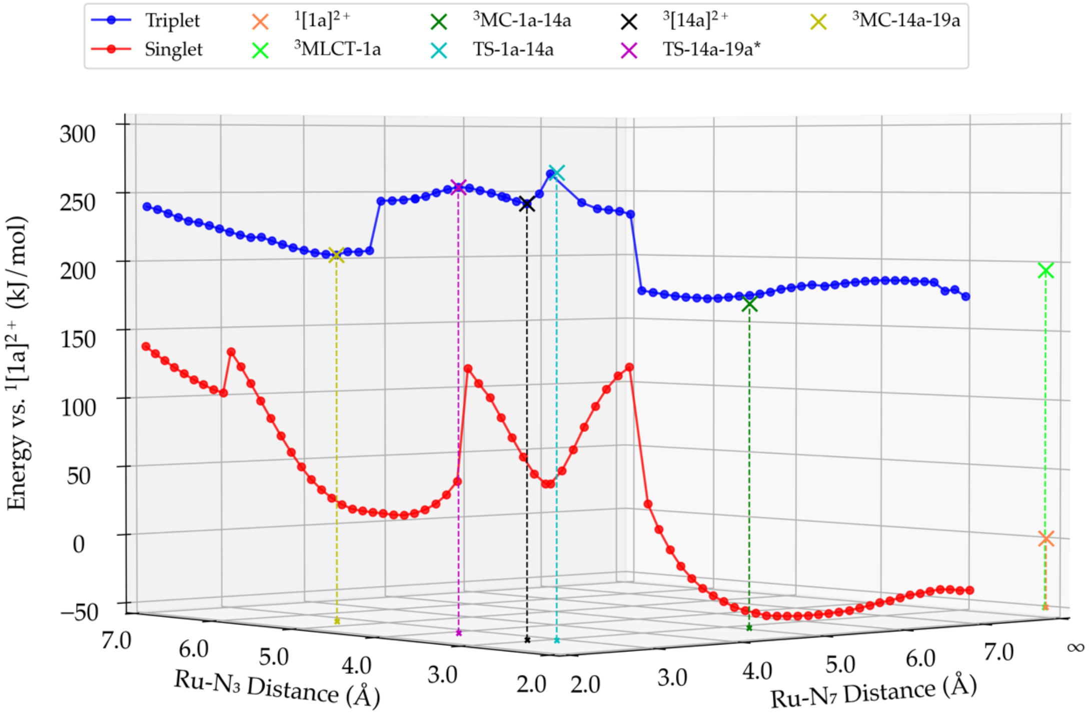

Figure S24. Plot of the linear transits and the obtained mechanistic states for the singlet ground state (red) and for the triplet excited state (blue) for pathway 1 of complex $[1a]^{2+}$ in CH_3CN. The Ru-N7 distance is plotted on the x-axis, the Ru-N3 distance on the y-axis, and the energy relative to the ground state $^1[1a]^{2+}$ is plotted on the z-axis.

Table S24. The relative energy to the ground state of the starting complex (kJ/mol), the bond distances (Å), the dihedral angle of the straining ligand (°) and the Mulliken spin on ruthenium of the states found in reaction pathway 2 of complex $^1[1a]^{2+}$ in acetonitrile.

	$^1[1a]^{2+}$	^{3}MLCT-1a	^{3}MC-1a-14a	TS-1a-14a	$^3[14a]^{2+}$	TS-14a-19a	^{3}MC-14a-19a
**Energy vs. $^1[1a]^{2+}$**	0.0	194.3	174.4	264.9	244.2	255.3	207.1
**Ru-N7**	-	-	4.335	2.176	2.021	2.028	2.021
**Ru-N1**	2.110	2.094	2.601	2.096	2.086	2.095	2.249
**Ru-N2**	2.106	2.099	2.158	2.105	2.144	2.083	2.120
**Ru-N3**	2.051	2.063	2.125	2.216	2.394	3.176	4.602
**Ru-N4**	2.060	2.079	2.346	3.885	3.819	3.969	4.273
**Ru-N5**	2.074	2.066	2.083	2.087	2.116	2.103	2.048
**Ru-N6**	2.080	2.081	2.176	2.083	2.553	2.257	2.062
**N1-C-C-N2**	177.6	175.3	218.1	196.3	186.4	175.7	195.3
**Mulliken spin (Ru)**	-	0.901	1.728	1.122	1.652	1.785	1.756

Complex $[2a]^{2+}$ - CH_3CN – Pathway 2

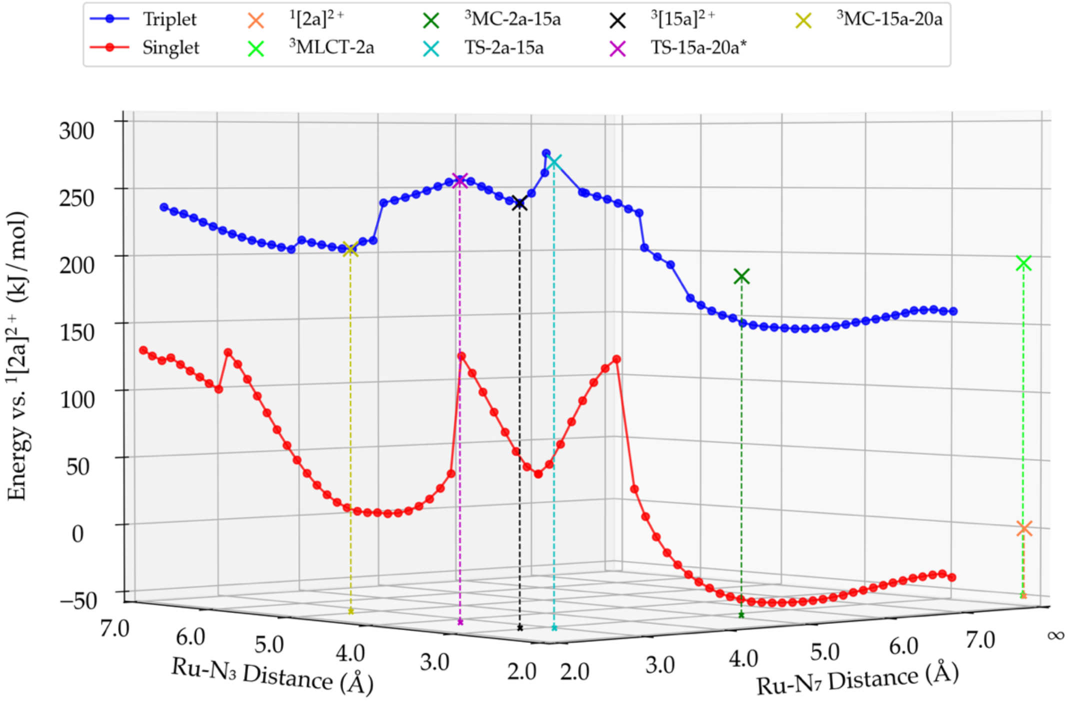

Figure S25. Plot of the linear transits and the obtained mechanistic states for the singlet ground state (red) and for the triplet excited state (blue) for pathway 1 of complex $[2a]^{2+}$ in CH_3CN. The Ru-N7 distance is plotted on the x-axis, the Ru-N3 distance on the y-axis, and the energy relative to the ground state $^1[2a]^{2+}$ is plotted on the z-axis.

Table S25. The relative energy to the ground state of the starting complex (kJ/mol), the bond distances (Å), the dihedral angle of the straining ligand (°) and the Mulliken spin on ruthenium of the states found in reaction pathway 2 of complex $^1[2a]^{2+}$ in acetonitrile.

	$^1[2a]^{2+}$	^{3}MLCT-2a	^{3}MC-2a-15a	TS-2a-15a	$^3[15a]^{2+}$	TS-15a-20a	^{3}MC-15a-20a
**Energy vs. $^1[2a]^{2+}$**	0.0	195.5	189.4	269.6	241.7	256.7	208.0
**Ru-N7**	-	-	4.455	2.259	2.015	2.024	2.017
**Ru-N1**	2.111	2.091	2.473	2.109	2.090	2.108	2.263
**Ru-N2**	2.115	2.139	2.142	2.109	2.154	2.090	2.116
**Ru-N3**	2.080	2.095	2.168	2.213	2.363	3.059	4.355
**Ru-N4**	2.066	2.069	2.434	3.876	3.792	4.004	4.192
**Ru-N5**	2.055	2.052	2.091	2.080	2.110	2.097	2.048
**Ru-N6**	2.049	2.041	2.070	2.107	2.597	2.270	2.062
**N1-C-C-N2**	179.0	179.9	187.0	184.8	181.7	179.8	184.4
**Mulliken spin (Ru)**	-	0.813	1.743	1.295	1.649	1.777	1.768

Complex $[1a]^{2+}$ - CH_3CN – Pathway 3

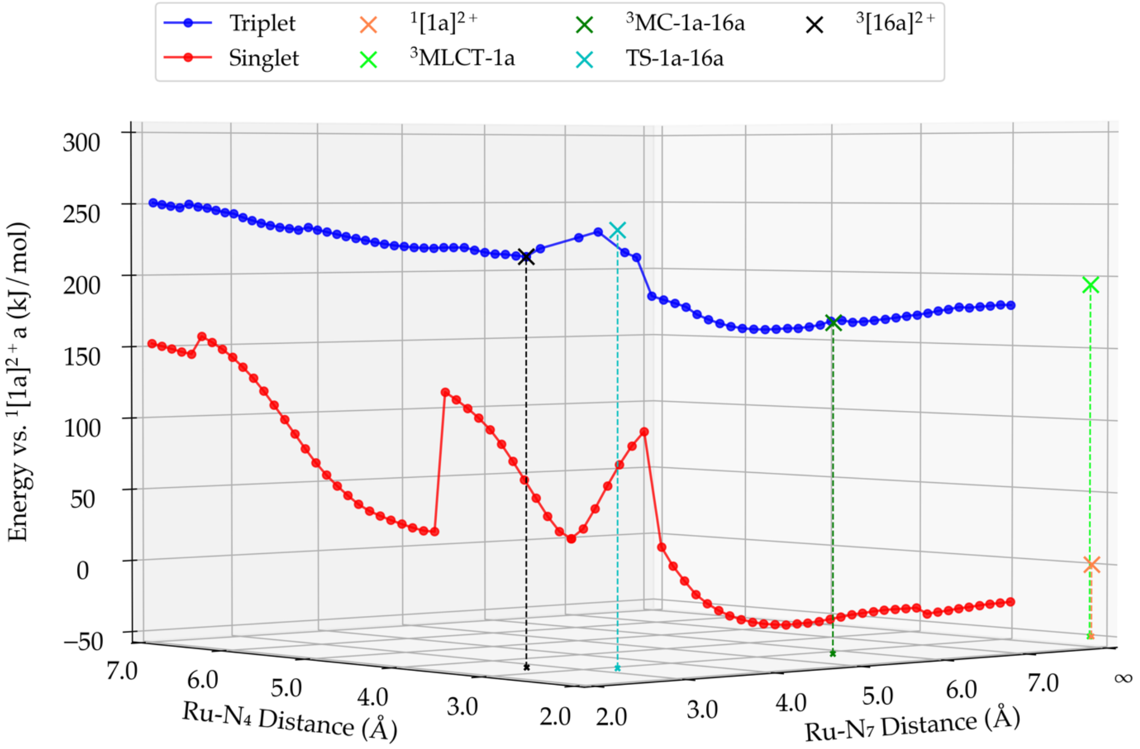

Figure S26. Plot of the linear transits and the obtained mechanistic states for the singlet ground state (red) and for the triplet excited state (blue) for pathway 1 of complex $[1a]^{2+}$ in CH_3CN. The Ru-N_7 distance is plotted on the x-axis, the Ru-N_4 distance on the y-axis, and the energy relative to the ground state $^1[1a]^{2+}$ is plotted on the z-axis.

Table S26. The relative energy to the ground state of the starting complex (kJ/mol), the bond distances (Å), the dihedral angle of the straining ligand (°) and the Mulliken spin on ruthenium of the states found in reaction pathway 3 of complex $^1[1a]^{2+}$ in acetonitrile.

	$^1[1a]^{2+}$	^{3}MLCT-1a	^{3}MC-1a-16a	TS-1a-16a	$^3[16a]^{2+}$
**Energy vs. $^1[1a]^{2+}$**	0.0	194.3	171.6	234.3	217.2
**Ru-N7**	-	-	4.944	2.577	2.045
**Ru-N1**	2.110	2.094	2.163	2.334	2.334
**Ru-N2**	2.106	2.099	2.721	2.094	2.156
**Ru-N3**	2.051	2.063	2.070	3.389	3.552
**Ru-N4**	2.060	2.079	2.145	2.222	2.679
**Ru-N5**	2.074	2.066	2.258	2.072	2.060
**Ru-N6**	2.080	2.081	2.091	2.135	2.053
**N1-C-C-N2**	177.6	175.3	138.9	197.6	201.0
**Mulliken spin (Ru)**	-	0.901	1.722	1.752	1.694

Complex $[2a]^{2+}$ - CH_3CN – Pathway 3

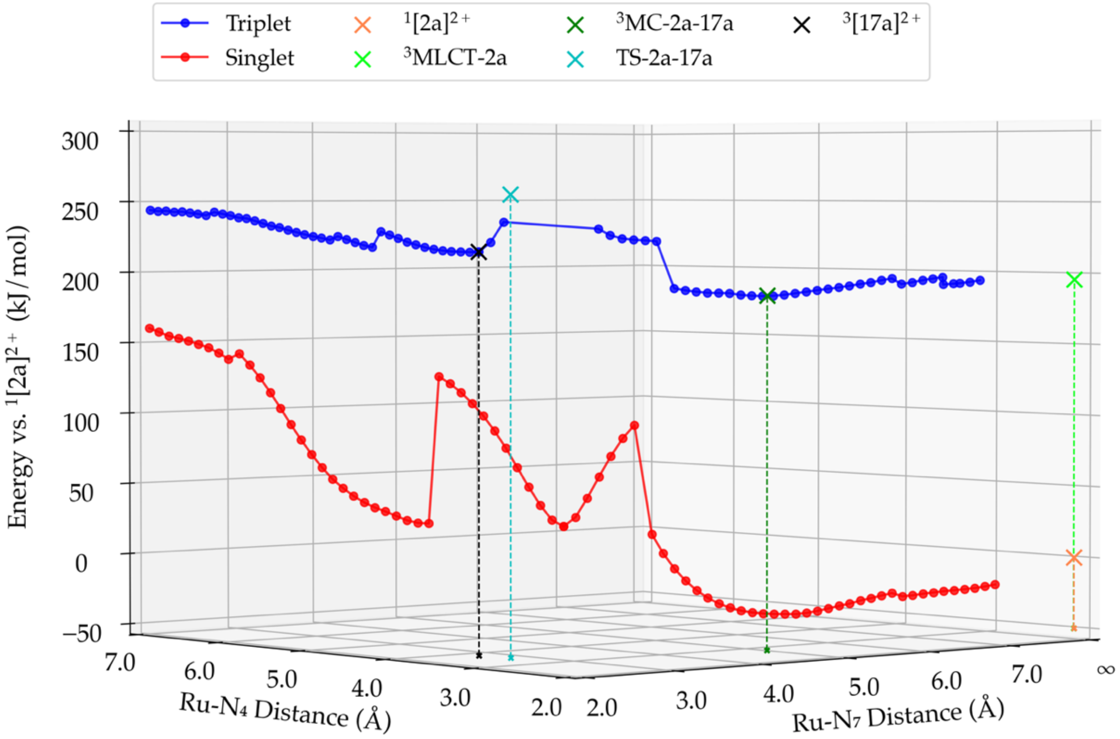

Figure S27. Plot of the linear transits and the obtained mechanistic states for the singlet ground state (red) and for the triplet excited state (blue) for pathway 1 of complex $[2a]^{2+}$ in CH_3CN. The Ru-N_7 distance is plotted on the x-axis, the Ru-N_4 distance on the y-axis, and the energy relative to the ground state $^1[2a]^{2+}$ is plotted on the z-axis.

Table S27. The relative energy to the ground state of the starting complex (kJ/mol), the bond distances (Å), the dihedral angle of the straining ligand (°) and the Mulliken spin on ruthenium of the states found in reaction pathway 3 of complex $^1[2a]^{2+}$ in acetonitrile.

	**^{1}[2a]$^{2+}$**	**^{3}MLCT-2a**	**^{3}MC-2a-17a**	**TS-2a-17a**	**^{3}[17a]$^{2+}$**
**Energy vs. ^{1}[2a]$^{2+}$**	0.0	195.5	188.1	255.9	217.9
**Ru-N7**	-	-	4.255	2.084	2.032
**Ru-N1**	2.111	2.091	2.147	2.386	2.259
**Ru-N2**	2.115	2.139	2.418	2.128	2.143
**Ru-N3**	2.080	2.095	2.073	2.726	3.734
**Ru-N4**	2.066	2.069	2.088	2.798	3.098
**Ru-N5**	2.055	2.052	2.502	2.061	2.044
**Ru-N6**	2.049	2.041	2.155	2.064	2.044
**N1-C-C-N2**	179.0	179.9	174.2	183.4	183.3
**Mulliken spin (Ru)**	-	0.813	1.748	1.642	1.719

Singlet and Triplet Potential Energy Surfaces

Complex [1a]$^{2+}$ - H_2O

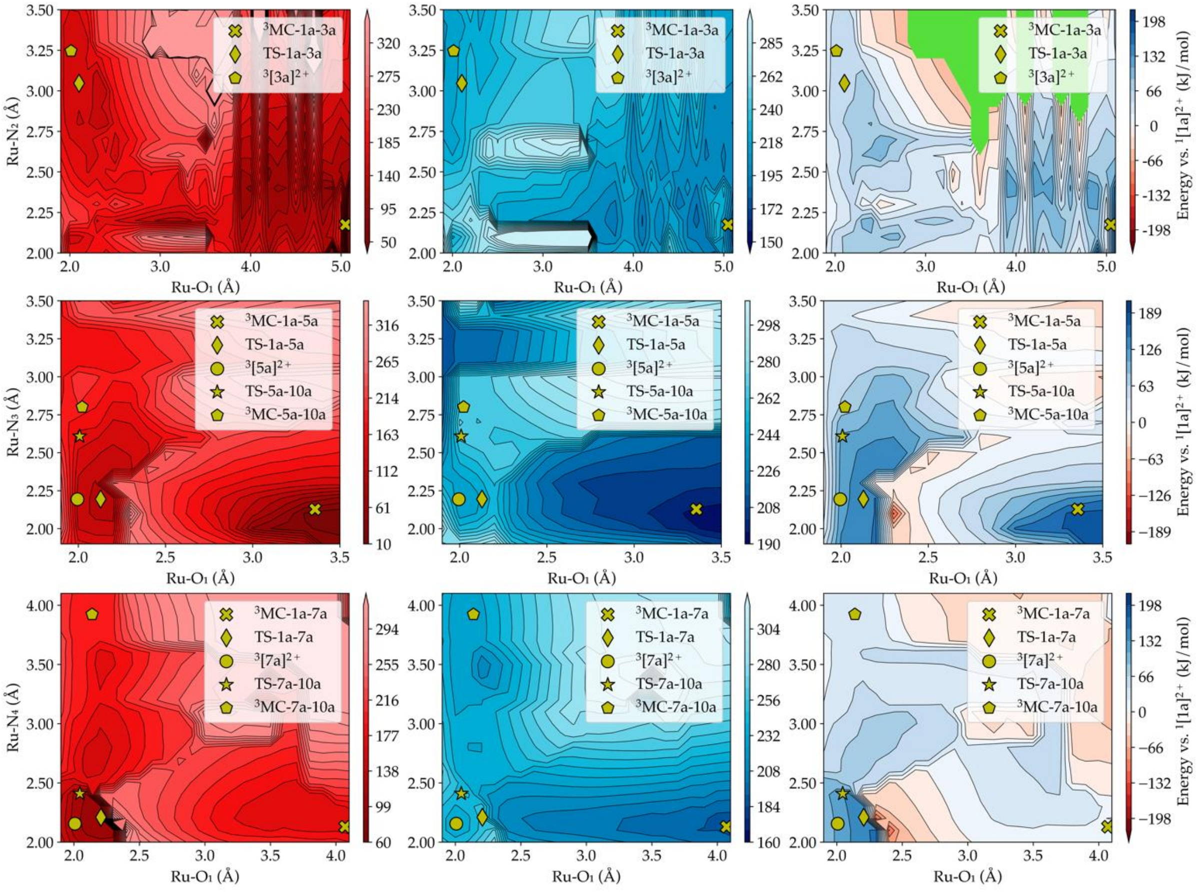

Figure S28. The singlet, triplet and difference in energy between these two surfaces of pathway 1-3 of complex [1a]$^{2+}$ in water.

Complex $[2a]^{2+}$ - H_2O

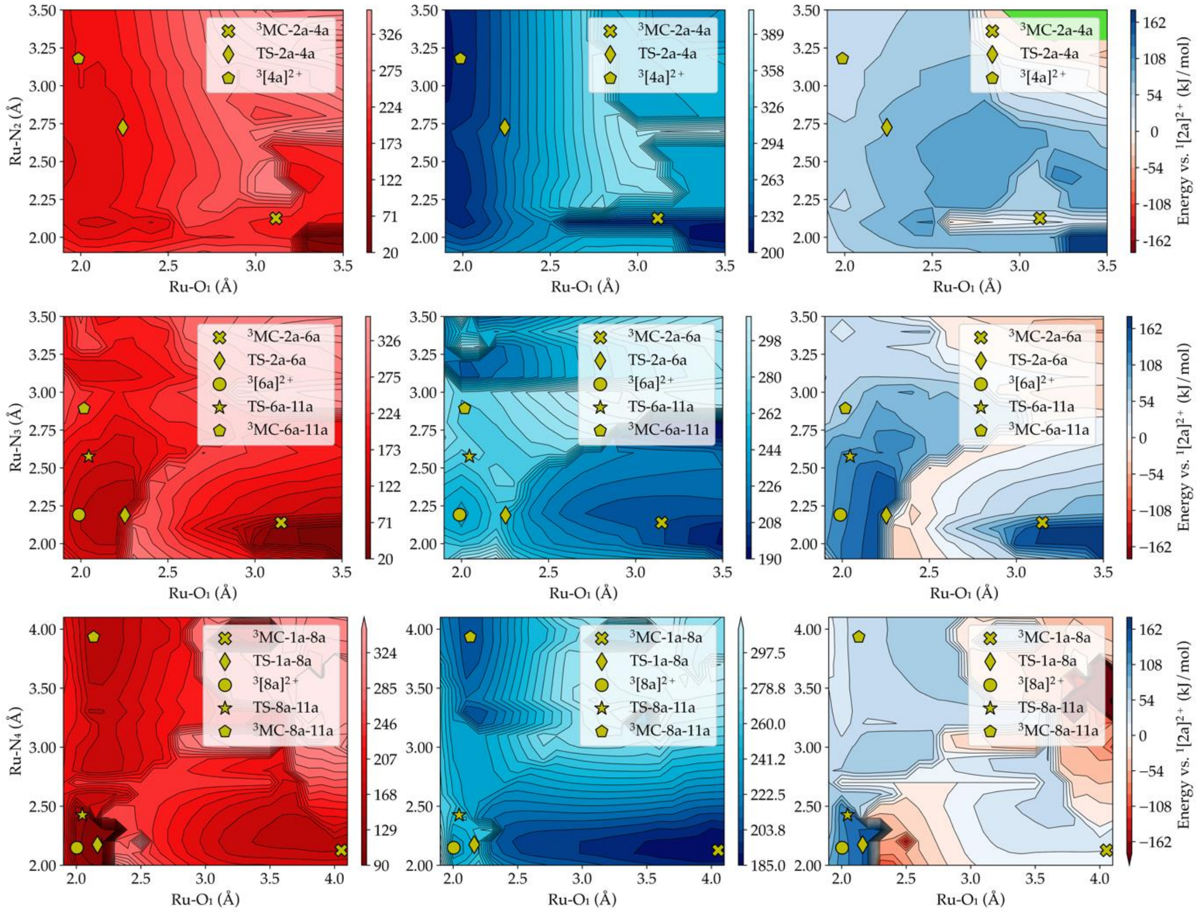

Figure S29. The singlet, triplet and difference in energy between these two surfaces of pathway 1-3 of complex $[2a]^{2+}$ in water.

Complex $[1a]^{2+}$ - CH_3CN

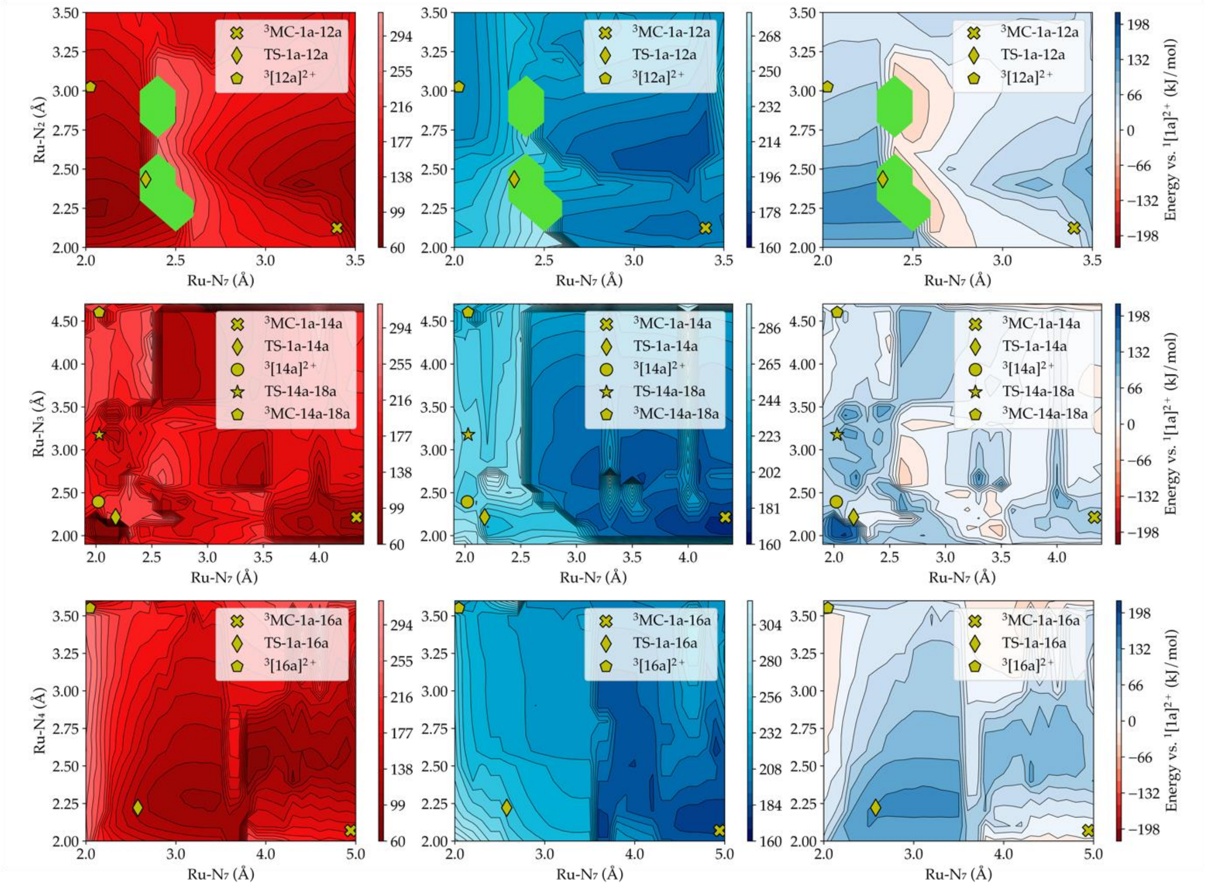

Figure S30. The singlet, triplet and difference in energy between these two surfaces of pathway 1-3 of complex $[1a]^{2+}$ in acetonitrile.

References

(1) Seok, W. K.; Meyer, T. J. Mechanism of Oxidation of Benzaldehyde by Polypyridyl Oxo Complexes of Ru(IV). *Inorg. Chem.* **2005**, *44* (11), 3931–3941. https://doi.org/10.1021/ic040119z.

(2) Howerton, B. S.; Heidary, D. K.; Glazer, E. C. Strained Ruthenium Complexes Are Potent Light-Activated Anticancer Agents. *J. Am. Chem. Soc.* **2012**, *134* (20), 8324–8327. https://doi.org/10.1021/ja3009677.

(3) Azar, D.; Audi, H.; Farhat, S.; El Sibai, M.; Abi-Habib, R.; Khnayzer, R. S. Phototoxicity of Strained Ru(II) Complexes: Is It the Metal Complex or the Dissociating Ligand? *Dalton Trans.* **2017**, *46* (35), 11529–11532. https://doi.org/10.1039/C7DT02255G.

(4) Collins, J. E.; Lamba, J. J. S.; Love, J. C.; McAlvin, J. E.; Ng, C.; Peters, B. P.; Wu, X.; Fraser, C. L. Ruthenium(II) α-Diimine Complexes with One, Two, and Three 4,4‘-Bis(Hydroxymethyl)-2,2‘-Bipyridine and 4,4‘-Bis(Chloromethyl)-2,2‘-Bipyridine Ligands: Useful Starting Materials for Further Derivatization. *Inorg. Chem.* **1999**, *38* (9), 2020–2024. https://doi.org/10.1021/ic981102h.

(5) Laemmel, A.-C.; Collin, J.-P.; Sauvage, J.-P. Efficient and Selective Photochemical Labilization of a Given Bidentate Ligand in Mixed Ruthenium(II) Complexes of the Ru(Phen)2L2+ and Ru(Bipy)2L2+ Family (L = Sterically Hindering Chelate). *Eur. J. Inorg. Chem.* **1999**, *1999* (3), 383–386. https://doi.org/10.1002/(SICI)1099-0682(199903)1999:3<383::AID-EJIC383>3.0.CO;2-9.

(6) Yoshikawa, N.; Yamabe, S.; Sakaki, S.; Kanehisa, N.; Inoue, T.; Takashima, H. Transition States of the 3MLCT to 3MC Conversion in Ru(Bpy)2(Phen Derivative)2+ Complexes. *J. Mol. Struct.* **2015**, *1094*, 98–108. http://dx.doi.org/10.1016/j.molstruc.2015.04.011.

(7) DeRosa, M. C.; Crutchley, R. J. Photosensitized Singlet Oxygen and Its Applications. *Coord. Chem. Rev.* **2002**, *233*, 351–371. http://dx.doi.org/10.1016/S0010-8545(02)00034-6.

(8) Garcìa-Fresnadillo, D.; Georgiadou, Y.; Orellana, G.; Braun, A. M.; Oliveros, E. Singlet-Oxygen (1Δg) Production by Ruthenium(II) Complexes Containing Polyazaheterocyclic Ligands in Methanol and in Water. *Helv. Chim. Acta* **1996**, *79* (4), 1222–1238. https://doi.org/10.1002/hlca.19960790428.

(9) Suzuki, K.; Kobayashi, A.; Kaneko, S.; Takehira, K.; Yoshihara, T.; Ishida, H.; Shiina, Y.; Oishi, S.; Tobita, S. Reevaluation of Absolute Luminescence Quantum Yields of Standard Solutions Using a Spectrometer with an Integrating Sphere and a Back-Thinned CCD Detector. *Phys. Chem. Chem. Phys.* **2009**, *11* (42), 9850–9860. https://doi.org/10.1039/B912178A.
